# The jojoba lipid droplet protein LDAP1 facilitates the packaging of wax esters into lipid droplets

**DOI:** 10.1093/plcell/koaf115

**Published:** 2025-08-01

**Authors:** Payton Whitehead, Saad Raza, Magdalena Miklaszewska, Ellen Hornung, Cornelia Herrfurth, Rohith Nadella, Alyssa Clews, Nathan M Doner, John M Dyer, Robert Mullen, Ivo Feussner, Josh V Vermaas, Kent D Chapman

**Affiliations:** BioDiscovery Institute and Department of Biological Sciences, University of North Texas, Denton, TX 76203, USA; MSU-DOE Plant Research Laboratory and Department of Biochemistry and Molecular Biology, Michigan State University, East Lansing, MI 48823, USA; Department of Plant Experimental Biology and Biotechnology, University of Gdańsk, Wita Stwosza 59, Gdańsk 80-308, Poland; Department of Plant Biochemistry, Albrecht-von-Haller-Institute for Plant Sciences and Göttingen Center for Molecular Biosciences (GZMB), University of Göttingen, Göttingen, Germany; Department of Plant Biochemistry, Albrecht-von-Haller-Institute for Plant Sciences and Göttingen Center for Molecular Biosciences (GZMB), University of Göttingen, Göttingen, Germany; Department of Plant Biochemistry, Albrecht-von-Haller-Institute for Plant Sciences and Göttingen Center for Molecular Biosciences (GZMB), University of Göttingen, Göttingen, Germany; MSU-DOE Plant Research Laboratory and Department of Biochemistry and Molecular Biology, Michigan State University, East Lansing, MI 48823, USA; Department of Molecular and Cellular Biology, University of Guelph, Guelph, ON, Canada N1G 2W1; Department of Molecular and Cellular Biology, University of Guelph, Guelph, ON, Canada N1G 2W1; U.S. Department of Agriculture, Agricultural Research Service, Albany, CA 94710, USA; Department of Molecular and Cellular Biology, University of Guelph, Guelph, ON, Canada N1G 2W1; Department of Plant Biochemistry, Albrecht-von-Haller-Institute for Plant Sciences and Göttingen Center for Molecular Biosciences (GZMB), University of Göttingen, Göttingen, Germany; MSU-DOE Plant Research Laboratory and Department of Biochemistry and Molecular Biology, Michigan State University, East Lansing, MI 48823, USA; BioDiscovery Institute and Department of Biological Sciences, University of North Texas, Denton, TX 76203, USA

## Abstract

Jojoba (*Simmondsia chinensis*) is a desert shrub with an unusual capacity to store liquid wax esters (WEs) in its seeds instead of triacylglycerols (TAGs) like most oilseed crops. To examine the factors that are important for WE compartmentalization in jojoba, we reconstituted WE biosynthesis and packaging in the leaves of *Nicotiana benthamiana*. Using this system, we screened jojoba proteins for their ability to support lipid droplet (LD) formation. A specific LIPID DROPLET-ASSOCIATED PROTEIN (LDAP) isoform, ScLDAP1, was identified as a key factor in the efficient compartmentalization of WEs in plant cells. LDAP1 isoforms from other plants (e.g. *Arabidopsis thaliana* [AtLDAP1]) did not support WE partitioning from the endoplasmic reticulum into LDs, although both AtLDAP1 and ScLDAP1 were targeted specifically to LD monolayer surfaces. ScLDAP1-mediated selective, efficient WE partitioning was facilitated by an amphipathic α-helix near its C-terminus, and mutational analysis identified 1 amino acid residue within this helix that was both necessary and sufficient for proper WE packaging into cytoplasmic LDs. Taken together, our results provide a mechanistic link between the biosynthesis and storage of WEs in plant cells, and will inform future biotechnology strategies for the efficient packaging of various neutral lipid types as demonstrated here for WEs in transgenic seeds.

## Teaser

The lipid droplet (LD) protein LDAP1 facilitates wax ester partitioning from the endoplasmic reticulum to cytoplasmic LDs in plant cells.

## Introduction

Storing lipids is a universal and evolutionarily conserved feature across organisms found in all of biology. While storage mechanisms may differ between prokaryotes and eukaryotes, 1 common theme is that hydrophobic molecules are packaged into subcellular organelles known as lipid droplets (LDs) (Birsoy et al. 2013; de Vries and Ischebeck 2020; Guzha et al. 2023). In plants, LDs are generally small in size (i.e. ∼1.0 *µ*m) and composed of a neutral lipid core surrounded by a phospholipid monolayer derived from the endoplasmic reticulum (ER) membrane during a directional budding process. In many organisms, the primary neutral lipids that are stored in LDs are triacylglycerols (TAGs) and steryl esters (SEs), but in some organisms the core of the LDs can vary, including wax esters (WEs), retinyl esters, or terpenes (Thiam and Beller 2017; Laibach et al. 2018; Sadre et al. 2019; Lundquist et al. 2020; Sturtevant et al. 2020; Antoine et al. 2023). Regardless of their composition, LDs were originally thought of as merely storage depots for lipophilic compounds, but have since been found to be linked to an array of functions including membrane trafficking, lipid signaling, and membrane remodeling during responses to stress and/or developmental changes (Ischebeck et al. 2020; Lundquist et al. 2020; Guzha et al. 2023).

While the overall mechanisms underlying LD biogenesis are still being uncovered, super-resolution microscopy and electron microscopy tomography have revealed that LD biogenesis begins at the ER membrane bilayer (Gao et al. 2019; Jackson 2019; Nettebrock and Bohnert 2020). Various proteins, including those involved in the final steps of neutral lipid synthesis, as well as various structural proteins specific to LD formation and maintenance, localize to distinct regions within the ER (Gao et al. 2019; Nettebrock and Bohnert 2020; Kumari et al. 2023). It is at these sites that neutral lipid synthesizing enzymes (e.g. acyltransferases) function to increase the local concentrations of neutral lipids, such as TAGs within the ER bilayer (Choudhary and Schneiter 2020; Henne et al. 2020). Once a specific concentration is reached, these non-bilayer-forming lipids demix and coalesce into what is referred to as a “lipid lens” between the 2 leaflets of the phospholipid bilayer (Gao et al. 2019; Jackson 2019; Kumari et al. 2023). After the establishment of the lipid lens, various ER membrane-bound structural proteins (referred to as Class 1-type LD proteins) localize to and promote the protrusion of the lipid lens into the cytoplasm (Pyc et al. 2017b; Olzmann and Carvalho 2019; Scholz et al. 2022). In addition to the membrane-bound proteins, soluble proteins from the cytoplasm (referred to as Class 2 type LD proteins) associate with and assist in stabilizing the growing, nascent LD (Chapman et al. 2019; Olzmann and Carvalho 2019; Scholz et al. 2022). Although a consensus LD targeting sequence has yet to be identified, most Class 2-type LD proteins utilize an amphipathic α-helix (or helices) to associate with the LD surface (Čopič et al. 2018; Prévost et al. 2018; Chorlay and Thiam 2020; Dhiman et al. 2020). While the role(s) of these LD structural proteins are still poorly understood, it is thought that some assist in the mitigation of membrane packaging defects, promotion of membrane curvature, and/or LD stabilization and prevention of LD-LD fusion (Santinho et al. 2020; Thiam and Ikonen 2021; Guzha et al. 2023). In addition, other proteins presumably promote the emergence and vectorial “budding” of the LD away from the ER surface, yielding a fully-formed, nascent LD that may be released into the cytoplasm via a scission event or may remain physically connected to the ER (Guzha et al. 2023).

Across eukaryotes, some homologous proteins seem to be conserved in their involvement in LD biogenesis. One such protein, SEIPIN, which is an ER membrane protein, has been shown to be important for site determination of LD formation, the directional emergence of the LD toward the cytoplasm, and for determining LD size and number (Cartwright and Goodman 2012; Cai et al. 2015; Chung et al. 2019). Other proteins such as the Class 2-type perilipin proteins in mammals, also influence LD size and numbers, and also are involved in resolving packing defects at the surface of nascent LDs (Giménez-Andrés et al. 2018; Prévost et al. 2018; Braun and Swanson 2022). Although no obvious perilipin homologs exist in plants (Chapman et al. 2019), it seems that analogous proteins have evolved similar functions, including the oleosins and LD-associated proteins (LDAPs), to support the emergence and stability of LDs in plant cells (Horn et al. 2013; Gidda et al. 2016; Pyc et al. 2017b; Huang 2018; Chapman et al. 2019; de Vries and Ischebeck 2020).

While the majority of plant LD biogenesis studies have focused primarily on the storage of TAGs, a number of plant species accumulate other types of lipid compounds, such as WEs, terpenes, terpene-esters, or TAGs containing unusual fatty acids (Hayes et al. 1995; Smith et al. 2013; Sturtevant et al. 2020; Lanier et al. 2023). Many of these compounds are of economic interest due to their potential usage in high-value nutraceutical or industrial applications (Romsdahl et al. 2019; Al Sulaimi et al. 2023). However, they are often produced in plants with low yields or other agronomic limitations. Investigations into the underlying mechanisms responsible for the synthesis and accumulation of these lipids have revealed that differential expression and/or evolutionarily diverged forms of lipid biosynthetic enzymes are often involved in their production (Clews et al. 2023). Numerous attempts have been made to express these genes in higher yielding platform crops, but unfortunately, amounts of the desired lipids are often well below that needed for commercial applications and sometimes have negative effects on overall yield or development. For example, studies attempting to over-accumulate economically valuable lipid compounds, such as patchoulol and WEs, in transgenic plants had some detrimental impacts on seed germination, seedling establishment, and plant growth and development (Zhan et al. 2014; Iven et al. 2016; Domergue and Miklaszewska 2022).

In an effort to identify factors that reduce the negative effects associated with WE production and accumulation, we previously performed a tissue-specific proteomic and transcriptomic analysis of the WE-accumulating seed tissues of jojoba (*Simmondsia chinensis*) (Sturtevant et al. 2020). These analyses revealed that along with gene transcripts encoding proteins well known to be involved in WE synthesis, such as wax synthase (WS), fatty acyl-CoA reductase (FAR), and fatty acid elongase (FAE), transcripts for a number of LD protein homologs were preferentially expressed in WE-accumulating seed tissues, with corresponding proteins accumulated on the LDs. Based on these results, we hypothesized that jojoba has evolved 1 or more LD packaging protein(s) to be specific or required for the efficient packaging of WEs into LDs. Here, we tested several of these LDAPs from jojoba for their capacity to support WE packaging in a transient, heterologous system, namely *Nicotiana benthamiana* leaves, and complemented these studies with molecular dynamic simulations (MDSs) and mutagenesis studies to develop a mechanistic explanation for WE recognition and packing. Among the proteins examined, jojoba LDAP1 (ScLDAP1) uniquely promoted the partitioning of WEs from the ER into LDs, and 1 amino acid residue in an amphipathic α-helix near the C-terminus of the ScLDAP1 protein was essential for the efficient packaging of WEs into LDs. ScLDAP1 not only promoted the packaging of WEs into LDs in leaves but also corrected the defective compartmentation of WEs and supported improved germination in transgenic Arabidopsis seeds designed to synthesize and accumulate jojoba-like WEs.

## Results

### Newly synthesized, very-long-chain WEs accumulate in the ER of *N. benthamiana* leaves and their partitioning into LDs is ameliorated by co-expression of ScLDAP1

**Figure 1.**
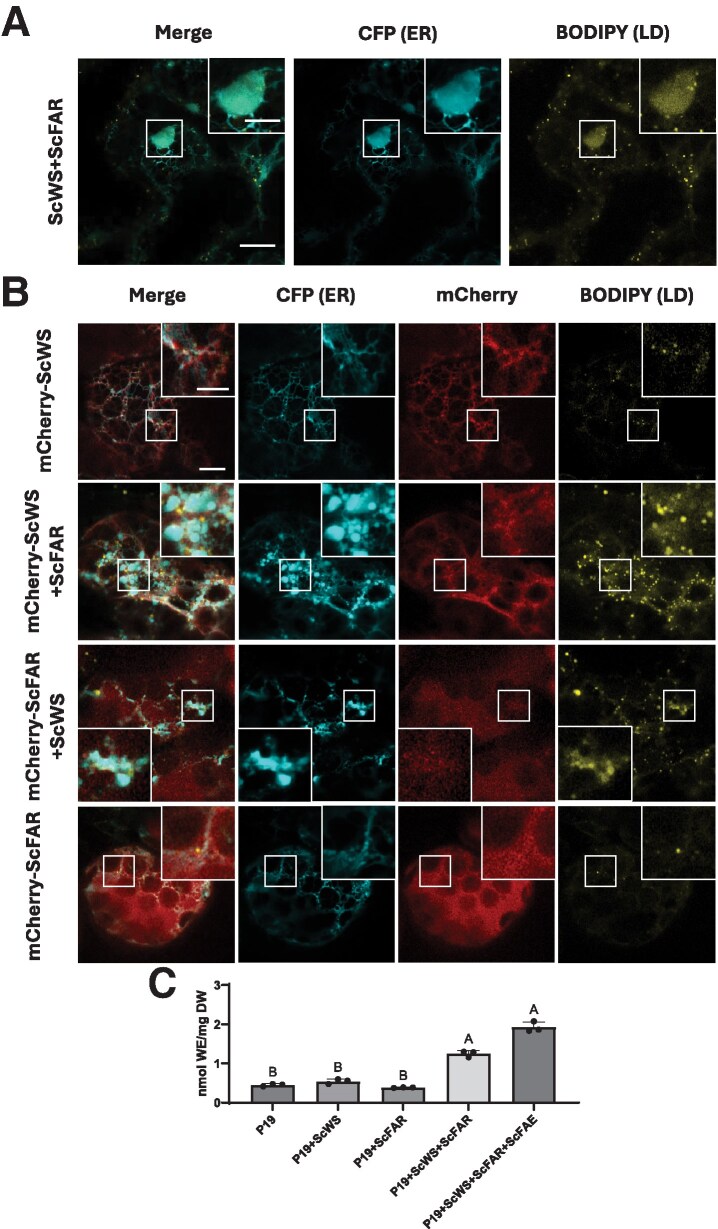
Accumulation of WEs in the leaf tissue of *N. benthamiana* induces ER defects. **A)** Representative confocal laser scanning microscopic (CLSM) images (z-sections) of infiltrated *N. benthamiana* leaves transiently producing ScWS, ScFAR, and a CFP-tagged ER marker (CFP-HDEL). Samples were stained with BODIPY (493/503) to visualize neutral lipids. Higher magnification Inserts highlight regions of WE-induced ER defects. Scale bars represent 10 *µ*m for the lower magnification images, and 5 *µ*m for magnified inserts. CFP-HDEL, cyan fluorescence; BODIPY (493/503), yellow fluorescence. For each transient expression experiment, *A. tumefaciens* harboring the P19 viral suppressor construct was added to suppress transgene silencing. All samples were stained and imaged (Zeiss LSM710 confocal laser scanning microscope, fitted with AIRYSCAN attachment) at 4 d post infiltration. Transient production of ScWS and ScFAR induced large swollen regions of ER defects presumed to be caused by improper release of WEs from the ER. **B)** Representative CLSM images (z-sections) of infiltrated *N. benthamiana* leaves transiently producing CFP-HDEL simultaneously with the following: mCherry-tagged ScWS alone, mCherry-tagged ScWS and untagged ScFAR, mCherry-tagged ScFAR and untagged ScWS, or, mCherry-ScFAR alone. Samples were stained with BODIPY (493/503) to visualize neutral lipids. CFP-HDEL, cyan; BODIPY (493/503), yellow; mCherry, magenta. Higher magnification inserts highlight regions of WE-induced ER defects, and “normal” reticulate ER. Scale bar represents 10 *µ*m for all original images, and 5 *µ*m for all magnified inserts. Transient production of either mCherry-tagged ScWS or ScFAR showed expected subcellular localization, and normal ER organization. Co-production of either fluorescently tagged ScWS or ScFAR with untagged ScFAR or ScWS, respectively, showed no alteration to the cellular localization of the proteins, but did induce the same “swollen” BODIPY-stained regions, further supporting the concept that the ER defects are a result of mispackaging of WEs. **C)** Quantification of the total amount of WEs produced in *N. benthamiana* leaves by ESI-MS analysis. Values correspond to average of triplicate WE measurements (*n* = 3). Error bars correspond to standard deviation. Different letters indicate significant difference at *P* ≤ 0.05, as determined by Kruskal–Wallis test followed by Dunn's test. For 1 infiltration experiment, *A. tumefaciens* harboring a ScFAE was included to elongate endogenous *N. benthamiana* fatty acids to 20 and 22 carbon in length to mimic endogenous jojoba fatty acids. Only upon the co-production of the enzymes ScWS and ScFAR, was there an increase in the amount of WEs in *N. benthamiana* leaves. Additional expression of ScFAE showed a modest increase (although not statistically significant) in the amount of WEs synthesized. DW, dry weight; LD, lipid droplet.

To assess the capacity of jojoba LD proteins for packaging WEs into LDs, we reconstituted WE biosynthesis in *N. benthamiana* leaves via *Agrobacterium*-mediated ectopic-(co)expression of jojoba WS (ScWS) and jojoba FAR (ScFAR). As shown in Fig. 1A, expression of both ScWS and ScFAR in *N. benthamiana* leaves revealed large, diffuse-stained, swollen-like structures within the ER, which was labeled with the ER marker protein cyan fluorescent protein (CFP)-HDEL, (Szymanski et al. 2007) that co-localized with the neutral-lipid-specific dye BODIPY (493/503) (Qiu and Simon 2016). To confirm that the BODIPY staining of ER defects was not a result of CFP-tagged recombinant protein overexpression, ScWS and ScFAR were co-expressed without CFP-HDEL, and leaves were stained with BODIPY (Supplementary Fig. S1, bottom row). Similar large, diffuse structures were stained with BODIPY (arrows), indicating that the ER disruption most likely reflected neutral lipid accumulation (i.e. newly synthesized WE) that was trapped in the ER rather than being properly partitioned into LDs.

To confirm that these ER structures were due to aberrant WE accumulation, and not due to protein aggregation from ectopic overexpression of the ScWS and ScFAR enzymes, N-terminal mCherry-tagged ScWS and ScFAR were co-expressed either with CFP-HDEL only or with both CFP-HDEL and an untagged ScFAR or ScWS respectively. As shown in Fig. 1B, mCherry-ScWS expression with CFP-HDEL revealed that mCherry-ScWS localized in an expected manner to the ER and showed a normal reticulate-like ER pattern. Similarly, expression of both mCherry-ScWS and untagged ScFAR showed the same localization of mCherry-ScWS to the ER, and the same ER structures colocalizing with neutral lipid staining observed with ScWS and ScFAR co-expression (Fig. 1A), but no apparent aggregation of either protein at those ER defect sites (Fig. 1B). Expression of mCherry-ScFAR with CFP-HDEL also showed cytoplasmic localization of ScFAR and normal reticulate ER structure (Fig. 1B). On the other hand, when mCherry-ScFAR and CFP-HDEL were expressed with untagged ScWS, the mCherry-ScFAR showed cytoplasmic localization, but, importantly, ER defects were observed and there was a lack of mCherry-ScFAR localization to the swollen ER regions (Fig. 1B). As expected ScWS and mCherry-ScFAR expression caused the appearance of swollen BODIPY-stained structures (Fig. 1B, bottom row) similar to those seen when untagged ScWS and untagged ScFAR were expressed with CFP-HDEL (Fig. 1A). Expression of both ScWS and ScFAR also showed a noticeable increase in the number of LDs in addition to the appearance of ER defects, which may be the result of partial, but incomplete, partitioning of WEs into LDs (Fig. 1B). WEs also were quantified for each treatment to support our microscopic results that ER defects were a result of WE accumulation (Fig. 1C). Indeed, only upon the co-expression of ScWS and ScFAR was there a noticeable increase in WE accumulation and the appearance of disrupted, swollen ER regions. There was a slight but not significant increase in WE formation when the jojoba FAE (ScFAE) was included in transient assays (Fig. 1C), likely due to the increased availability of very long chain substrates for ScFAR and ScWS (see Supplementary Fig. S2), so ScFAE was included in all subsequent experiments to support optimal WE synthesis. Taken together these results suggested that swollen regions of ER in ScWS and ScFAR expressing cells were a result of accumulation and improper WE release from the ER. This confocal laser scanning microscopy (CLSM)-based assay provided a means to visually survey jojoba proteins for their capacity to promote the proper partitioning of WEs from the ER into LDs.

**Figure 2.**
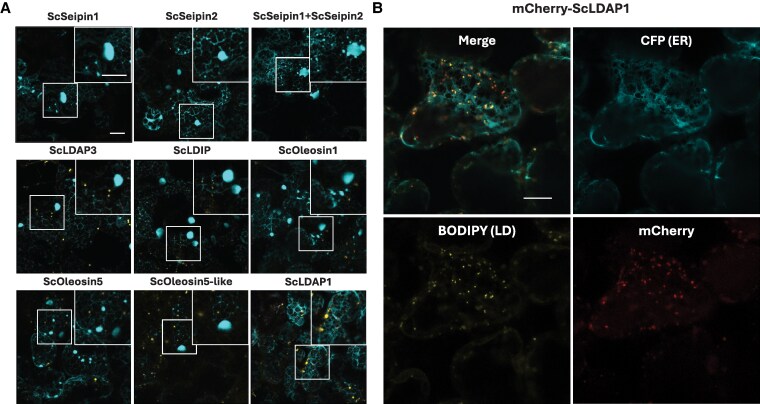
Transient expression of jojoba LD packaging proteins and their capacity to correct WE-induced ER defects. **A)** Representative CLSM images (z-sections) of infiltrated *N. benthamiana* leaves transiently producing ScWS, ScFAR, ScFAE, and a CFP-tagged ER marker (CFP-HDEL), in conjunction with jojoba LD proteins either individually, or in the case of jojoba Seipin1 (ScSeipin1) and jojoba Seipin2 (ScSeipin2), in combination with 1 another. All samples were stained with BODIPY (493/503) to visualize neutral lipids. Inset images highlight ER defects. Scale bars represent 20 *µ*m for both original images, and magnified inserts. BODIPY (493/503), yellow; CFP-HDEL, cyan. For each transient expression experiment *A. tumefaciens* harboring the P19 viral suppressor construct was added to suppress transgene silencing. All samples were stained and imaged (Zeiss LSM710 confocal laser scanning microscope, fitted with AIRYSCAN attachment) at 4 d post infiltration. Of the jojoba LD proteins produced with the WE synthesizing enzymes, only ScLDAP1 showed the capacity to restore ER defects suggesting it has some involvement in WE packaging. **B)** Representative high-resolution AIRYSCAN images of infiltrated *N. benthamiana* leaves transiently producing ScWS, ScFAR, ScFAE, CFP-HDEL, and mCherry-tagged ScLDAP1. Samples were stained with BODIPY (493/503) to visualize neutral lipids. ScLDAP1 production with WE synthesizing enzymes, showed “normal” reticulate ER organization, and BODIPY-stained LDs colocalizing with mCherry-tagged ScLDAP1. CFP-HDEL, cyan; BODIPY (493/503), yellow; mCherry, magenta. Scale bar represents 10 *µ*m for all images.

Previous proteomic and transcriptomic analyses of developing jojoba seeds identified several candidate LDAPs that were enriched in WE-accumulating seed tissues (Sturtevant et al. 2020). Of these, we selected the following jojoba proteins for expression in *N. benthamiana* leaf cells, along with co-expressed untagged ScWS and ScFAR: Seipin1, Seipin2, LDAP1, LDAP3, LDAP-interacting protein (LDIP), oleosin1, oleosin5, and oleosin5-like. As shown in Fig. 2A, the majority of the jojoba proteins examined showed no noticeable reduction in the swollen regions of the ER, with the exception of ScLDAP1. Specifically, ScLDAP1 co-expression with ScWS and ScFAR appeared to ameliorate the ER disruptions and promote normal LD accumulation. LDAP1 proteins were shown previously to localize to the LD monolayer, and in transient or stable plant expression assays, promote the accumulation of LDs (Gidda et al. 2013, 2016; Horn et al. 2013). Consistent with this, N-terminal mCherry-tagged ScLDAP1 (mCherry-LDAP1) co-expressed with untagged ScWS and ScFAR localized specifically to LDs (based on its colocalization with BODIPY; Fig. 2B), and this was accompanied by an obvious reduction in ER swelling, i.e. compared with cells only co-expressing ScWS and ScFAR (Fig. 1). Collectively, these results suggested that ScLDAP1 promoted the efficient partitioning of WEs from the ER into cytoplasmic LDs that are stabilized by the binding of ScLDAP1.

**Figure 3.**
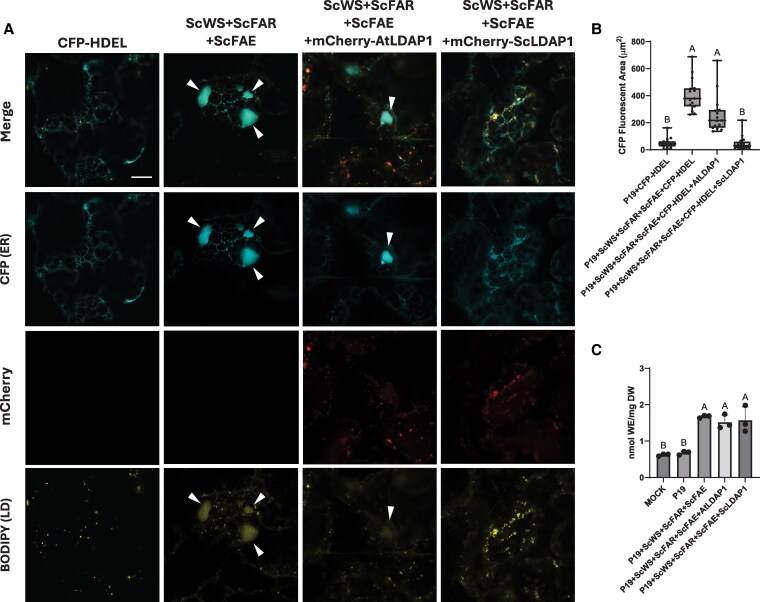
Comparison of ScLDAP1 and AtLDAP1s effect on ER structures and WE levels in *N. benthamiana* leaves. **A)** Representative confocal laser scanning microscopy (CLSM) images (z-sections) of infiltrated *N. benthamiana* leaves transiently producing ScWS, ScFAR, ScFAE, a CFP-tagged ER marker (CFP-HDEL), and either mCherry-tagged AtLDAP1, or mCherry-tagged ScLDAP1. Samples were stained with BODIPY (493/503) to visualize neutral lipids. Arrows point to regions of ER defects. CFP-HDEL, cyan; BODIPY (493/503), yellow; mCherry, magenta. Scale bar represents 10 *µ*m for all images. For each transient expression experiment, *A. tumefaciens* harboring the P19 viral suppressor construct was added to suppress transgene silencing. ScFAE was included to elongate endogenous *N. benthamaina* fatty acids to 20 and 22 carbon in length to mimic endogenous jojoba fatty acids. All samples were stained and imaged (Zeiss LSM710 confocal laser scanning microscope, fitted with AIRYSCAN attachment) at 4 d post infiltration. No mCherry fluorescence was detected in the absence of LDAP-mCherry-tagged proteins (third row, first 2 columns). Transient co-production of ScLDAP1 with WE synthesizing enzymes showed a restored reticulate ER structure, while co-production of AtLDAP1 showed no restoration, suggesting a species-specific WE specificity of ScLDAP1. **B)** Quantification of the CFP-fluorescent area (*µ*m^2^) associated with swollen ER structures. “Wax Synthesis” samples include the expression of P19 (endogenous silencing suppressor), CFP-HDEL, ScWS, ScFAR, and ScFAE collectively and in combination with either AtLDAP1, or ScLDAP1. Values correspond to the averages of 3 individual infiltration experiments (with 5 images from each replicate). Different letters indicate significant difference at *P* ≤ 0.05, as determined by Kruskal–Wallis test followed by Dunn's test. Whiskers of the box and whisker plot correspond to the upper 25% and lower 25% of values from the dataset. The upper lines and lower lines of the whiskers correspond to the maximum and minimum values of the dataset. The box corresponds to the middle 50% of the data with the line bisecting the box corresponding to the median of the data set. The top and bottom lines of the box correspond to the upper and lower quartile, respectively. Co-production of ScLDAP1 with WE synthesizing enzymes showed a significant reduction in CFP fluorescence area associated with WE-induced ER defects, while AtLDAP1 co-production did not. **C)** Quantification of the total amount of WEs produced in *N. benthamiana* leaves by ESI-MS analysis. “Wax Synthesis” samples include the expression of P19 (endogenous silencing suppressor), ScWS, ScFAR, and ScFAE collectively and in combination with either AtLDAP1, or ScLDAP1. Values correspond to triplicate extractions from leaves of 3 separate infiltrated plants. Different letters indicate significant difference at *P* ≤ 0.05, as determined by Kruskal–Wallis test followed by Dunn's test. Error bars correspond to standard deviation. Co-production of either ScLDAP1 or AtLDAP1 with WE synthesizing enzymes did not improve the accumulation of WE overproduction of just the wax synthesis enzymes. DW, dry weight; LD, lipid droplet.

LDAP1 is a ubiquitous LD protein found in all plants and is predominately expressed in vegetative tissues, such as leaves and stems (Gidda et al. 2016; Guzha et al. 2023). However, in jojoba, LDAP1 is most abundantly expressed in developing seeds during WE accumulation and is enriched in the proteome of isolated LDs (Sturtevant et al. 2020). To assess whether the reversal of the aberrant swollen ER phenotype in *N. benthamiana* leaves was specific to the LDAP1 from jojoba or rather was a property shared by LDAP1 isoforms from other plant species, N-terminal mCherry-tagged Arabidopsis LDAP1 (mCherry-AtLDAP1) was co-expressed with untagged ScWS and ScFAR. ER organization was then analyzed by CLSM based on the co-expressed ER marker protein CFP-HDEL and compared with jojoba mCherry-LDAP1. As shown in Fig. 3A, mCherry-AtLDAP1 localized to LDs similar to mCherry-ScLDAP1 but did not reverse the ER swelling or the release of the trapped neutral lipids. For a quantitative estimate of the restoration of the ER, the total fluorescent area of CFP-HDEL associated with the enlarged ER structures was measured and compared with control samples (i.e. empty vector [mock] and P19 viral suppressor of transgene silencing alone), or with the ScWS and ScFAR in the presence of either mCherry-ScLDAP1 or mCherry-AtLDAP1 (Fig. 3B). As anticipated from the visual CLSM results, mCherry-AtLDAP1 did not show a significant reduction in ER swelling area when compared with samples (co)expressing only ScWS and ScFAR, while mCherry-ScLDAP1 reduced the ER swelling to levels similar to leaves without WE production (CFP-HDEL alone; Fig. 3B). Similar ER swelling also was observed in cells overexpressing the native *N. benthamiana* LDAP1 (NbLDAP1), NbLDAP2 or NbLDAP3, as well as AtLDAP2 or AtLDAP3 (Supplementary Figs. S3 and S4), reinforcing the notion that only ScLDAP1 was able to promote efficient WE partitioning from the ER to LDs. That is, it appeared that the functional activity of WE partitioning from the ER was unique to ScLDAP1 and not a characteristic shared with broader LDAP isoform family members.

To compare the total WE levels accumulated by co-expressing either AtLDAP1 or ScLDAP1, total neutral lipids were extracted and WE levels were quantified by direct-infusion ESI-MS/MS (Fig. 3C). *N. benthamiana* leaves were infiltrated with ScWS, ScFAR, and ScFAE (to support fatty acid elongation for WE synthesis to better mimic WE substrates produced in jojoba [Supplementary Fig. S2]), with or without either AtLDAP1 or ScLDAP1. Overall, there was no difference in total WE content among any samples expressing the WE biosynthetic enzymes, regardless of whether an LDAP1 was co-expressed (Fig. 3C), suggesting that ScLDAP1 does not function to facilitate additional WE synthesis, but rather to promote more efficient partitioning of the synthesized WEs out of the ER and into LDs in these transient assays.

**Figure 4.**
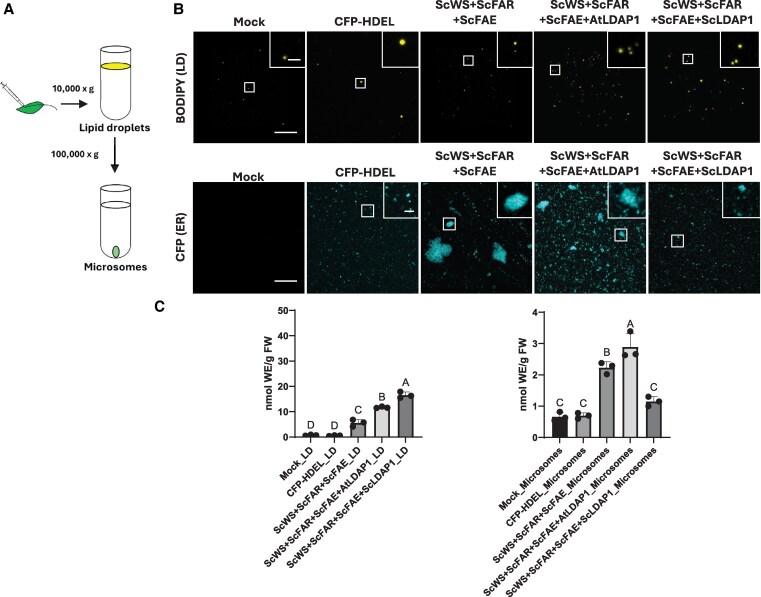
Analysis of WE distribution between isolated LDs and microsomes from infiltrated *N. benthamiana* leaves. **A)** Cartoon representation of the workflow for LD and microsome isolation from *N. benthamiana* leaves. **B)** Representative confocal laser scanning microscopy (CLSM) images (single plane) of LDs and microsomes isolated from infiltrated *N. benthamiana* leaves producing ScWS, ScFAR, ScFAE, a CFP-tagged ER marker (CFP-HDEL), and either AtLDAP1 or ScLDAP1. All isolated LD fractions were stained with BODIPY (493/503) to visualize neutral lipids. Magnified image inserts highlight both LDs and microsomes. Scale bars represent 40 *µ*m for each of the original images, and 5 *µ*m for each of the magnified inserts. CFP-HDEL, cyan; BODIPY (493/503), yellow. For each transient expression experiment *A. tumefaciens* harboring the P19 viral suppressor construct was added to suppress transgene silencing. All samples were collected at 4 d post infiltration. Addition of either AtLDAP1 or ScLDAP1 showed an increase in the number of LDs, but only ScLDAP1 showed a substantial decrease in the size of large ER-derived structures in microsomal fractions. **C)** Quantification of WEs from either LD or microsomal fractions isolated from infiltrated *N. benthamiana* leaves. “Wax Synthesis” samples include the expression of P19 (endogenous silencing suppressor), ScWS, ScFAR, and ScFAE collectively and in combination with either AtLDAP1, or ScLDAP1. Different letters indicate significant differences at *P* ≤ 0.05, as determined by 1-way ANOVA with Tukey's post-test (*n* = 3). Error bars correspond to standard deviation. Quantification of WEs in isolated LD fractions showed significant enrichment of WEs in samples co-producing ScLDAP1 with wax synthesis enzymes, compared with samples producing wax synthesis enzymes alone or with AtLDAP1. FW, fresh weight.

To corroborate in situ microscopy studies, a cell fractionation approach also was implemented to assess WE partitioning, whereby LDs from *N. benthamiana* leaf tissues were isolated by floatation centrifugation, and microsomes were pelleted from the remaining supernatant by ultracentrifugation (Fig. 4A). *N. benthamiana* leaves were infiltrated with ScWS, ScFAR, and ScFAE, with or without either AtLDAP1 or ScLDAP1. Then, LDs (marked by BODIPY) and microsomes (marked by CFP-HDEL) were isolated 4 d post infiltration. Representative images of each of these fractions are shown in Fig. 4B. LDs were more abundant in LD fractions that had harbored either of the LDAP1s compared with mock or CFP-HDEL controls (Fig. 4B). It has been previously shown that overexpression of AtLDAP1 increased the number of LDs accumulated in transgenic Arabidopsis leaves (Gidda et al. 2016), so it was not entirely unexpected that LDs would be induced/stabilized by either LDAP1 isoform. When comparing the microsomal fractions, a difference in the size and shape of CFP-HDEL-labeled ER-derived microsomes was obvious. That is, in samples producing the WE biosynthesis enzymes alone, large CFP-HDEL-containing structures were evident (Fig. 4B) similar in size to the swollen ER regions visualized in leaves (Figs. 1A, 2A, and 3A). Co-expression of AtLDAP1 yielded smaller sized CFP-HDEL structures, whereas the co-expression of ScLDAP1 resulted in the relative absence of these large structures from the microsomal fractions (Fig. 4B). Neutral lipids were quantified in each of these subcellular fractions and the amounts are reported in Fig. 4C and Supplementary Fig. S5. Overall, more WEs (on a tissue weight basis) were recovered in the LD fractions isolated from leaves expressing ScLDAP1, compared with fractions with AtLDAP1 or the WE biosynthesis genes alone (Fig. 4C). However, the relative proportion of WEs remaining in the microsomes appeared to be greater in the absence of ScLDAP1 (Fig. 4C). That is, the coincident production of ScLDAP1 resulted in the most WEs recovered in the LDs, and the samples with AtLDAP1 appeared to recover significantly less WEs in LDs (Fig. 4C). Interestingly, the quantification of TAGs showed the reverse, whereby there was significantly more TAG in the LD fraction in the presence of AtLDAP1 compared with ScLDAP1 (or in the absence of either any LDAP1) (Supplementary Fig. S5). Despite the caveats of losses of material during cell fractionation, it appears from these biochemical studies that the partitioning of WEs into LDs is promoted by ScLDAP1 greater than AtLDAP1. It is tempting to speculate from these results that ScLDAP1 has evolved to be more selective for the packaging of WEs and that AtLDAP1 is more selective for TAGs.

### The function of ScLDAP1 in WE partitioning depends on LDIP

**Figure 5.**
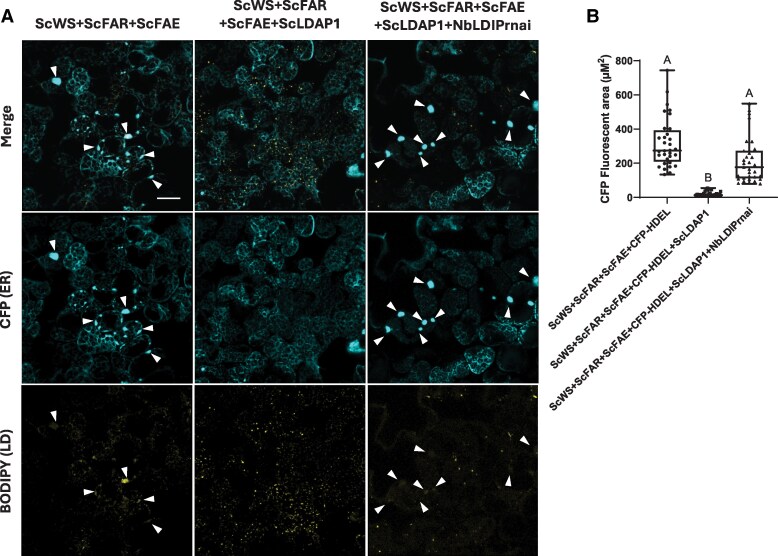
RNAi suppression of NbLDIP shows a loss of ScLDAP1 WE-specific function. **A)** Representative confocal laser scanning microscopy (CLSM) images (z-sections) of *N. benthamiana* transiently producing ScWS, ScFAR, ScFAE, a CFP-tagged ER marker (CFP-HDEL), and either ScLDAP1, or ScLDAP1 and an NbLDIP RNAi suppressor (NbLDIPrnai). Samples were stained with BODIPY (493/503) to visualize neutral lipids. Arrows point to regions of ER defects. Scale bar represents 40 *µ*m and is the same for all images. CFP-HDEL, cyan; BODIPY (493/503), yellow. For each transient expression experiment, *A. tumefaciens* harboring the P19 viral suppressor construct was added to suppress transgene silencing. All samples were stained and imaged (Zeiss LSM710 confocal laser scanning microscope) at 4 d post infiltration. Confirmation of RNAi suppression of NbLDIP was evaluated by qualitative RT-PCR (Supplementary Fig. S6). RNAi suppression of NbLDIP caused a reversion of ScLDAP1-induced “normal” ER organization back to swollen ER defects. This suggests that LDIPs may be required for ScLDAP1 to perform its WE-specific function. **B)** Quantification of the CFP-fluorescent area (*µ*m^2^) associated with swollen ER defects. “Wax Synthesis” samples include the expression of P19 (endogenous silencing suppressor), CFP-HDEL, ScWS, ScFAR, and ScFAE collectively and in combination with either ScLDAP1, or ScLDAP1 and NbLDIPrnai. CFP quantification was based on a minimum of 29 images from 3 replicate infiltration experiments for each treatment. Different letters indicate significant differences in comparison between each sample as determined by Kruskal–Wallis test followed by Dunn's test. (*P* ≤ 0.05). Whiskers of the box and whisker plot correspond to the upper 25% and lower 25% of values from the dataset. The upper lines and lower lines of the whiskers correspond to the maximum and minimum values of the dataset. The box corresponds to the middle 50% of the data with the line bisecting the box corresponding to the median of the data set. The top and bottom lines of the box correspond to the upper and lower quartiles respectively. Quantification of the CFP-fluorescent area associated with WE-induced ER defects shows a significant reduction in the fluorescent area associated with these defects with ScLDAP1, and this reduction is reversed by suppression of endogenous NbLDIP. LD, lipid droplet.

A previous yeast 2-hybrid interaction screen identified an Arabidopsis hydrophobic protein that directly interacts with LDAP proteins (Pyc et al. 2017a). This LDIP, was subsequently shown to interact with not only LDAPs but also SEIPIN proteins in Arabidopsis and was essential for the proper formation of LDs (Pyc et al. 2021). We hypothesized that endogenous *N. benthamiana* LDIP (NbLDIP) might participate in the partitioning function of ScLDAP1. To test this possibility, we utilized an RNA interference (RNAi) assay previously used to suppress NbLDIP expression in leaves (Pyc et al. 2021) to examine the effects of LDIP suppression in the WE reconstitution assays. As shown in Fig. 5A, LDIP suppression (confirmed by RT-PCR, Supplementary Fig. S6) in leaves that were co-expressing WE biosynthetic enzymes and ScLDAP1 reduced the corrective effect to the ER afforded by ScLDAP1. The ScLDAP1 function was confirmed to be LDIP-dependent based on CFP-fluorescent area quantification of ER defects (Fig. 5B). We conclude that ScLDAP1 capacity to more efficiently partition WEs into LDs was dependent on sufficient levels of endogenous LDIP, suggesting that WE-containing LDs formation may proceed in an analogous manner to that of TAG- containing LDs except that ScLDAP1 has evolved features that make it specific to WEs.

### Both ScLDAP1 and AtLDAP1 preferentially associate with WE-filled monolayers in MDS

The association of LDAP with LDs was explored previously, and the results were somewhat enigmatic (Gidda et al. 2016). Any truncations of the protein resulted in its mis-localization from the LD surface to the cytoplasm in vivo, and liposome-binding experiments in vitro did not really confirm differential affinity for a specific lipid composition. Consequently, it was not clear what part of the LDAP1 protein is involved in its localization to LDs specifically. LD proteins in general preferentially associate with LD monolayers and this has been proposed to involve several factors, including hydrophobicity, membrane charge, and packing defects that occur at the phospholipid monolayer overlying the neutral lipids during LD formation (Prévost et al. 2018; Chorlay and Thiam 2020; Dhiman et al. 2020).

To gain some insights into the interactions of ScLDAP1 and AtLDAP1 with a phospholipid surface, protein structural models were generated using AlphaFold2 (Jumper et al. 2021), and these were utilized in MDS to assess protein binding preferences to both a simulated ER bilayer and a simulated WE-filled monolayer (WE core with an overlying phospholipid monolayer to replicate a LD). These membrane surfaces were simulated using published lipid class and molecular species compositions quantified from *Nicotiana tabacum* leaves (Ruzicska et al. 1983; Ishida 2013), or jojoba seed oil (Gülz and Eich 1982; Sturtevant et al. 2020; Supplementary Table S1, Figs. S7 to S10, Videos S1 and S2). To simulate ScLDAP1 and AtLDAP1 interactions with the membrane surface in an unbiased manner, both proteins were placed in 6 different orientations 50 Å above the phospholipid surface. The 6 orientations were selected by rotating LDAP1 as though it were a cube, in 90-degree increments such that each face of the cube was proximal to the membrane. As we do not know in advance which residues should bind to the membrane, this eliminates our own biases in the simulation setup. From this starting configuration, LDAP1 and the membrane were simulated for 1,000 ns to capture association interactions. In previous studies, 1,000 ns has been an ample time frame to arrive at a consistent bound pose (Vermaas and Tajkhorshid 2017; Kulke et al. 2024). These simulations are organized into interactions with a bilayer model, a wax-ester-filled monolayer, and a TAG-filled monolayer (Supplementary Videos S3 to S76).

**Figure 6.**
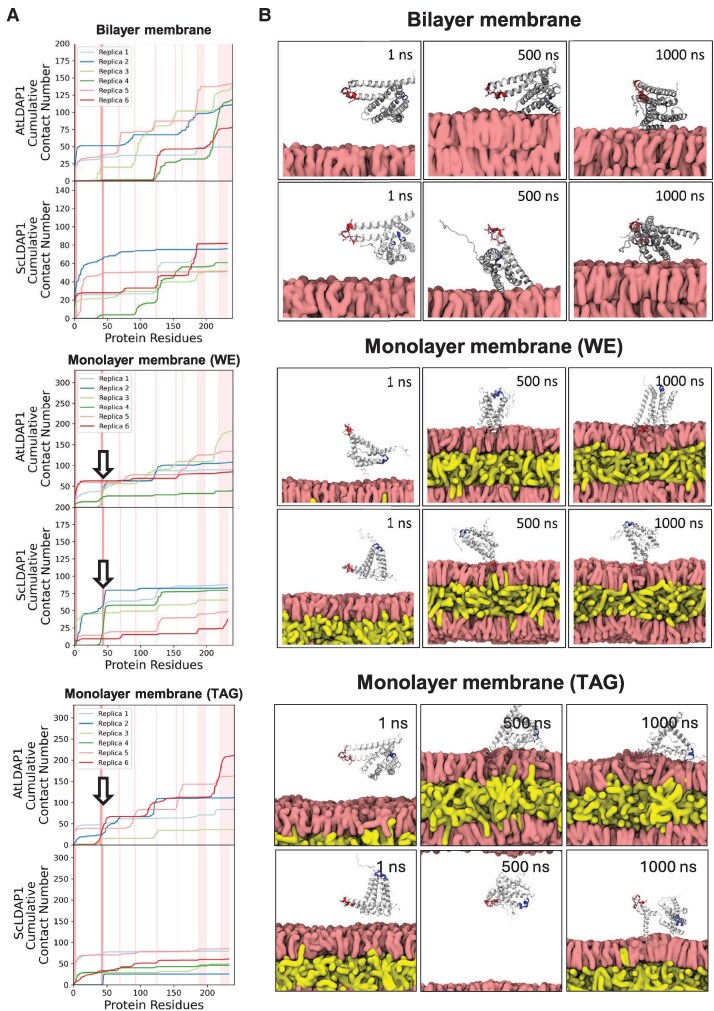
Simulated interactions of AtLDAP1 and ScLDAP1 with a jojoba-like bilayer and a WE-filled and a TAG-filled monolayer. **A)** Average contact number per LDAP1 protein residue to jojoba-like membrane bilayer, and WE- and TAG-filled monolayer, reported as a cumulative sum. Average contact number was calculated based on the bound conformation where the contact number of each residue to the membrane was >25. Different colored lines represent a different simulation trajectory. White arrows denote the region that makes marked contact with the surface. This region is colored dark red in the snapshots shown in **B)**. Light red regions correspond to the loops and turns found in the secondary structure of LDAP1. See simulations, Supplementary Videos S3 to S8, MDS of AtLDAP1 with a jojoba-like bilayer; Supplementary Videos S9 to S14, MDS of ScLDAP1 with a jojoba-like bilayer; Supplementary Videos S15 to S20, MDS of AtLDAP1 with a jojoba-like WE-filled monolayer; Supplementary Videos S21 to S26, MDS of ScLDAP1 with a jojoba-like WE-filled monolayer; Supplementary Videos S53 to S58, MDS of AtLDAP1 with a jojoba-like TAG-filled LD membrane; Supplementary Videos S59 to S64, MDS of ScLDAP1 with a jojoba-like TAG-filled LD membrane. **B)** Representative snapshots at 1, 500, and 1,000 ns, of LDAP1s simulated with the bilayer, and WE- and TAG-filled monolayers. The red highlighted region in snapshots corresponds to the loop identified to directly insert into the monolayer. Blue region is the amphipathic α-helix identified through mutation analysis. See simulations, Supplementary Videos S3 to S8, MDS of AtLDAP1 with a jojoba-like bilayer; Supplementary Videos S9 to S14, MDS of ScLDAP1 with a jojoba-like bilayer; Supplementary Videos S15 to S20, MDS of AtLDAP1 with a jojoba-like WE-filled monolayer; Supplementary Videos S21 to S26, MDS of ScLDAP1 with a jojoba-like WE-filled monolayer; Supplementary Videos S53 to S58, MDS of AtLDAP1 with a jojoba-like TAG-filled LD membrane; Supplementary Videos S59 to S64, MDS of ScLDAP1 with a jojoba-like TAG-filled LD membrane.

LDAP1 showed binding with WE-filled monolayer, while the bilayer simulation showed no binding of LDAP1 with membrane. To determine if the bound poses would coalesce into a single bound pose, we extended LDAP1/WE simulations to 2,000 ns. The bound poses remained distinct, and so we also chose 1,000 ns for the remaining simulations when binding to TAG membranes. Figure 6A shows traces for each of 6 replicates for the cumulative contacts of either the AtLDAP1 protein or the ScLDAP1 protein interacting with a jojoba-like bilayer (top) or a monolayer filled with either WEs (middle) or TAGs (bottom). Figure 6B shows representative simulation snapshots at 1, 500, and 1,000 ns. Since each simulation began with a different orientation of the LDAP1 protein above the membrane surface, we anticipated that consistent binding interactions between the protein and membrane surface would reflect the observed orientation that would also be preferred in vivo.

An important initial question to address was if LDAP1 would interact with the membrane surface during our relatively short simulations. In most cases, protein-membrane contacts were observed before the end of the simulation, although some initial poses exhibited faster binding than others (Fig. 6A, Supplementary Figs. S11 and S12). Other poses were seen to bind reversibly during simulation, with the contact number going back to zero after an initial association (Supplementary Fig. S11). A general trend when comparing the simulated interactions with the bilayer membrane and the WE-filled bilayer (Fig. 6A, Supplementary Figs. S11 and S12) was that both the ScLDAP1 or AtLDAP1 protein made more contacts to lipophilic components if WEs were present under the phospholipid monolayer surface.

In simulation trajectories featuring either WE-filled, or TAG-filled monolayers without proteins, the WEs and TAGs were observed to occasionally come to the surface of the membrane and were solvent-exposed (Supplementary Fig. S7, Videos S1, S2, S51 and S52), representing what others have described as packing defects that may be recognized by LD proteins. We quantified the defect area through the PackMem software package (Gautier et al. 2018), which indicated larger defects for TAG-filled membrane monolayers than WE-filled monolayers (Supplementary Fig. S13). Those exposed WEs or TAGs potentially could allow the LDAP1 proteins deeper penetration into the monolayer and thus provide a means for the protein to recognize the LD surface versus the bilayer membrane.

Contrary to what might have been expected based on the WE selectivity of ScLDAP1 demonstrated in cell biology assays (Figs. 2 to 5), AtLDAP1 made more membrane surface contacts in general when compared with ScLDAP1 in these simulations (Fig. 6A). However, AtLDAP1 often contacted the membrane with structured regions lying flat against the membrane surface, whereas the membrane contacts of ScLDAP1 were mainly at protein turns and coils (Fig. 6, A and B), with a particular emphasis toward the N-terminus—compare, for example, Supplementary Video S15 (AtLDAP1) with Supplementary Video S21 (ScLDAP1). Ultimately, the same region of both AtLDAP1 and ScLDAP1 seemed to consistently drive the molecular interactions with the monolayer, especially for WE, and is depicted by a red-colored region within the polypeptide backbone in Fig. 6B. Looking at the cumulative contact plot for the monolayer and WE models, there was a specific region across residues 40 to 50 that had increased cumulative contacts in AtLDAP1 and interactions were especially pronounced for ScLDAP1 (Fig. 6A, arrows in middle 2 plots). The region colored in red that appeared to interact well with the WE-filled monolayer did not show pronounced interactions with the membrane bilayer for either protein during the simulations (Fig. 6, A and B; top 2 rows).

**Figure 7.**
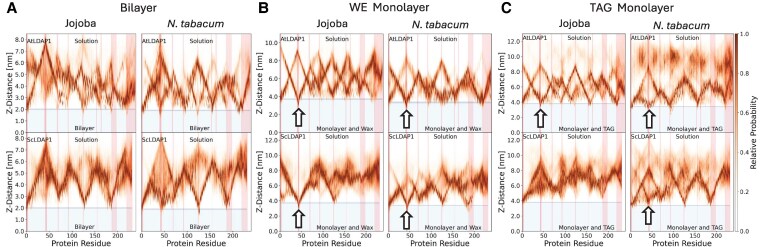
Probability distribution of depth of LDAP1 residues interacting with a jojoba or *N. tabacum* membrane bilayer, WE-filled monolayer, or TAG-filled monolayer. **A)** Depth plots illustrate the probability of insertion depth of either ScLDAP1 or AtLDAP1 residues in a jojoba- or *N. tabacum-*like bilayer membrane system. Depth probability distribution is calculated from the relative position of each residue from the center of the bilayer. Relative probability is mathematically summarized as *P_i_*(*z*)/*P_i_*(max), with *P_i_*(*z*) corresponding to the probability of a residue (*P_i_*) at a given height (*z*) from the center of the bilayer, and *P_i_*(max) corresponding to the maximum probability observed for each residue (*P_i_*). Plot color refers to the residue-by-residue probability distribution of how far that specific residue is from the membrane center. Residues plotted as Z-distance from the membrane center (set as 0 nm). Light blue portion of the graph corresponds to the membrane bilayer, with the top of the light blue area corresponding to the approximate positioning of phospholipid headgroups. Light red regions correspond to the loop and turn found in the secondary structure. **B)** Depth plots illustrate the probability of insertion depth of either ScLDAP1 or AtLDAP1 residues in a jojoba-like WE-filled membrane system, or an *N. tabacum* phospholipid membrane system, filled with *N. benthamiana-*like WEs. Depth probability distribution is calculated from the relative position of each residue from the center of the WE-filled membrane. Relative probability is mathematically summarized as *P_i_*(*z*)/*P_i_*(max), with *P_i_*(*z*) corresponding to the probability of a residue (*P_i_*) at a given height (*z*) from the center of the bilayer, and *P_i_*(max) corresponding to the maximum probability observed for each residue (*P_i_*). Residues plotted as Z-distance from the membrane center (set as 0 nm). Plot color refers to the residue-by-residue probability distribution of how far that specific residue is from the membrane center. The light blue portion of the graph corresponds to the membrane bilayer, with the top of the light blue area corresponding to the approximate positioning of phospholipid headgroups. Light red regions correspond to the loop and turn found in the secondary structure. **C)** Depth plots illustrate the probability of insertion depth of either ScLDAP1 or AtLDAP1 residues in a jojoba-like, or *N. tabacum*-like TAG-filled membrane system. Depth probability distribution is calculated from the relative position of each residue from the center of the TAG-filled membrane. Relative probability is mathematically summarized as *P_i_*(*z*)/*P_i_*(max), with *P_i_*(*z*) corresponding to the probability of a residue (*P_i_*) at a given height (*z*) from the center of the bilayer, and *P_i_*(max) corresponding to the maximum probability observed for each residue (*P_i_*). Residues plotted as Z-distance from the membrane center (set as 0 nm). Plot color refers to the residue-by-residue probability distribution of how far that specific residue is from the membrane center. Light blue portion of the graph corresponds to membrane bilayer, with the top of the light blue area corresponding to the approximate positioning of phospholipid headgroups. Light red regions correspond to the loop and turn found in the secondary structure. Data for A, B, and C extracted from multiple simulations—Supplementary Videos S3 to S8, MDS of AtLDAP1 with a jojoba-like bilayer; Supplementary Videos S9 to S14, MDS of ScLDAP1 with a jojoba-like bilayer; Supplementary Videos S15 to S20, MDS of AtLDAP1 with a jojoba-like WE-filled monolayer; Supplementary Videos S21 to S26, MDS of ScLDAP1 with a jojoba-like WE-filled monolayer; Supplementary Videos S27 to S32, MDS of AtLDAP1 with a *N. tabacum-* like bilayer; Supplementary Videos S33 to S38, MDS of ScLDAP1 with a *N. tabacum-* like bilayer; Supplementary Videos S39 to S44, MDS of AtLDAP1 with a *N. tabacum-* like phospholipid monolayer, filled with *N. benthamiana-* like WEs; Supplementary Videos S45 to S50, MDS of ScLDAP1 with a *N. tabacum-*like phospholipid monolayer, filled with *N. benthamiana-*like WEs; Supplementary Videos S53 to S58, MDS of AtLDAP1 with a jojoba- like TAG-filled monolayer; Supplementary Videos S59 to S64, MDS of ScLDAP1 with a jojoba- like TAG-filled LD membrane; Supplementary Videos S65 to S70, MDS of AtLDAP1 with a *N. tabacum-* like TAG-filled monolayer; Supplementary Videos S71 to S76, MDS of ScLDAP1 with a *N. tabacum-* like TAG-filled monolayer.

ScLDAP1 and AtLDAP1 both were shown to interact with the WE-filled membrane through a contact metric (Fig. 6); however, penetration depth provides a complementary and perhaps more intuitive view of which residues were most important for the membrane interaction (Fig. 7). From the depth plots, which express the penetration depth in probabilistic terms based on the 6 trajectories for each membrane model, there were multiple dips where a residue came in close proximity to the membrane, or even is inserted into it (Fig. 7). For membrane bilayers without WEs, the extreme N-terminus is the region that inserts most consistently into the membrane (Fig. 7). By contrast, there is a region just before amino acid 50 where both proteins interact with the monolayer membrane filled with WEs and is especially strong for several simulation trajectories for the ScLDAP1. For AtLDAP1 this region corresponded to a disordered loop at residues 40 to 43 that seemed to insert more consistently into the WE-filled membrane, and for ScLDAP1, this same loop, spanning residues 42 to 44, also was more probable to interact with the WE-filled membrane (Fig. 7). These regions are colored red for both proteins in the simulation snapshots presented in Fig. 6 (and all Supplementary Videos S3 to S50 and S53 to S76). Most notably, this protein region remained the furthest distance from the bilayer surface for all simulations for both proteins (Fig. 7, left 2 plots). Given these probability trends, both LDAP1 proteins appear to interact with the WE-filled monolayer better (compared with the bilayer surface) through this same loop region, it is possible that this region may be important as a part of a targeting signal for localizing the LDAPs to the LD monolayer surface in general, rather than being a selective feature of the ScLDAP1 for WEs. The probability distribution also suggests that ScLDAP1 has more specificity for binding with WE-filled monolayers as the AtLDAP1 distribution was spread over a larger distance in comparison to ScLDAP1 (Fig. 7). ScLDAP1 disorder loop region can insert into the jojoba membrane independent of the initial face of the protein, there seems to be enough dynamics to rotate the ScLDAP1 protein and it finds the preferred orientation with membrane to insert the disorder loop in the membrane (Fig. 7, Supplementary Videos S31 to S36).

### AtLDAP1 has a somewhat higher association rate with TAG-filled monolayers in MDS

The molecular simulations to this point established a preferential association of LDAP1 with WE-filled monolayer compared with a normal lipid bilayer (Figs. 6 and 7). While jojoba primarily makes WEs, TAGs similarly are packaged into LDs in most other oilseeds (Birsoy et al. 2013; de Vries and Ischebeck 2020; Guzha et al. 2023), and so comparing WE-filled- with similar TAG-filled monolayer models was a natural point of comparison. Using the same AlphaFold models and simulation conditions as for the WEs enabled a direct comparison. Given the LDAP1 protein interactions we had observed with WE (Figs. 6 and 7; bottom 2 rows), we were expecting to see that the LDAP1 proteins would interact with the hydrophobic core of TAG-filled monolayermodel membranes. However, the contact maps (Fig. 6A) of LDAP1 proteins showed a generally lower number of contact numbers, when compared with the WE-filled monolayers (plots on bottom row). The AtLDAP1 motif around residues 40 to 50 showed higher interaction with the TAG-filled LD when compared with ScLDAP1, but was still lower overall when compared with its interaction with the WE-monolayers (Fig. 6A; bottom arrow, compare with middle rows; see also Supplementary Video S58). Only 1 of the 12 replicas of ScLDAP1 had the same interaction between the membrane surface and the domain around residue 40 to 50 (Fig. 6A, bottom row). For many other replicas of ScLDAP1 binding to a TAG-filled membrane, the simulation phenotype was similar to that of the bilayer, where ScLDAP1 either laid flat on the surface of the membrane or bounced off the membrane surface. This suggests that the interaction of ScLDAP1 with TAG-filled monolayers might be less efficient compared with its interaction with WE-filled monolayers and that AtLDAP1 might be better than ScLDAP1 at recognizing the TAG-filled monolayer surface.

Deeper penetration of AtLDAP1 and ScLDAP1 proteins in the TAG-filled monolayer was quantified similarly by calculating the probability distribution of LDAP1 residues along the membrane-normal axis relative to the LD center (Fig. 7). The same disordered loop of AtLDAP1 (amino acids 40 to 43) was responsible for penetration into the LD (see Supplementary Video S70 for example). ScLDAP1 showed much less penetration in LD compared with AtLDAP1 (Fig. 7). Although this disordered loop may anchor LDAP1 protein to the LD, it also showed some preference for binding more to a WE-filled monolayer compared with the TAG-filled monolayer. We opted not to extend the simulation for another 1,000 ns as we did not observe any specific binding pose except the insertion of the disordered loop into the TAG-filled monolayer.

It is important to point out here that the area of membrane defects in TAG-filled LD models was higher compared with WE-filled monolayers (Supplementary Fig. S13). Compare also the surface defect dynamics in Supplementary Videos for WE-filled monolayers versus TAG-filled monolayers (Supplementary Videos S1 and S2 versus S51 and S52). Therefore, the specificity of ScLDAP1 for WE-filled monolayers could be attributed to the defect size in the monolayers, but the exact mechanism for this specificity is not immediately clear. However, if we assume that ScLDAP1 preferentially binds to smaller rather than larger defects, such a hypothesis would be consistent with only observing 1 of the replicas of the ScLDAP1 to bind extensively with a TAG-filled monolayer (see Supplementary Video S76). Perhaps other replicas just did not encounter a suitable binding patch. This searching process is highly stochastic and is not just dependent on the proteins being in the right orientation, but also that the protein lands on a suitable membrane patch. We speculate that our choice for a planar model to reduce computation cost may reduce the propensity of membrane defects compared with a curved membrane that would exist in a LD.

### ScLDAP1 requires a specific amphipathic α-helix for its WE specificity

**Figure 8.**
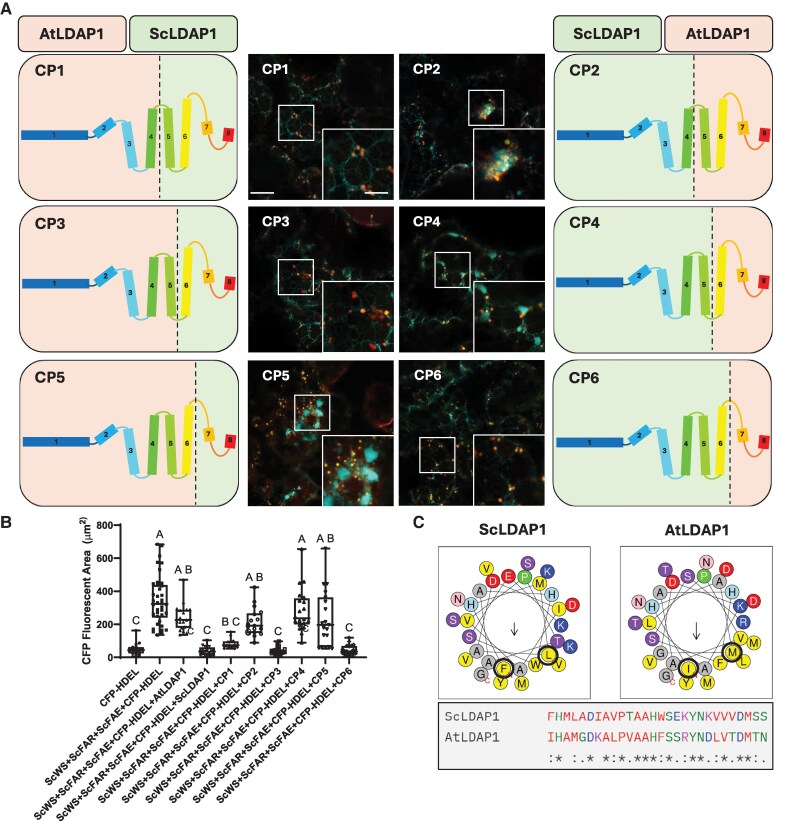
Chimeric LDAP1 expression shows restoration of ER organization and LD packaging. **A)** Representative high-resolution AIRYSCAN images of infiltrated *N. benthamiana* leaves transiently expressing ScWS, ScFAR, ScFAE, a CFP-tagged ER marker (CFP-HDEL), and N-terminal mCherry-tagged chimeric LDAP1s. Samples were stained with BODIPY (493/503) to visualize neutral lipids. Higher magnification inserts highlight regions of normal ER and WE-induced ER defects. Scale bars represent 10 *µ*m for all original images, and 5 *µ*m for all magnified inserts. CFP-HDEL, cyan; BODIPY (493/503), yellow; mCherry, magenta. For each transient expression experiment, *A. tumefaciens* harboring the P19 viral suppressor construct was added to suppress transgene silencing. All samples were stained and imaged (Zeiss LSM710 confocal laser scanning microscope, fitted with AIRYSCAN attachment) at 4 d post infiltration. Cartoon diagrams illustrate regions of chimeric swaps between AtLDAP1 and ScLDAP1 indicated by a dotted line. Cartoons representing secondary structures (rectangles, α helices; lines, disordered loops) are colored in a rainbow scheme starting from the N-terminus to C-terminus of the protein. Based on the expression of each CP, 1 helix from ScLDAP1 (colored yellow, helix 6) appeared to be essential to correct WE-induced ER defects. **B)** Quantification of the CFP-fluorescent area (*µ*m^2^) associated with swollen ER defects. “Wax Synthesis” samples include the expression of P19 (endogenous silencing suppressor), CFP-HDEL, ScWS, ScFAR, and ScFAE collectively and with AtLDAP1, ScLDAP1, CP1, CP2, CP3, CP4, CP5, or CP6. Values correspond to the averages of images taken from 3 separate infiltration experiments (with ∼7 images from each replicate). Different letters indicate significant difference at *P* ≤ 0.05, as determined by Kruskal–Wallis test followed by Dunn's test. Whiskers of the box and whisker plot correspond to the upper 25% and lower 25% of values from the dataset. The upper lines and lower lines of the whiskers correspond to the maximum and minimum values of the dataset. The box corresponds to the middle 50% of the data with the line bisecting the box corresponding to the median of the data set. The top and bottom lines of the box correspond to the upper and lower quartiles, respectively. **C)** Helical wheel projection of the essential α helices from both AtLDAP1 and ScLDAP1 (generated by Heliquest webserver). Hydrophobic residues are colored yellow, hydrophilic and charged residues are colored purple, blue, pink, red, and blue. Alanine and glycine residues are colored gray. Proline residues are colored green. The direction of the arrowhead in the center of the wheel indicates the position of the hydrophobic face along the axis of the helices. Black circles indicate residues that we have identified as being unique to both ScLDAP1 and AtLDAP1, suggesting potential involvement in ScLDAP1's WE-specific function, and AtLDAP1s lack of WE specificity.

While results from MDS predicted that there was a higher propensity for the identified loop (aa 40 to 45) of ScLDAP1 to bind to WE-filled monolayers compared with a bilayer, the observed AtLDAP1 capacity to also bind to WE-filled monolayers through the same region suggested that some other, additional portion of ScLDAP1 is responsible for facilitating WE partitioning out of the ER and into LDs. Consequently, we took advantage of the predicted, conserved multi-α-helix organization of the LDAP1 proteins with interrupted proline turns to swap peptide segments of the AtLDAP1 and the ScLDAP1 and evaluate the function of the resulting chimeric proteins (CPs) (Fig. 8). These CPs were then expressed in the *N. benthamiana*-based WE reconstitution assays to assess their capacity to promote proper WE exit from the ER. Initially, a pair of CPs were generated by swapping 2 halves of the protein: the N-terminal half of ScLDAP1 being replaced with the comparable region from AtLDAP1 (referred to as CP 1 and 2, respectively; Fig. 8A). Upon transient production of either CP with the WE biosynthetic enzymes, only the CP1 that contained the C-terminal half from ScLDAP1 had the ability to reduce the WE-induced swollen ER defects (Fig. 8A, top row). To assess if the full C-terminal half of ScLDAP1 or 1 or more pieces of this protein segment were required for function, additional CP3, CP4, CP5, CP6 made by swapping smaller C-terminal regions, based around conserved LDAP α-helices were generated and tested in the same manner (Fig. 8A). Upon CP co-expression with the WE synthesizing machinery, confocal imaging revealed that neutral lipid release from the ER relied on the presence of an α-helix in the ScLDAP1 spanning residues 156 to 187 (Fig. 8, A and B). This helix 6, colored yellow in the cartoon models of the LDAP proteins, sourced from ScLDAP1, but NOT from AtLDAP1, was both necessary and sufficient to reduce the abundance of the swollen ER regions induced by WE synthesis. CP production was confirmed by visualizing the mCherry fluorescence where the chimeric LDAPs each localized to LDs, further supporting separate functional regions on the LDAP proteins—1 part for targeting to LDs which is the same for each LDAP1, and 1 that is selective for WE partitioning that is unique to the ScLDAP1 helix 6.

**Figure 9.**
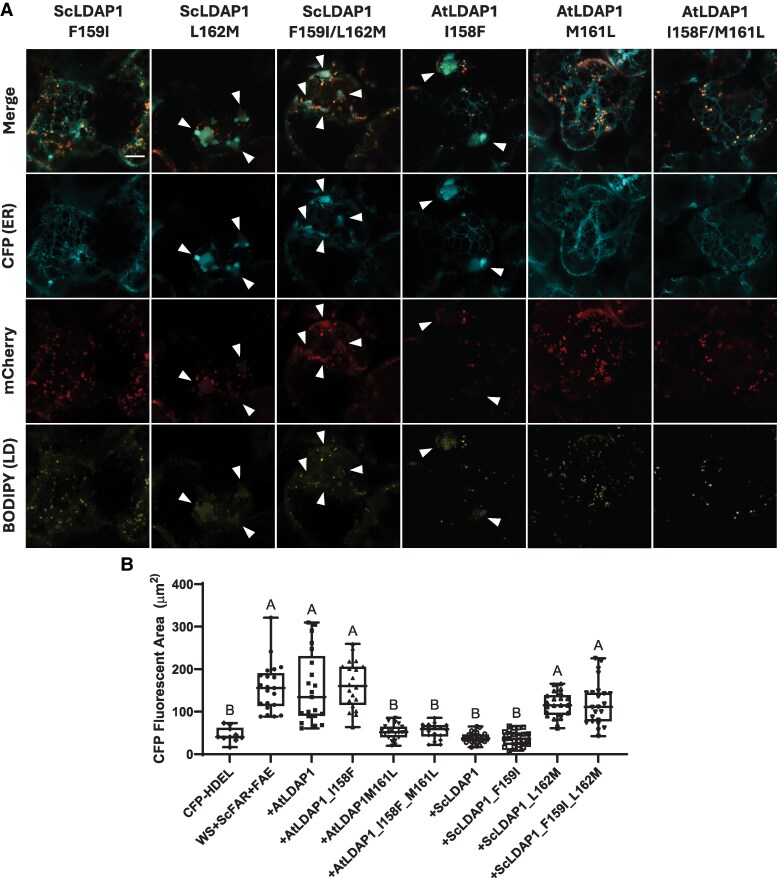
Mutant LDAP1 expression shows a gain and loss of WE specificity for both AtLDAP1 and ScLDAP1. **A)** Representative high-resolution AIRYSCAN images of infiltrated *N. benthamiana* leaves transiently expressing ScWS, ScFAR, ScFAE, a CFP-tagged ER marker (CFP-HDEL), and N-terminal mCherry-tagged mutant LDAP1. Samples were stained with BODIPY (493/503) to visualize neutral lipids. Arrows point to regions of ER defects. Scale bar corresponds to 10 *µ*m for all images. CFP-HDEL, cyan; BODIPY (493/503), yellow; mCherry, magenta. All samples were stained and imaged (Zeiss LSM710 confocal laser scanning microscope, fitted with AIRYSCAN attachment) at 4 d post infiltration. Mutation of AtLDAP1 residue 161 from methionine (M) to leucine (L) showed a gain of WE specificity, while the comparable mutation of ScLDAP1 residue 161 from L to M showed a loss of WE specificity. Mutations of AtLDAP1 residue 158 from isoleucine (I) to phenylalanine (F), and ScLDAP1 residue 159 from F to I did not show any effect on WE specificity. Double mutations of LDAP1 from both Arabidopsis and jojoba showed a similar gain and loss of function that was shown with the singular L and M mutations. LD, lipid droplet. **B)** Quantification of the CFP-fluorescent area (*µ*m^2^) associated with swollen ER defects. “Wax Synthesis” samples include the expression of P19 (endogenous silencing suppressor), CFP-HDEL, ScWS, ScFAR, and ScFAE collectively and with the mutant or WT LDAP1s. Values correspond to the multiple images collected from 3 separate infiltration experiments (with a minimum of 7 images from each replicate). Different letters indicate significant difference at *P* ≤ 0.05, as determined by Kruskal–Wallis test followed by Dunn's test. Whiskers of the box and whisker plot correspond to the upper 25% and lower 25% of values from the dataset. The upper lines and lower lines of the whiskers correspond to the maximum and minimum values of the dataset. The box corresponds to the middle 50% of the data with the line bisecting the box corresponding to the median of the data set. The top and bottom lines of the box correspond to the upper and lower quartiles, respectively.

To assist in the identification of potential features from ScLDAP1 helix 6 that could potentially be responsible for the WE-specific function, protein helical wheel projections and amino acid sequence alignments were compared for ScLDAP1 and AtLDAP1 (Fig. 8C). Helices from both LDAP1 proteins exhibited a strong amphipathic character, with similar overall hydrophobic and hydrophilic faces (Fig. 8C). Several residues were different between the 2 helices in terms of their position on the hydrophobic faces of the helices and the primary sequence (Fig. 8C). LDAP analysis by others have suggested that large hydrophobic residues present on the non-polar face of amphipathic helices played a key role in preferential associations with LDs (Prévost et al. 2018). Taking this into consideration, we selected the residues 159 (phenylalanine; F) and 162 (leucine; L) from jojoba and the comparable residues 158 (isoleucine; I) and 161 (methionine; M) from Arabidopsis to test for their function in facilitating the partitioning of WEs from the ER into LDs. N-terminal, mCherry-tagged single- and double-mutated versions of ScLDAP1 and AtLDAP1 coding sequences were generated and co-expressed in *N. benthamiana* leaves with the WE biosynthesis enzymes (i.e. untagged ScWS, ScFAR, ScFAE), as well as CFP-HDEL to visualize ER organization (Fig. 9). Mutations that swapped jojoba residues for Arabidopsis residues and vice versa included the following: ScLDAP1 F159I (ScL1-F159I), ScLDAP1 L162M (ScL1-L162M), ScLDAP1 F159I + L162M (ScL1-F159I/L162M), AtLDAP1 I158F (AtL1-I158F), AtLDAP1 M161L (AtL1-M161L), and AtLDAP1 I158F + M161L (AtL1I158F/M161L). Converting the jojoba residue from F to I did not change its partitioning function; however, changing the L to M appeared to disrupt the protein's ability to reduce the ER swelling (Fig. 9A, compare the first 2 columns). The double mutant, ScL1F159I/L162M, also had disrupted function, due to it also carrying the L162M mutation. Further, the inverse mutations in the AtLDAP1 corroborated these results. The single mutant I158F in the Arabidopsis sequence did not alter the protein function from wild-type (WT) AtLDAP1; i.e. the ER defects were still visible (Fig. 9, column 4). However, the single mutant M161L (and the double mutant containing the M161L) dramatically reduced the ER swelling and appeared to facilitate partitioning of neutral lipids out of the ER and into LDs in a manner similar to the jojoba native sequence (Fig. 9A, see last 2 columns). Enhanced-resolution confocal images were collected from 7 different leaf locations in each of 3 independent infiltration experiments and were quantified for CFP fluorescence (Fig. 9B). These combined data report a reproducible quantitative read-out of the ER swelling. For reference, the CFP fluorescence areas for the 2 native proteins are included in the quantitative plot (AtLDAP1 and ScLDAP1, Fig. 9B); for visual comparison also see Fig. 3A (last 2 columns). The confirmation of mutant protein production was assessed in each experiment by mCherry fluorescence, where the mCherry-tagged LDAP1 proteins all co-localized with BODIPY staining of LDs (Fig. 9A, bottom 2 rows). Taken together these results indicate that L162 within helix 6 on ScLDAP1 is responsible for WE selectivity, and ultimately the ability to more efficiently partition WEs into LDs. Further, these experiments support 2 functional regions of LDAP protein—1 for LD localization separate from 1 for WE selectivity.

### Structural and hydrophobic differences between WT and mutant LDAP1s

**Figure 10.**
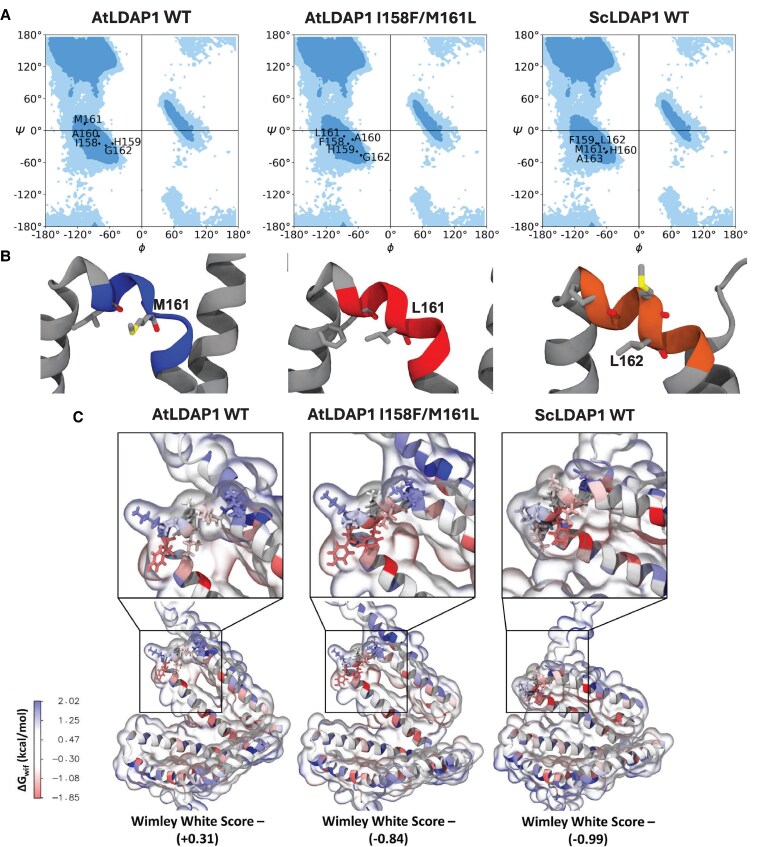
Structural and hydrophobic differences of WT and mutant LDAP1s. **A)** Ramachandran plot analyzing the ϕ and ψ angles of AtLDAP1 WT, AtLDAP1 I158F/M161L, ScLDAP1 WT. Plotted residues shown correspond to the first 5 residues of the sixth α-helix in each LDAP1. The blue and light blue regions represent the allowed and marginally allowed regions for dihedral angle distribution, respectively. AtLDAP1 WT residue M161 shows ϕ and ψ angles that lie outside the acceptable range for α-helices (−57° and −47°, respectively), indicating a breakage in helicity. ScLDAP1 WT, and AtLDAP1 I158F/M161L show ϕ and ψ angles of acceptable ranges. **B)** Selected regions of AlphaFold predicted AtLDAP1 WT (blue), AtLDAP1 I158F/M161L (red), and ScLDAP1 WT (orange) structures. Labels highlight residues identified as being critical for conferring the ability to more efficiently partition WEs. The presence of M161 in AtLDAP1 WT results in a disruption of helicity as suggested in Ramachandran plots (Fig. 10A), and this disruption is restored upon the mutation of M161 to a ScLDAP1 WT like L161. **C)** Hydrophobicity measurements using the Wimley–White scale of amino acids corresponding to the sixth α-helix in AtLDAP1 WT, AtLDAP1 I158F/M161L, and ScLDAP1 WT. Blue coloring of residues, or positive values (bottom) correspond to higher free energy values (hydrophilicity), while red or negative values indicate lower free energy values (hydrophobicity). Wimley–White score comparison of selected residues in the region of LDAP1s shown to be critical for WE-specific function shows ScLDAP1 and AtLDAP1 I158F/M161L as having a more favorable free energy value compared with AtLDAP1 WT, suggesting more favorable associations with the LD monolayer.

Both chimeric and site-directed mutation experiments point to a specific region of 1 amphipathic α-helix that appears to be responsible for ScLDAP1 WE selectivity. To provide a structural explanation for the differences in helix 6, we performed structural comparisons between the AlphaFold (Jumper et al. 2021) predicted AtLDAP1 and ScLDAP1 helices. Comparing the AlphaFold predicted structures in this region highlighted differences in secondary structure between Arabidopsis and jojoba helices (Fig. 10). In AtLDAP1, the secondary structure for residues between 159 and 163 is predicted to be a split helix-coil-helix motif. By comparison, in ScLDAP1 the secondary structure of helix 6 is predicted to be 1 continuous helix (Fig. 10A). By mutating the AtLDAP, M161L greater helicity was predicted (Fig. 10A). By visualizing the structure of the WT and mutant AtLDAP1 protein, the helicity breakage point is around the M161 residue (Fig. 10B). To classify any residue as part of an α-helix by the visualization software the ϕ and ψ angles should be near −57° and −47° respectively (Ramachandran and Sasisekharan 1968). Plotting the ϕ and ψ angles on a Ramachandran plot for the WT Arabidopsis, mutant and WT jojoba shows that the dihedral angle of M161 is the largest change in this region (Fig. 10A).

Beyond the slightly modified helical angle predictions described above, the double mutant that introduces the 2 jojoba residues into the Arabidopsis sequence also changes the overall hydrophobicity of this region. As measured by summing the Wimley and White (WW) hydrophobicity scale values (Wimley and White 1996) for the residues in the loop region, this specific place where the mutation is targeted is not particularly hydrophobic overall in AtLDAP1, with a sum score of +0.31 (Fig. 10C). By contrast, the ScLDAP1 is highly hydrophobic in this region, with a WW score of −0.99. This negative score implies that the protein would be more favorable in this region to interact with a hydrophobic environment. Just swapping the 2 jojoba residues into the ATLDAP1 protein (AtL1-I158F/M161L) was enough to change the WW score to negative (−0.84), similar to the native ScLDAP1 protein. These results suggest that the hydrophobicity of this specific loop region facilitates the selective interaction of ScLDAP1 with nascent LDs containing WEs and efficiently promotes the exit of WEs into LDs. It also explains why the AtLDAP1 is less effective in this functional role.

### Transgenic Arabidopsis lines co-expressing ScLDAP1 with WE biosynthetic enzymes show improved storage lipid compartmentalization

**Figure 11.**
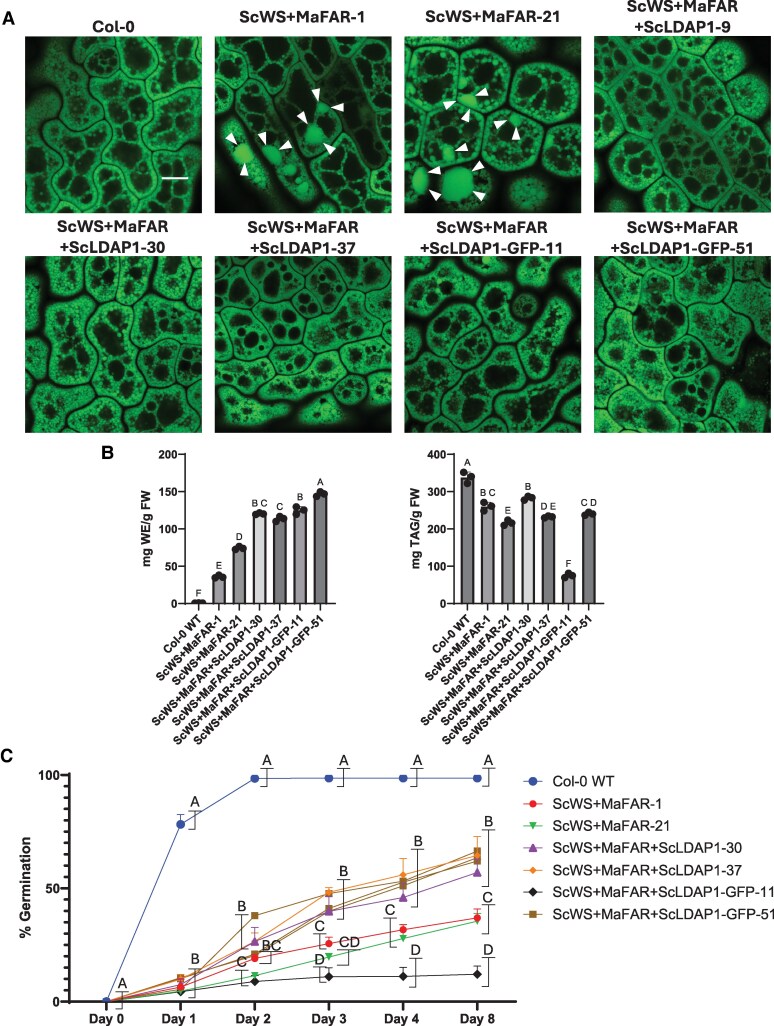
ScLDAP1 improves WE accumulation in stable transgenic Arabidopsis lines. **A)** Representative high-resolution AIRYSCAN images (single plane) of Arabidopsis seed embryos stably expressing ScWS, MaFAR, and ScLDAP1 or C-terminally green fluorescent protein (GFP)-tagged ScLDAP1 (ScLDAP1-GFP). Samples were stained with BODIPY (493/503) to visualize neutral lipids. Arrows point to regions of lipid aggregates. BODIPY (493/503), green. Scale bar represents 5 *µ*m for each image. AIRYSCAN imaging of Arabidopsis seed embryos expressing ScWS and MaFAR show large amorphous lipid-stained structures similar to those identified in *N. benthamiana* leaves expressing similar genes involved in WE synthesis. The production of ScLDAP1 in conjunction with ScWS and MaFAR resulted in a more uniform LD phenotype with no large lipid-stained structures. **B)** WE and TAG quantification of transgenic Arabidopsis seeds expressing ScWS and MaFAR with or without ScLDAP1. “Wax Synthesis” samples include the expression of either ScWS and MaFAR collectively and with either ScLDAP1 ScLDAP1-GFP. Quantification represents mean values of triplicates for each line (*n* = 3). Different letters indicate significant difference at *P* ≤ 0.05, as determined by 1-way ANOVA with Tukey's post-test. Error bars correspond to standard deviation. Sample names correspond to the genes stably expressed in said line, followed by the numerical identifier of that line (e.g. Alone-1 corresponds to ScWS + MaFAR-1). **C)** Percent seed germination of Arabidopsis seeds expressing either ScWS and MaFAR individually, or in conjunction with ScLDAP1. Seeds were considered to be germinating by radical emergence. Three replicate germinations of a minimum of 150 seeds per replicate were used per line. The number of germinated seeds was counted at 1-, 2-, 3-, 4-, and 8 d post exposure to light. Different letters indicate significant difference at *P* ≤ 0.05, as determined by 1-way ANOVA with Tukey's post-test. Error bars correspond to standard deviation. FW, fresh weight.

Previous studies for transgenic oil seed lines ectopically expressing WE synthesizing machinery, reported that WE accumulation led to deleterious side effects (Ivarson et al. 2017; Domergue and Miklaszewska 2022), including poor seed germination and disrupted neutral lipid packaging (Iven et al. 2016; Domergue and Miklaszewska 2022). Based on our results suggesting that ScLDAP1 supports WE partitioning out of the ER and into LDs, we tested whether ScLDAP1 could improve the neutral lipid packaging in transgenic seeds producing WEs. Therefore, transgenic Arabidopsis lines were generated expressing either ScWS and *Marinobacter aquaeolei* FAR (MaFAR, with a broader range of FAR activity than ScFAR [Willis et al. 2011]) by themselves, or co-expressed with untagged or C-terminal GFP-tagged ScLDAP1. Embryos from these transgenic lines were stained with BODIPY (493/503) and LD phenotypes visualized by enhanced-resolution confocal microscopy (AiryScan, Fig. 11). Similar to what was reported previously, transgenic lines expressing just the WE machinery showed a disrupted neutral lipid packaging in the form of large fluorescent-stained lipid structures that somewhat resembled the ER defects observed in *N. benthamiana* reconstitution assays. Germination percentages of these lines were also measured, and similar to what has been previously reported (Iven et al. 2013, 2016; Ivarson et al. 2017), they were shown to be severely reduced when WEs were accumulated (Fig. 11C).

Upon co-expression of either untagged- or GFP-tagged ScLDAP1 in transgenic Arabidopsis seeds, the disrupted lipid packaging was reversed to a more uniform LD population, comparable to the LD organization in seeds of the Col (0) non-transgenic background (Fig. 11A). WE and TAG levels within each transgenic line were also measured, and all lines expressing both ScLDAP1 and WE machinery showed increased WE accumulation when compared with the lines expressing only the WE machinery (Fig. 11B). However, expressing ScLDAP1 did not affect the composition of WE accumulated in the seeds (Supplementary Fig. S14). In addition to the improved subcellular compartmentation of storage lipids in the seeds, the percent germination of all but 1 of the lines expressing ScLDAP1 showed significantly higher germination percentages over an 8-d time course compared with those expressing just the WE synthesizing genes (Fig. 11C). This partial restoration of seedling development suggests that improved compartmentalization of neutral lipids afforded by ScLDAP1 may help reverse some of the negative effects of WEs on seed germination in transgenics, but also indicates that other factors yet to be identified will be required for a complete restoration of seedling establishment in transgenics designed to accumulate WEs in seeds. Overall, however, our results support an important role for ScLDAP1 in the effective WE packaging in plant cells.

## Discussion

Within the last decade, the list of proteins and the understanding of their mechanistic contributions to LD biogenesis in plants have expanded considerably (reviewed in Guzha et al. 2023). Certainly, the abundant oleosin proteins have long been known to be important for the stable compartmentalization of neutral lipids LDs in seeds and pollen, where cells undergo extreme desiccation and subsequent rehydration (Siloto et al. 2006; Miquel et al. 2014; Shimada et al. 2018; Gao et al. 2019). However, a growing appreciation that LDs accumulate in many tissues that do not express oleosin genes, and the awareness of conserved protein-mediated mechanisms for lipid storage across kingdoms (Huang 2018; Gao et al. 2019; Guzha et al. 2023), has prompted a further examination of the potentially distinct ways in which LDs are produced and function in plants. This growing understanding has revealed an increasingly elaborate mechanism for LD biogenesis that requires the cooperation between several protein complexes to allow for the efficient formation and directional LD release from the ER into the cytoplasm (Bouchnak et al. 2023; Guzha et al. 2023; Kumari et al. 2023; Zhao et al. 2023). In plants, several central players have been shown to play essential roles in LD biogenesis, including SEIPINs, LDIP, LDAPs, and VAP27-1, which has provided a general model for how these fundamental components work together to produce LDs in all types of tissues and cells. Additional accessory proteins contribute to LD stability (oleosins), intracellular LD distribution (SLDP and LIPA), LD turnover (SDP1, PUX10, CDC48A, MIEL1), and LD function (caleosins, steroleosins), depending upon cellular demands, plant tissue types, and/or developmental stage (Chen et al. 1999; Lin et al. 2002; Huang and Huang 2017; Deruyffelaere et al. 2018; Rahman et al. 2018; Krawczyk et al. 2022; Scholz et al. 2022; Bouchnak et al. 2023; Clews et al. 2023; Guzha et al. 2023; Traver and Bartel 2023). Here, we show that there are other subtle, but important, variations in the fundamental LD biogenetic components that contribute to LD formation/function, depending upon the type of neutral lipid that is synthesized and packaged in LDs. Specifically, our data point to a key amino acid residue in an amphipathic α-helix of LDAP1 from jojoba seeds (ScLDAP1) that is both necessary and sufficient to support the efficient packaging of newly synthesized WEs in a heterologous plant cell environment, supported by other regions of LDAP that bind to the LD surface. In work by others, transgenic oilseeds that were re-designed to synthesize WEs in their seeds showed aberrant LD morphologies with deleterious effects on seed germination and seedling growth. Given these atypical LD morphologies, it was proposed that the transgenic plants hosting the WE biosynthetic enzymes might lack the LD-related proteins required for efficient packaging of WEs into LDs (Ivarson et al. 2017; Sturtevant et al. 2020; Domergue and Miklaszewska 2022). Our results with Arabidopsis seeds co-expressing ScLDAP1 with the WE biosynthetic enzymes (Fig. 11) support this concept and suggest that LD packaging proteins in general are an important consideration for biotechnology strategies aimed at producing large amounts of high-value, non-native lipids in heterologous plant systems.

### ScLDAP1 facilitates the efficient packaging of WEs into LDs

LD proteins have varied roles in the formation, stability, and degradation of LDs (Renne et al. 2020; Guzha et al. 2023). Disruption of genes encoding most of these proteins has shown substantial impacts on lipid content, LD size, quantity, and/or location in vegetative and seed tissues (Cai et al. 2015; Gidda et al. 2016; Pyc et al. 2017b, 2021; Chung et al. 2019; Scholz et al. 2022). Our transient expression assays reconstituting WE biosynthesis in *N. benthamiana* leaves offered both visual and biochemical readouts for the effective partitioning, or lack thereof, of neutral lipids out of the ER and into LDs (Figs. 1 to 4). Even temporary overaccumulation of WEs resulted in disruptions in ER organization and the retention of neutral lipids in the ER rather than export to LDs, and this was only alleviated by co-expression of ScLDAP1 (Figs. 2 and 3). By contrast, the LDAP isoforms from Arabidopsis or *N. benthamiana* were ineffective at promoting the partitioning of the WEs out of the ER in these transient assays (Figs. 3 and 4, Supplementary Figs. S3 and S4).

Recent studies have shown that packing defects in the monolayer surface are sensed by amphiphilic α-helices of some LD proteins, with some also showing differential affinity toward different neutral lipid classes (Chorlay and Thiam 2020; Shivaiah et al. 2023). For example, differential surface affinities by helical segments from various LD proteins were demonstrated for synthetic squalene-containing LDs, triolein-containing LDs, or sterol ester-containing LDs, indicating that packing defects at the monolayer surface, the underlying neutral lipid composition, and the nature of the amphipathic α-helix amino acids, all act to facilitate the association of LD proteins to monolayer rather than bilayer surfaces (Chorlay and Thiam 2020). Further, two-thirds of 42 plastoglobuli proteins identified in a proteomics study contain an amphipathic α-helix, which was suggested to mediate the specific interaction of these proteins to the monolayer surface of the plastoglobuli and not to the bilayer surface of the thylakoid membrane (Shivaiah et al. 2023).

Our MDS of monolayer surfaces with specific phospholipids and neutral lipid compositions, taken from jojoba seeds (Sturtevant et al. 2020), *N. tabacum* leaves (Ruzicska et al. 1983; Ishida 2013), or *N. benthamiana* leaves (Supplementary Table S1), demonstrated that there were packing defects noted by the transient appearance and disappearance of WEs and TAGs at the monolayer surface over 1,000 ns simulations (Supplementary Fig. S7, Videos S1, S2, S51 and S52). Comparing multiple MDS interactions of AtLDAP1 and ScLDAP1 with monolayer and bilayer surfaces indicated that both proteins interacted more strongly with monolayers versus bilayers with consistent penetration of a helix-loop region between amino acids 40 and 45 into the monolayer surface (Fig. 6A, arrows, Fig. 7). ScLDAP1 showed preferential binding to the WE-filled monolayers, compared with TAG-filled monolayers. From molecular dynamics, we predict that the penetration of a helix-loop region into the membrane surface could be part of a possible binding mechanism of LDAP1 with monolayers. We recognize that there are limitations with simulation experiments and that these predictions from MDS will need to be experimentally validated with future studies. Moreover, actual 3D structural determinations for a plant LDAP1 protein in the future might help uncover additional mechanistic details of how the LDAP1 proteins could support the partitioning of neutral lipids out of the ER.

While a canonical LD targeting sequence has been difficult to generalize, it has been shown that for a majority of Class 2 type LD proteins, localization to the LD is facilitated in part by the presence of amphipathic α-helices (Čopič et al. 2018; Prévost et al. 2018). Previous studies with LDAP1 from Arabidopsis showed that removing any part of the LDAP protein disrupted its localization to LDs in situ (Gidda et al. 2016). Further, liposome-binding assays were insufficient to support a specific surface lipid-binding mechanism; however, no “monolayer” membrane was used to test the binding affinity of AtLDAP1, only different phospholipid compositions reflective of subcellular compartments (including LDs). Hence, it may be that the helix-loop region identified here by MDS near the N-terminus of both AtLDAP1 and ScLDAP1 (Fig. 6A), could represent the actual region of LDAP proteins that is important for LD-specific targeting, a property that would be required for either of these proteins to function properly in its in vivo environment. However, this would need to be supported by further LD targeting studies.

On the other hand, and contrary to our expectations, the MDS studies here did not indicate a differential selectivity of ScLDAP1 over AtLDAP1 for the monolayer surface (with underlying WEs) at any other part of the protein. It is possible that protein-protein interactions at the surface via other LDAP proteins and/or the LDIP, might help to drive selectivity, which would not have been detected in our MDS studies. Notably, LDAP proteins are known to interact with each other and also with LDIP (Pyc et al. 2017b), and the efficient WE partitioning out of the ER required LDIP (Fig. 5), so a protein-protein-mediated mechanism is a reasonable possibility. Alternatively, a selective WE-specific interaction(s) may occur over a longer time scale than the microsecond time frame simulated here. Consequently, an unbiased series of helix-swapping experiments followed by site-directed mutational analysis was implemented to gain further mechanistic insights into the nature of the selective partitioning activity of ScLDAP1 for WEs.

### A key residue in an amphipathic α-helix confers WE specificity to ScLDAP1 in vivo

ScLDAP1 was predicted to be comprised largely of α-helices (e.g. Fig. 10, Supplementary Fig. S15), and we postulated that 1 or more of these helices in ScLDAP1 may have uniquely evolved to have WE selectivity. CPs comprised of various portions of ScLDAP1 and AtLDAP1 showed that 1 distinct amphipathic α-helix was important to allow for the proper packaging of WEs (Fig. 8). Analyses by others showed that the residue composition of the hydrophobic face of amphipathic α-helices (as well as overall hydrophobicity of this face) determines the degree of affinity of an LD protein toward packing defects that occur during the formation of LDs, and that subtle changes in residue composition could dramatically alter this affinity (Prévost et al. 2018). Based on our results, increased hydrophobicity through a single residue mutation was sufficient to allow AtLDAP1 to function like ScLDAP1 (Fig. 9). Introducing the mutation altered the predicted secondary structure in the AtLDAP1 helix to 1 more like that of ScLDAP1 (Fig. 10) which may affect residue positioning and ultimately association toward a WE-containing LD surface. Although speculative, a more hydrophobic helix might confer a more rapid association with a region of the ER where WEs are accumulating and promote a more efficient packaging process, perhaps by accelerating the binding of additional LDAP1 proteins or other protein partners, and/or a more pronounced curvature that would facilitate exit of WEs from the ER. In addition, the greater hydrophobicity of WEs compared with TAGs may highlight a need for an equally hydrophobic LD associating protein to stabilize the monolayer surface during early LD formation. It is likely that a small evolutionary change in ScLDAP1 accompanied the plant's ability to efficiently store WEs in its seeds, a storage lipid class that is not widely found in other plants. It would be of interest to further explore potential coevolution for LD packaging and storage lipid biosynthetic machinery, particularly given the large range of neutral lipids that occur across the plant kingdom (Ohlrogge et al. 2018). Further, it is important to emphasize that this change in ScLDAP1 hydrophobicity and functionality was a feature not revealed by simple sequence comparisons, but rather was context specific in an amphipathic helix, a situation that is perhaps primed for algorithmic datamining in the future. For example, amino acid sequence alignments of LDAP1 family members reveal only 2 other proteins with an equivalent “L162” in the amphipathic helix 6 of ScLDAP1, and these are proteins from legumes (soybean and cowpea) that do not store WEs in their seeds. So, it is likely that the helicity/hydrophobicity context is probably just as important for the ScLDAP1 WE-specific function as the specific residue, L162. Future computational approaches that consider the structural and physical properties of amphipathic helices in LDAP proteins may uncover other packaging-selective isoforms of LDAPs. In addition, whether LDAPs or other LD surface-associated proteins have co-evolved with the LD biosynthetic machinery to efficiently compartmentalize various lipid types in plants is fertile ground for future investigation.

### A model for ScLDAP1 function

**Figure 12.**
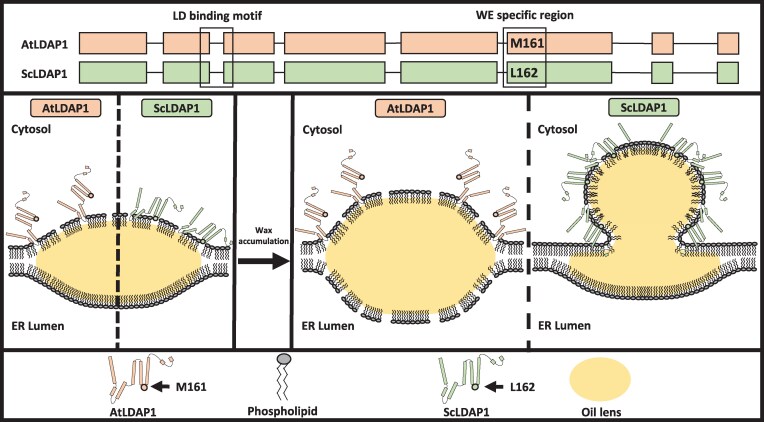
Model for ScLDAP1 WE-specific function. The model depicts the proposed effects of ScLDAP1 and AtLDAP1 on WE LD formation. Here both AtLDAP1 and ScLDAP1 are depicted as associating to phospholipid packing defects on the surface of nascent LDs through a conserved N-terminal loop (LD binding motif, upper left) corresponding to residues 40 to 43 in AtLDAP1 and 42 to 44 in ScLDAP1 (middle left). In instances where only AtLDAP1 is present, further WE accumulation proceeds, but remains trapped in the ER (middle) due to the presence of a less hydrophobic residue (M161) and disrupted helicity of helix 6 in AtLDAP1 (Fig. 10). In contrast, ScLDAP1 with its more hydrophobic residue (L162) and intact helix (Fig. 10) allows for secondary interaction with the packing defects, reducing membrane surface tension and allowing for the correct packaging of WEs into LDs, facilitating their export from the ER (right).

Taken together, our results suggest 2 separate regions that would allow for ScLDAP1 targeting and function. The first region identified by MDS near the N-terminus suggests a conserved loop (between amino acid residues 40 and 45) is required for the initial targeting and insertion of LDAPs to a monolayer surface (Figs. 6 and 7), while the region identified through mutational analysis (M161L in AtLDAP1, Figs. 8 to 10) is tuned toward facilitating WE partitioning from the ER to cytoplasmic LDs. A speculative model, based on our results, is shown in (Fig. 12) and suggests a WE-specific function of ScLDAP1. Here, both AtLDAP1 and ScLDAP1 are depicted as capable of associating with, and binding to, WE-filled nascent LDs through a conserved N-terminal loop region shared between both proteins (amino acid residues 40 to 45; Fig. 12, left panel). Upon further WE accumulation within the ER bilayer and the appearance of packing defects on the nascent LD surface, the ScLDAP1 residue L162, shown to be specific to WEs, associates with and potentially interacts with those defects (visualized in Supplementary Fig. S7), likely reducing membrane surface tension and promoting vectoral budding of the nascent LD (Fig. 12, right). AtLDAP1, with its less hydrophobic residue M161 and disrupted helicity in helix 6 (Fig. 10), is consequently less efficient at associating with and covering defects of the underlying WEs which likely leads to higher surface tension and less efficient LD formation (Fig. 12, middle). This results in the retention of WEs in the aberrant, large lipid-stained structures trapped in the ER (Fig. 12, middle). Although not described in this model, both AtLDAP1 and ScLDAP1 appear to be adequately suited to recognize defects that occur when just TAGs accumulate in LDs, and both proteins facilitate the release of TAG-filled monolayers from the ER albeit perhaps with seemingly different affinities/efficiencies (Supplementary Fig. S5). While this hypothetical model in Fig. 12 is consistent with our experimental results, there are several questions that remain. For example, does SEIPIN play an important role in WE storage? We presume so, since LDIP appears to be required for ScLDAP1 function, and LDIP and SEIPIN are known to cooperate in the release of TAG-filled monolayers from the ER (Pyc et al. 2021). Are there other proteins or factors that participate in this process or that would further improve WE accumulation? How extensive can this concept of selective recognition/packaging be extrapolated to other types of neutral lipids synthesized in the ER in plants, such as rubber, terpene esters, phenolic esters, and sterol esters? Addressing these questions and others could provide valuable insights into not only the fundamental cell biology of lipid storage, but also may have important applications in the overall accumulation of energy-dense, high-value lipids in plants.

## Materials and methods

### Plant materials

*N. benthamiana* plants used in transient expression experiments were germinated and grown in soil at 28 °C under 16-h/8-h day–night cycles. Leaves of 4 to 5-wk-old *N. benthamiana* plants were infiltrated with the *Agrobacterium tumefaciens* strain GV3101 harboring the appropriate binary vectors. For all infiltrations, *A. tumefaciens* harboring the Tomato bushy stunt virus (TBSV) gene, P19, was included to suppress gene silencing and enhance transgene expression, except for infiltrations that utilized NbLDIP RNAi which instead utilized the *Tomato yellow leaf curl virus* (TYLCV) gene V2 to facilitate the suppression of gene silencing (Naim et al. 2012). *A. tumefaciens* transformation, growth, infiltration, and preparation of infiltrated material for microscopic analysis have been described previously in (Petrie et al. 2010). For confirmation of both RNAi suppression as well as transgene expression, qualitative RT-PCR was used (Supplementary Figs. S6 and S16). Samples from mock infiltrated, as well as agrobacterium-infiltrated leaves were collected, flash frozen, and RNA extracted using the QIAGEN RNeasy Plant mini kit (QIAGEN, Inc., Valencia, CA, USA) following the manufacturer's protocol. One hundred nanograms of RNA was then used to generate cDNA utilizing the SuperScript III First-Strand Synthesis System following the manufacturer's protocol. Approximately 50 ng of cDNA was then used as template sequences for PCR to confirm the presence of transgene transcripts. PCRs were performed using Promega GoTaq Green Master Mix (Promega, Madison, WI, USA) following the manufacturer protocol. The following thermocycler protocol was used: 98 °C for 1 min, 35 amplification cycles (98 °C for 10 s, 55 °C for 15 s, 72 °C for 60 s), and 72 °C for 7 min. Sequences of primers used in the reactions are listed in Supplementary Table S2. Control reactions without reverse transcriptase confirm that transcripts and not genomic DNA fragments were amplified.

Experiments involving Arabidopsis seeds and seedlings employed WT Columbia (Col-0) ecotype, as well as transgenic lines overexpressing the genes involved in the synthesis of WEs and/or ScLDAP1 generated from Col-0 ecotype. The Arabidopsis transgenic lines were obtained via floral dipping (Clough and Bent 1998) using *A. tumefaciens* strain GV3101 and harboring binary vectors containing WE synthesis and ScLDAP1 genes. T1 plants with transgene expression were selected by spraying 7-d-old seedlings with 0.05% (w/v) Basta solution. T3 seeds were used for lipid analyses. Percent germination assays were performed according to the protocol described in Lindsey et al. (2017). Approximately 10 mg of seeds were sterilized in a 50% bleach solution for ∼10 min. The bleach solution was removed, and seeds were then washed 6 times with sterile water prior to being plated on ½ Murashige and Skoog (MS) plates. Plates were then covered with aluminum foil and stratified at 4 °C for 72 h. Plates were then transferred to 23 °C growth chambers and foil was removed to initiate germination of seeds. Germinating seeds were then counted based on radicle emergence at 1-, 2-, 3-, 4-, and 8 d after the initiation of germination.

### Plasmid construction

Coding regions for the cloning of jojoba LD proteins and ScFAE were isolated from cDNA generated from jojoba seeds. RNA was purified from mature jojoba seeds according to the protocol described in (Sturtevant et al. 2020). Approximately 100 ng of purified RNA was used to generate cDNA utilizing the SuperScript One-Step RT-PCR system (Invitrogen). ScWS, ScFAR, ScSeipin1, and ScSeipin2 were synthesized by the DNA synthesis services provided by GenScript Inc. Forward and reverse primers were utilized to amplify coding regions and are listed in Supplementary Table S2. Restriction enzymes AscI and PacI (NEB) and T4 ligase (NEB) were used to insert amplified DNA into the plant expression vectors pMDC32 and pMDC32mCherry (Curtis and Grossniklaus 2003). CPs were generated utilizing overlap extension PCR (Hilgarth and Lanigan 2020). Fragments used for overlap extension were amplified from vectors containing either AtLDAP1 or ScLDAP1 utilizing primers listed in (Supplementary Table S2). Fragments were then fused using an Overlap PCR and cloned into the plant expression vector pMDC32mCherry (Curtis and Grossniklaus 2003) using the restriction enzymes AscI and PacI (NEB), and T4 ligase (NEB). AtLDAP1 and ScLDAP1 mutants were synthesized by the DNA synthesis services provided by Gene Universal Inc. Genes were amplified using the appropriate forward and reverse primers from vectors containing synthesized genes, and cloned into pMDC32mCherry (Curtis and Grossniklaus 2003) using the restriction enzymes AscI and PacI (NEB) and T4 ligase (NEB). The binary vectors for simultaneous expression of genes encoding MaFAR, ScWS, and ScLDAP1 were generated using the Gateway technology (Thermo Fisher Scientific) as previously described (Heilmann et al. 2012). To construct the entry vectors, the coding sequences for ScWS, ScLDAP1, and ScLDAP1-GFP were amplified from plasmids pBinGlyRed-FWS3 (Miklaszewska and Banaś 2016), pMDC32, and pMDC84, respectively, using Phusion High-Fidelity DNA polymerase (Thermo Fisher Scientific) and primers listed in Supplementary Table S2. The amplified sequences were cloned into the SalI/BamHI restriction sites of either pENTRY-C5 (ScWS) or pENTRY-A4 (ScLDAP1, ScLDAP1-GFP). The resulting constructs were sequenced and used in combination with pENTRY-B6 containing YFP:myc:MaFAR (Vollheyde et al. 2020) to yield the binary vectors: pCAMBIA33-βcon::YFP:myc:MaFAR/gly::ScWS,pCAMBIA33-nap::LDAP1/βcon::YFP:myc:MaFAR/gly::ScWS, pCAMBIA33-nap::LDAP1:GFP/βcon::YFP:myc:MaFAR/gly::ScWS. The correct assembly of the vectors was confirmed by sequencing. The promoters driving expression of transgenes were as follows: ScWS, soybean glycinin promoter (gly); MaFAR, soybean β-conglycinin promoter (βcon); ScLDAP1, *Brassica napus* napin promoter (nap) (Park et al. 1992; Rask et al. 1998; Dean and Finer 2023).

### Microscopy

Agrobacterium-infiltrated *N. benthamiana* leaves expressing heterologous genes were prepared for confocal microscopic analysis 4 d post infiltration as previously described in Cai et al. (2015). Micrographs of *N. benthamiana* leaves were captured by using a Zeiss LSM710 confocal microscope retrofitted with an AiryScan detector head. Images were acquired with either a 63× oil immersion objective lens (NA = 1.4), or a 40× water immersion objective lens (NA = 1) and an Ar-ion laser (Carl Zeiss Inc.). Images of leaf cells were acquired as either Z-stacks (0.5 *µ*m z-sections, 25 sections total) or as single optical plane images, and saved as 1,024 × 1,024-pixel digital images. AiryScan images were acquired as single optical plane images (0.25 *µ*m thick) and saved as 1,240 × 1,240-pixel digital images. BODIPY 493/503 a neutral lipid-specific dye (Spangenburg et al. 2011), and chlorophyll autofluorescence were excited with a 488 nm laser, CFP with a 405 nm laser, and mCherry with a 563 nm laser. For all lasers, laser intensities were set to 2% of the total laser power. Emission spectra for the above-listed fluorophores were collected as follows: 500 to 540 nm for BODIPY fluorescence, 590 to 640 nm for mCherry, 450 to 490 nm for CFP, and 650 to 750 nm for chlorophyll autofluorescence. For all images, the master gain was set within the range of 725 to 750 depending on the fluorescence signal in the samples. For neutral lipid visualization samples were stained with 2 *µ*g/mL BODIPY 493/503 (prepared from a stock solution of 4 mg/mL dissolved in DMSO) in 50 mm PIPES buffer (pH 7). Images were taken from at least 2 separate infiltrated leaves across 3 individual infiltration experiments. Per sample, a minimum of 15 images were taken with representative images being shown. For CFP fluorescence area quantification, the ImageJ 1.54f plugin, 3D objects counter was used to quantify CFP fluorescence area associated with ER defects (Schindelin et al. 2012). A minimum of 15 images across 3 infiltration experiments were used for CFP area quantification.

Transgenic seeds were prepared for confocal microscopic analysis of LDs by first imbibing dry seeds in water for 20 to 40 min to soften the seed coat. Seed coats were then removed by gently rolling them between the glass slide and cover slip. Seed embryos were then added to a fixative solution (4% paraformaldehyde in 50 mm PIPES pH 7.0) and incubated on a rotational shaker at 100 rpm for 20 min. The fixative solution was removed, and embryos washed 3 times with a solution of 50 mm PIPES pH 7.0 for 10 min each wash before being transferred to a dye solution (BODIPY 493/503 2 *μ*g/mL in 50 mm PIPES, pH 7.0) to stain for 20 min on a rotational shaker at 100 rpm covered from the light. The staining solution was removed, and embryos were washed 3 times with a wash solution of 50 mm PIPES pH 7.0 for 10 min on a rotational shaker at 100 rpm covered from the light. Stained seed embryos were then transferred to slides and analyzed using a Zeiss LSM710 confocal microscope retrofitted with an AiryScan detector head. BODIPY 493/503 was excited by a 488 nm laser, and the emission signal was collected from 500 to 540 nm. Laser intensity was set to 2% of the total laser power.

### LD and microsome isolation

For LD and microsome isolations ∼2.5 g of infiltrated *N. benthamiana* leaves taken from plants 4 d post-infiltration were homogenized in 5 mL of ice-cold buffer 600 mm sucrose buffer (600 mm sucrose, NaH_2_PO_4_, pH 7.5, 150 mm NaCl, 0.1% (v/v) Tween-20, 1 mm PMSF, 1× cOmplete Protease Inhibitor Cocktail (Roche)). The resulting supernatant was then transferred to 15-mL centrifuge tubes. 2.5 mL of ice-cold 400 mm sucrose buffer (400 mm Sucrose, 10 mm NaH_2_PO_4_, pH 7.5, 150 mm NaCl, 0.1% (v/v) Tween-20, 1 mm PMSF, 1× Complete Protease Inhibitor Cocktail (Roche)) was then layered on top of leaf homogenate and samples centrifuged at 10,500 × *g* for 60 min at 4 °C to float LDs. The floated LDs were then carefully transferred to fresh tubes for either lipid extraction, or microscopic analysis, while the remaining supernatant was transferred to ultracentrifuge tubes and centrifuged and 100,000 × *g* for 60 min at 4 °C. The resulting supernatant was then removed, and the microsomal pellet resuspended in fresh 400 mm sucrose buffer and either used for lipid extractions or microscopic analysis.

### Lipid extraction and analysis

For total lipid extraction, ∼5 g of infiltrated *N. benthamiana* leaves taken from plants 4 d post-infiltration was flash frozen with liquid nitrogen, and then lyophilized overnight to remove water and prevent metabolic degradation of lipids by endogenous lipases. A portion of the lyophilized tissues (∼50 to 70 mg) were then measured into homogenization tubes prefilled with hexane-washed glass beads, and 40 nmol of both a WE standard heptadecanoyl-heptadecanoate (17:0/17:0) (Nu-check Prep Inc.), and TAG standard glycerol triheptadecanoate (17:0/17:0/17:0) (Nu-check Prep Inc.) were added. A 70 °C solution of 2-proponal with the addition of 1% BHT (w/v) was added, and tissues were disrupted for 1 min using a Glen Mills Mini-Beadbeater-16. Homogenized tissue was transferred to new tubes and incubated at 70 °C for 30 min in a hot water bath. Samples were removed and allowed to cool to room temperature before 1 mL of CHCl_3_ was added with 400 µL of water to establish a ratio of solution of 2:1:0.45 2-propanol/CHCl_3_/H_2_O (v/v/v) before being transferred to a 4 °C fridge overnight. Total lipids were separated into an organic layer through the addition of 1 mL of CHCl_3_ and then washed 2× with 1 M KCl, with the aqueous layer removed after each wash. The organic layer was then transferred to new tubes, and concentrated under a stream of N_2_, before being resuspended in 1 mL of CHCl_3_. The resulting total lipid fraction was then added to SPE columns (Discovery DSC-Si SPE Tube) for separation of the neutral lipid fraction from polar lipids. For SPE fractionation of the neutral lipids, SPE columns were first washed with 5 mL of acetone and then conditioned with 2× washes of 5 mL of hexane. Total lipid fractions were then added and allowed to enter the column. For neutral lipid elution 6 mL of 4:1 hexanes/diethyl ether was added and washed through the column. The samples were then concentrated under a stream of N_2_ and resuspended once more in 1 mL of CHCl_3_ for storage before being prepped for MS analysis. For isolation of lipids from LD and microsomal fractions, lipids were extracted according to the protocol described in (Bartels and Dörmann 2021).

For MS analysis lipid extracts were dried under a stream of N_2_ and then dissolved in 1 mL of methanol:chloroform (2:1, v/v) containing 5 mm ammonium acetate. Lipid extracts were analyzed on a Waters SYNAPT G2-si Mass Spectrometry System by direct infusion. Samples of each extract were analyzed using the following parameters: positive ionization mode, voltage of 2.5 kV, back pressure of 0.5 psi, source temperature of 40 °C, and curtain gas set to 10 (arbitrary units). Data was collected using the MassLynx software. WE signals were selected using a 0.01–atomic mass unit window, a minimal signal-to-noise ratio of 1.0, and a maximum intensity of 0%. Data were processed using the mMass mass spectrometry tool. Samples were normalized against tissue dry weight, and relative quantification was calculated based on the internal standard response. Confirmation of WE peaks was achieved using MS/MS fragmentation utilizing the same instrument and settings at a collision voltage of 5, 10, and 20 kV. The resulting fragmentations were then compared with published fragmentation patterns of WEs to confirm WE ID (Iven et al. 2016).

Lipid extraction from Arabidopsis seeds was performed as described previously (Iven et al. 2013). Briefly, 1.5 mg (for ESI-MS/MS analysis) or 5 mg (for GC-FID analysis) seeds were homogenized in 1 mL methanol with a glass rod in an 8-ml glass tube. After homogenization, 1 mL of chloroform was added to each sample. For ESI-MS/MS analysis, 5 nmol heptadecanoyl-heptadecanoate (Nu-Chek Prep, Inc., Elysian, MN, USA) was added to each sample as an internal standard. For GC-FID analysis, 50 *µ*g di-17:0 WE and 100 *µ*g tri-15:0 TAG were added. Lipids were extracted by shaking for 20 min at 4 °C. Non-soluble cell fragments were pelleted by centrifugating at 450 × *g* for 5 min. The supernatant was transferred into a new 8-mL glass tube, and the pellet was re-extracted with 1 mL *n*-hexane:diethyl ether:acetic acid (65:53:1, v/v/v) for 10 min at 4 °C with shaking. After centrifugation, the supernatants were combined and evaporated under streaming nitrogen. The lipid extracts were dissolved in 40 *µ*L chloroform and separated by thin-layer chromatography (TLC) on 0.25 × 20 × 20 cm F60 (Merck KGaG, Darmstadt, Germany) silica gel glass plates using *n*-hexane:diethyl ether:acetic acid (80:20:1, v/v/v) as a solvent system. The lipid bands were visualized under UV light after spraying the plate with 0.2% (w/v) 8-anilino-1-naphthalenesulfonic acid or primuline (0.05% w/v in acetone/water, 80:20 v/v). For ESI-MS/MS measurement, WE bands were scraped from the plate and extracted twice with 1 mL *n*-hexane. The resulting supernatant was evaporated under streaming nitrogen. The WE fractions were dissolved in 2 mL methanol: chloroform (2:1, v/v) containing 5 mm ammonium acetate and diluted 100-fold. WE molecular species profiling was done according to the protocol described previously (Iven et al. 2013). For GC-FID analysis, WE and TAG bands were scraped from the plate, directly used for acidic methanolysis, and quantified as described in Iven et al. (2016).

### Molecular modeling

To directly compare the binding of the LDAP1s to WE-filled and TAG-filled monolayers, we first built the molecular models from Sc- and AtLDAP1 sequences using AlphaFold2 (Jumper et al. 2021) (Supplementary Fig. S15). Prior to simulation, the protonation states for the Sc- and AtLDAP1 proteins were analyzed using PROPKA (Olsson et al. 2011). Based on the predicted protonation states, in both ScLDAP1 and AtLDAP1 residue D78 was protonated and in ScLDAP1 residue K39 was deprotonated.

### Molecular assembly

With the end goal of comparing the bound structures for the different LDAP1 proteins to biological membranes, we used CHARMM-GUI (Jo et al. 2008) to develop 6 different membrane models, creating bilayer, WE-filled monolayer, and TAG-filled monolayer models with a membrane composition like that of either jojoba or *N. tabacum*. Membrane components for the bilayer and filled monolayer models were generated from published phospholipid compositions (Gülz and Eich 1982; Ruzicska et al. 1983), published WE compositions (Sturtevant et al. 2020) and compositions taken from this study (*N. benthamiana*), and published TAG compositions (Ishida 2013; Sturtevant et al. 2020; Supplementary Table S1). For the jojoba membrane models, the lipid head group composition was a 45:38:10:7 ratio of phosphatidyl choline (PC), phosphatidyl ethanolamine (PE), phosphatidyl inositol (PI), and phosphatidyl glycerol (PG) (Supplementary Fig. S8A). *N. tabacum* bilayer headgroup ratio was a 60:59:37:26:15:3 mixture of PC, PE, phosphatidic acid (PA), PG, PI, and phosphatidyl serine (PS) (Supplementary Fig. S8B). For the monolayer mimics that feature a bilayer bisected by a layer of WEs, we used CHARMM-GUI to build a WE layer from an initial template of bis-monoacylglyceryl phosphate (BMGP), and TAG layer was built by TAG lipids found in CHARMM-GUI. BMGP was modified to WE using CHARMM patch scripts and VMD patch commands (Brooks et al. 1983; Humphrey et al. 1996). The initial BMGP tails were modified into different tail lengths and degrees of unsaturation given in Supplementary Table S1. The resulting WE bilayer was simulated in a water box to generate a relaxed conformation for the WEs (Supplementary Fig. S9). Some of the TAG tails were also elongated, and unsaturation sites were inserted using VMD patch commands (Brooks et al. 1983; Humphrey et al. 1996) to match observed TAG structures found in *N. tabacum*, and *jojoba* (Ishida 2013; Sturtevant et al. 2020). The relaxed WEs and TAGs were inserted between the jojoba and *N. tabacum* bilayer, respectively, by creating a 30 Å gap between the upper and lower membrane leaflets mimicking the monolayer of LD surrounding WEs (Supplementary Fig. S10). The resulting model does lack the curvature of LD but it can mimic the LD surface by separating the hydrophobic core from the water surface. This methodology has been previously used by other groups to do all-atom MDS studies of LDs (Kim et al. 2022; Sapia and Vanni 2024). The monolayer system was equilibrated for 1 *μ*s to ensure stability of the system (Supplementary Fig. S17).

After creating an equilibrated system of monolayer, WEs and TAGs, the LDAP1 protein from Arabidopsis, and jojoba, was placed 50 Å above the center of monolayer (WEs + monolayer, TAG + monolayer) and 40 Å above the center of bilayer. Six replica simulations were run each with a different orientation of LDAP1 protein; 4 orientations were rotated around the *x*-axis 0, 90, 180 and 270 and 2 were rotated around the *y*-axis at 90 and −90 (Supplementary Fig. S18). The topologies of protein, LD membrane, WEs, TAGs, and solvent were merged using TopoTools (Kohlmeyer and Vermaas 2017). The system was re-solvated with the solvate command in VMD (Humphrey et al. 1996).

### Molecular dynamics protocol

The 72 simulation systems (6 independent protein orientations, 2 LDAP1 proteins, 2 different headgroup compositions, and 3 different membrane cores) were simulated by NAMD 3.0a9 (Phillips et al. 2020) using the CHARMM36m (Huang et al. 2017) force field for proteins and lipids. Simulations were performed in the NPT ensemble. Pressure was controlled using the Langevin piston method (Feller et al. 1995) at 1 atm. The temperature was maintained at 298 K (25 °C) using a Langevin thermostat with 1 ps^−1^ damping. SETTLE algorithm was used to fix hydrogen bond length in the simulation system (Miyamoto and Kollman 1992), allowing 2 fs timesteps. Long-range electrostatic interactions were handled using particle mesh Ewald (PME) (Essmann et al. 1995) grid with each grid point placed at 1.2 Å. Short-range non-bonded interaction was calculated at a cutoff of 12 Å (Darden et al. 1993). Each system was minimized for 1,000 steps and further equilibrated for 50 ps using the same condition with margin adjustment within 7 Å. After equilibration margin adjustments were removed and each bilayer system and TAG-filled layer was simulated for 1 *µ*s, while the simulations with the central WE layer were extended to 2 *µ*s. Videos of each simulation of both LDAP1s in each starting orientation are as follows: Supplementary Videos S3 to S8, MDS of AtLDAP1 with a jojoba like bilayer; Supplementary Videos S9 to S14, MDS of ScLDAP1 with a jojoba like bilayer; Supplementary Videos S15 to S20, MDS of AtLDAP1 with a jojoba like WE-filled monolayer; Supplementary Videos S21 to S26, MDS of ScLDAP1 with a jojoba like WE-filled monolayer; Supplementary Videos S27 to S32, MDS of AtLDAP1 with a *N. tabacum-*like bilayer; Supplementary Videos S33 to S38, MDS of ScLDAP1 with a *N. tabacum*-like bilayer; Supplementary Videos S39 to S44, MDS of AtLDAP1 with a *N. tabacum-*like phospholipid monolayer, filled with *N. benthamiana-*like WEs; Supplementary Videos S45 to S50, MDS of ScLDAP1 with a *N. tabacum-*like phospholipid monolayer, filled with *N. benthamiana-*like WEs; Supplementary Videos S53 to S58, MDS of AtLDAP1 with a jojoba like TAG-filled monolayer; Supplementary Videos S59 to S64, MDS of ScLDAP1 with a jojoba like TAG-filled monolayer membrane; Supplementary Videos S65 to S70, MDS of AtLDAP1 with a *N. tabacum*-like TAG-filled monolayer; Supplementary Videos S71 to S76, MDS of ScLDAP1 with a *N. tabacum-*like TAG-filled monolayer.

### Computational analysis

Simulation data were analyzed using python-enabled VMD 1.9.4a48. (Humphrey et al. 1996). Python-enabled VMD allowed us to use the numerical libraries numpy (Van Der Walt et al. 2011) for analysis and matplotlib (Hunter 2007) for generating plots and figures. Interactions between LDAP1 protein and membrane were evaluated using python scripts. Protein-membrane contacts were calculated using a coordination number, first evaluating all protein-membrane heavy atom pairs within 5 Å and then summing the distance-weighted contact function according to the equation:


$$C_{ij}(t)=\sum_{i=1,j=1}^{N,M}\frac{1}{1+e^{\left(5(d_{ij}(t))-4)\right)}}$$

where *C_ij_* is the matrix of contact points between paired atoms, *i* and *j* where we calculate a distance *d_ij_*. The pairs are between N protein-heavy atoms and M lipid-heavy atoms. Although derived from a native contact definition (Sheinerman and Brooks 1998), this functional form has proved versatile for quantifying close contacts in several biological systems (Vermaas et al. 2015, 2018; Vermaas and Tajkhorshid 2017; Zheng et al. 2019).

Membrane penetration depths of individual protein residues were quantified by calculating the probability densities of the *z*-distance in relation to the membrane. Since the membrane composition is symmetric, and the protein can bind to either side of the membrane due to the periodic boundary conditions of the simulation system, we measured the absolute value of the distance. Spatial gaps in the lipid monolayer were calculated using PackMem (Gautier et al. 2018).

### Statistical analysis

Statistical analysis of total CFP fluorescence associated with ER defects induced by WE accumulation was all performed using the Kruskal–Wallis test followed by Dunn's post-test using PRISM (v10.2.3) (GraphPad; www.graphpad.com). Statistical analysis of WE and TAG quantification from both total lipids, and isolated cellular fractions, was all performed using either Kruskal–Wallis test followed by Dunn's post-test or ordinary 1-way ANOVA followed by Tukey's post hoc multiple comparison tests using PRISM (v10.2.3) (GraphPad; www.graphpad.com). Statistical analysis of the seed percent germination assay was performed using ordinary 1-way ANOVA followed by Tukey's post hoc multiple comparison test using PRISM (v10.2.3) (GraphPad; www.graphpad.com). Summaries of all statistical analysis data are available in Supplementary Table S3.

### Accession numbers

Accession numbers, taken from The Arabidopsis Information Resource (https://www.Arabidopsis.org/), National Center for Biotechnology Information (https://www.ncbi.nlm.nih.gov/), Sol Genomics Network (https://solgenomics.net/), and the National Genomics Data Center, Chinese National Genomics Data Center for Bioinformation (https://ngdc.cncb.ac.cn). Accession numbers are as follows: AtLDAP1 (At1g67360), AtLDAP2 (AT2G47780), AtLDAP3 (AT3G05500), NbLDIP (Niben101Scf06413g00005.1), EF1α (Niben101Scf08618g01012.1) TBSV P19 (AJ288924.1), TYLCV V2 (NC_003828.1), Mm-FIT2 (NM_173397), ScWS (Sc13g0002650.01), ScFAR (Sc12g0004030.01), ScFAE (Sc13g0004890.01), ScLDIP (Sc11g0004790.01), ScLDAP1 (Sc04g0010120.01), ScLDAP3 (Sc16g0008650.01), ScSeipin1 (Sc16g0010380.01), ScSeipin2 (Sc17g0005550.01), ScOleosin1 (Sc22g0000890.01), ScOleosin5 (Sc03g0007530.01), ScOleosin5-like (Sc16g0003220.01), NbLDAP1 (Niben101Scf05621g05004.1), NbLDAP2, (Niben101Scf07086g00016.1), NbLDAP3 (Niben101Scf13703g00003.1).

## Supplementary Material

koaf115_Supplementary_Data

## Data Availability

The molecular simulation inputs and selected outputs, as well as the analysis behind the plots, are available through Zenodo: https://doi.org/10.5281/zenodo.11123374.

## Supplementary data

The following materials are available in the online version of this article.

**Supplementary Figure S1.** WE-induced BODIPY-stained defects form independent of CFP-HDEL expression.

**Supplementary Figure S2.** WE Molecular species composition from infiltrated *N. benthamiana* leaves.

**Supplementary Figure S3.** Transient expression of *N. benthamiana* LDAP1 does not correct WE-induced defects.

**Supplementary Figure S4.** Transient expression of LDAP isoforms from Arabidopsis and *N. benthamiana* do not correct WE-induced defects.

**Supplementary Figure S5.** Analysis of TAG distribution between isolated LDs and microsomes from infiltrated *N. benthamiana* leaves.

**Supplementary Figure S6.** Confirmation of *NbLDIP* silencing in *N. benthamiana* leaves by qualitative RT-PCR.

**Supplementary Figure S7.** Emergence of WE over the LD membrane surface.

**Supplementary Figure S8.** Overhead view of the membrane compositions of the head groups in jojoba and *N. tabacum*-like systems.

**Supplementary Figure S9.** Conformation of WEs in jojoba and *N. tabacum*-like membrane systems.

**Supplementary Figure S10.** Insertion of a WE layer inside a membrane bilayer to mimic the LD monolayer.

**Supplementary Figure S11.** Time-based contact plots of Arabidopsis and ScLDAP1 with bilayer membranes and LD monolayers with WEs.

**Supplementary Figure S12.** Simulated interactions of AtLDAP1 and ScLDAP1 with a *N. tabacum*-like bilayer, WE- and TAG-filled monolayer.

**Supplementary Figure S13.** Average area of phospholipid membrane defects observed during a bilayer, and WE-filled or TAG-filled monolayer simulation.

**Supplementary Figure S14.** ScLDAP1 expression does not affect WE profiles in Arabidopsis seeds expressing WE machinery.

**Supplementary Figure S15.** AlphaFold2 modeling, and sequence alignment of AtLDAP1 and ScLDAP1.

**Supplementary Figure S16.** Confirmation of transgene expression by qualitative RT-PCR.

**Supplementary Figure S17.** Thickness of membranes with WEs measured by the phosphate-to-phosphate distance from the 2 monolayers.

**Supplementary Figure S18.** Depiction of the simulation box along with the 6-replica initial protein starting position.

**Supplementary Table S1.** Lipid composition of phospholipid membranes, and WE and TAG cores used in MDS systems.

**Supplementary Table S2.** Oligonucleotide sequences used for plasmid construction and RT-PCR.

**Supplementary Table S3.** Reports from the statistical tests performed in GraphPad Prism.

**Supplementary Video S1.** MDS of a jojoba-like WE-filled monolayer.

**Supplementary Video S2.** MDS of an *N. tabacum-*like phospholipid monolayer filled with *N. benthamiana-*like WEs.

**Supplementary Videos S3 to S8.** MDS of AtLDAP1 with a jojoba-like bilayer.

**Supplementary Videos S9 to S14.** MDS of ScLDAP1 with a jojoba-like bilayer.

**Supplementary Videos S15 to S20.** MDS of AtLDAP1 with a jojoba-like WE-filled monolayer.

**Supplementary Videos S21 to S26.** MDS of ScLDAP1 with a jojoba-like WE-filled monolayer.

**Supplementary Videos S27 to S32.** MDS of AtLDAP1 with an *N. tabacum*-like bilayer.

**Supplementary Videos S33 to S38.** MDS of ScLDAP1 with an *N. tabacum*-like bilayer.

**Supplementary Videos S39 to S44.** MDS of AtLDAP1 with an *N. tabacum-*like phospholipid monolayer filled with *N. benthamiana-*like WEs.

**Supplementary Videos S45 to S50.** MDS of ScLDAP1 with an *N. tabacum-*like phospholipid monolayer filled with *N. benthamiana-*like WEs.

**Supplementary Video S51.** MDS of a jojoba-like TAG-filled monolayer.

**Supplementary Video S52.** MDS of an *N. tabacum*-like TAG-filled monolayer.

**Supplementary Videos S53 to S58.** MDS of AtLDAP1 with a jojoba-like TAG-filled monolayer.

**Supplementary Videos S59 to S64.** MDS of ScLDAP1 with a jojoba-like TAG-filled monolayer.

**Supplementary Videos S65 to S70.** MDS of AtLDAP1 with an *N. tabacum*-like TAG-filled monolayer.

**Supplementary Videos S71 to S76.** MDS of ScLDAP1 with an *N. tabacum-*like TAG-filled monolayer.

## Dive Curated Terms

The following phenotypic, genotypic, and functional terms are of significance to the work described in this paper:

SDP1 Gramene: AT5G04040
SDP1 Araport: AT5G04040

lipid CHEBI: CHEBI:18059

PUX10 Gramene: AT4G10790
PUX10 Araport: AT4G10790

CLSM Gramene: Confocal Laser-Scanning Microscopy
CLSM Araport: Confocal Laser-Scanning Microscopy

## References

[koaf115-B1] Al Sulaimi R, Macknojia A, Eskandari M, Shirani A, Gautam B, Park W, Whitehead P, Alonso AP, Sedbrook JC, Chapman KD. Evaluating the effects of very long chain and hydroxy fatty acid content on tribological performance and thermal oxidation behavior of plant-based lubricants. Tribol Int. 2023:185:108576.

[koaf115-B2] Antoine G, Vaissayre V, Meile J-C, Payet J, Conéjéro G, Costet L, Fock-Bastide I, Joët T, Dussert S. Diterpenes of coffea seeds show antifungal and anti-insect activities and are transferred from the endosperm to the seedling after germination. Plant Physiol Biochem. 2023:194:627–637. 10.1016/j.plaphy.2022.12.013 36535102

[koaf115-B3] Bartels D, Dörmann P. Plant lipids: methods and protocols. New York (NY): Humana Press; 2021. Chapter 16.

[koaf115-B4] Birsoy K, Festuccia WT, Laplante M. A comparative perspective on lipid storage in animals. J Cell Sci. 2013:126(7):1541–1552. 10.1242/jcs.104992 23658371

[koaf115-B5] Bouchnak I, Coulon D, Salis V, D’Andréa S, Brehelin C. Lipid droplets are versatile organelles involved in plant development and plant response to environmental changes. Front Plant Sci. 2023:14:1193905. 10.3389/fpls.2023.1193905 37426978 PMC10327486

[koaf115-B6] Braun RJ, Swanson JMJ. Capturing the liquid-crystalline phase transformation: implications for protein targeting to sterol ester-rich lipid droplets. Membranes (Basel). 2022:12(10):949. 10.3390/membranes12100949 36295707 PMC9607156

[koaf115-B7] Brooks BR, Bruccoleri RE, Olafson BD, States DJ, Swaminathan SA, Karplus M. CHARMM: a program for macromolecular energy, minimization, and dynamics calculations. J Comput Chem. 1983:4(2):187–217. 10.1002/jcc.540040211

[koaf115-B8] Cai Y, Goodman JM, Pyc M, Mullen RT, Dyer JM, Chapman KD. Arabidopsis SEIPIN proteins modulate triacylglycerol accumulation and influence lipid droplet proliferation. Plant Cell. 2015:27(9):2616–2636. 10.1105/tpc.15.00588 26362606 PMC4815042

[koaf115-B9] Cartwright BR, Goodman JM. Seipin: from human disease to molecular mechanism. J Lipid Res. 2012:53(6):1042–1055. 10.1194/jlr.R023754 22474068 PMC3351812

[koaf115-B10] Chapman KD, Aziz M, Dyer JM, Mullen RT. Mechanisms of lipid droplet biogenesis. Biochem J. 2019:476(13):1929–1942. 10.1042/BCJ20180021 31289128

[koaf115-B11] Chen JCF, Tsai CCY, Tzen JTC. Cloning and secondary structure analysis of caleosin, a unique calcium-binding protein in oil bodies of plant seeds. Plant Cell Physiol. 1999:40(10):1079–1086. 10.1093/oxfordjournals.pcp.a029490 10589521

[koaf115-B12] Chorlay A, Thiam AR. Neutral lipids regulate amphipathic helix affinity for model lipid droplets. J Cell Biol. 2020:219(4):e201907099. 10.1083/jcb.201907099 32328636 PMC7147095

[koaf115-B13] Choudhary V, Schneiter R. Lipid droplet biogenesis from specialized ER subdomains. Microbial Cell. 2020:7(8):218. 10.15698/mic2020.08.727 32743002 PMC7380455

[koaf115-B14] Chung J, Wu X, Lambert TJ, Lai ZW, Walther TC, Farese RV. LDAF1 and seipin form a lipid droplet assembly complex. Dev Cell. 2019:51(5):551–563.e7. 10.1016/j.devcel.2019.10.006 31708432 PMC7235935

[koaf115-B15] Clews AC, Ulch BA, Jesionowska M, Hong J, Mullen RT, Xu Y. Variety of plant oils: species-specific lipid biosynthesis. Plant Cell Physiol. 2023:pcad147. 10.1093/pcp/pcad147 37971406

[koaf115-B16] Clough SJ, Bent AF. Floral dip: a simplified method for Agrobacterium-mediated transformation of Arabidopsis thaliana. Plant J. 1998:16(6):735–743. 10.1046/j.1365-313x.1998.00343.x 10069079

[koaf115-B17] Čopič A, Antoine-Bally S, Giménez-Andrés M, La Torre Garay C, Antonny B, Manni MM, Pagnotta S, Guihot J, Jackson CL. A giant amphipathic helix from a perilipin that is adapted for coating lipid droplets. Nat Commun. 2018:9(1):1332. 10.1038/s41467-018-03717-8 29626194 PMC5889406

[koaf115-B18] Curtis MD, Grossniklaus U. A gateway cloning vector set for high-throughput functional analysis of genes in planta. Plant Physiol. 2003:133(2):462–469. 10.1104/pp.103.027979 14555774 PMC523872

[koaf115-B19] Darden T, York D, Pedersen L. Particle mesh Ewald: an N⋅log (N) method for Ewald sums in large systems. J Chem Phys. 1993:98(12):10089–10092. 10.1063/1.464397

[koaf115-B20] Dean EA, Finer JJ. Amino acids induce high seed-specific expression driven by a soybean (*Glycine max*) glycinin seed storage protein promoter. Plant Cell Rep. 2023:42(1):123–136. 10.1007/s00299-022-02940-4 36271177

[koaf115-B21] Deruyffelaere C, Purkrtova Z, Bouchez I, Collet B, Cacas J-L, Chardot T, Gallois J-L, D’Andrea S. PUX10 is a CDC48A adaptor protein that regulates the extraction of ubiquitinated oleosins from seed lipid droplets in Arabidopsis. Plant Cell. 2018:30(9):2116–2136. 10.1105/tpc.18.00275 30087208 PMC6181022

[koaf115-B22] de Vries J, Ischebeck T. Ties between stress and lipid droplets pre-date seeds. Trends Plant Sci. 2020:25(12):1203–1214. 10.1016/j.tplants.2020.07.017 32921563

[koaf115-B23] Dhiman R, Caesar S, Thiam AR, Schrul B. Mechanisms of protein targeting to lipid droplets: a unified cell biological and biophysical perspective. Semin Cell Dev Biol. 2020:108:4–13. 10.1016/j.semcdb.2020.03.004 32201131

[koaf115-B24] Domergue F, Miklaszewska M. The production of wax esters in transgenic plants: towards a sustainable source of bio-lubricants. J Exp Bot. 2022:73(9):2817–2834. 10.1093/jxb/erac046 35560197 PMC9113324

[koaf115-B25] Essmann U, Perera L, Berkowitz ML, Darden T, Lee H, Pedersen LG. A smooth particle mesh Ewald method. J Chem Phys. 1995:103(19):8577–8593. 10.1063/1.470117

[koaf115-B26] Feller SE, Zhang Y, Pastor RW, Brooks BR. Constant pressure molecular dynamics simulation: the Langevin piston method. J Chem Phys. 1995:103(11):4613–4621. 10.1063/1.470648

[koaf115-B27] Gao M, Huang X, Song B-L, Yang H. The biogenesis of lipid droplets: lipids take center stage. Prog Lipid Res. 2019:75:100989. 10.1016/j.plipres.2019.100989 31351098

[koaf115-B28] Gautier R, Bacle A, Tiberti ML, Fuchs PF, Vanni S, Antonny B. PackMem: a versatile tool to compute and visualize interfacial packing defects in lipid bilayers. Biophys J. 2018:115(3):436–444. 10.1016/j.bpj.2018.06.025 30055754 PMC6084522

[koaf115-B29] Gidda SK, Park S, Pyc M, Yurchenko O, Cai Y, Wu P, Andrews DW, Chapman KD, Dyer JM, Mullen RT. Lipid droplet-associated proteins (LDAPs) are required for the dynamic regulation of neutral lipid compartmentation in plant cells. Plant Physiol. 2016:170(4):2052–2071. 10.1104/pp.15.01977 26896396 PMC4825156

[koaf115-B30] Gidda SK, Watt SC, Collins-Silva J, Kilaru A, Arondel V, Yurchenko O, Horn PJ, James CN, Shintani D, Ohlrogge JB. Lipid droplet-associated proteins (LDAPs) are involved in the compartmentalization of lipophilic compounds in plant cells. Plant Signal Behav. 2013:8(11):e27141. 10.4161/psb.27141 24305619 PMC4091607

[koaf115-B31] Giménez-Andrés M, Čopič A, Antonny B. The many faces of amphipathic helices. Biomolecules. 2018:8(3):45. 10.3390/biom8030045 29976879 PMC6164224

[koaf115-B32] Gülz P-G, Eich C. Composition of phospholipids in seed oil of jojoba (*Simmondsia chinensis* [link], Schneider). Z Naturforsch C. 1982:37(11–12):1286–1287. 10.1515/znc-1982-11-1232

[koaf115-B33] Guzha A, Whitehead P, Ischebeck T, Chapman KD. Lipid droplets: packing hydrophobic molecules within the aqueous cytoplasm. Annu Rev Plant Biol. 2023:74(1):195–223. 10.1146/annurev-arplant-070122-021752 36413579

[koaf115-B34] Hayes DG, Kleiman R, Phillips BS. The triglyceride composition, structure, and presence of estolides in the oils of*Lesquerella* and related species. J Am Oil Chem Soc. 1995:72(5):559–569.

[koaf115-B35] Heilmann M, Iven T, Ahmann K, Hornung E, Stymne S, Feussner I. Production of wax esters in plant seed oils by oleosomal cotargeting of biosynthetic enzymes. J Lipid Res. 2012:53:2153–2161. 22878160 10.1194/jlr.M029512 PMC3435548

[koaf115-B36] Henne M, Goodman JM, Hariri H. Spatial compartmentalization of lipid droplet biogenesis. Biochim Biophys Acta Mol Cell Biol Lipids. 2020:1865(1):158499. 10.1016/j.bbalip.2019.07.008 31352131 PMC7050823

[koaf115-B37] Hilgarth RS, Lanigan TM. Optimization of overlap extension PCR for efficient transgene construction. MethodsX. 2020:7:100759. 10.1016/j.mex.2019.12.001 32021819 PMC6992990

[koaf115-B38] Horn PJ, James CN, Gidda SK, Kilaru A, Dyer JM, Mullen RT, Ohlrogge JB, Chapman KD. Identification of a new class of lipid droplet-associated proteins in plants. Plant Physiol. 2013:162(4):1926–1936. 10.1104/pp.113.222455 23821652 PMC3729771

[koaf115-B39] Huang AHC . Plant lipid droplets and their associated proteins: potential for rapid advances. Plant Physiol. 2018:176(3):1894–1918. 10.1104/pp.17.01677 29269574 PMC5841732

[koaf115-B40] Huang C-Y, Huang AHC. Unique motifs and length of hairpin in oleosin target the cytosolic side of endoplasmic reticulum and budding lipid droplet. Plant Physiol. 2017:174(4):2248–2260. 10.1104/pp.17.00366 28611060 PMC5543949

[koaf115-B41] Huang J, Rauscher S, Nawrocki G, Ran T, Feig M, De Groot BL, Grubmüller H, MacKerell AD Jr. CHARMM36m: an improved force field for folded and intrinsically disordered proteins. Nat Methods. 2017:14(1):71–73. 10.1038/nmeth.4067 27819658 PMC5199616

[koaf115-B42] Humphrey W, Dalke A, Schulten K. VMD: visual molecular dynamics. J Mol Graph. 1996:14(1):33–38. 10.1016/0263-7855(96)00018-5 8744570

[koaf115-B43] Hunter JD . Matplotlib: a 2D graphics environment. Comput Sci Eng. 2007:9(3):90–95. 10.1109/MCSE.2007.55

[koaf115-B44] Ischebeck T, Krawczyk HE, Mullen RT, Dyer JM, Chapman KD. Lipid droplets in plants and algae: distribution, formation, turnover and function. Semin Cell Dev Biol. 2020:108:82–93. 10.1016/j.semcdb.2020.02.014 32147380

[koaf115-B45] Ishida N . A comprehensive study on triacylglycerols in tobacco leaves using liquid chromatography and atmospheric-pressure chemical-ionization mass spectrometry. Contrib Tob Nicotine Res. 2013:25(7):627–637. 10.2478/cttr-2013-0939

[koaf115-B46] Ivarson E, Iven T, Sturtevant D, Ahlman A, Cai Y, Chapman K, Feussner I, Zhu L-H. Production of wax esters in the wild oil species Lepidium campestre. Ind Crops Prod. 2017:108:535–542. 10.1016/j.indcrop.2017.07.002

[koaf115-B47] Iven T, Herrfurth C, Hornung E, Heilmann M, Hofvander P, Stymne S, Zhu L-H, Feussner I. Wax ester profiling of seed oil by nano-electrospray ionization tandem mass spectrometry. Plant Methods. 2013:9(1):24. 10.1186/1746-4811-9-24 23829499 PMC3766222

[koaf115-B48] Iven T, Hornung E, Heilmann M, Feussner I. Synthesis of oleyl oleate wax esters in Arabidopsis thaliana and Camelina sativa seed oil. Plant Biotechnol J. 2016:14(1):252–259. 10.1111/pbi.12379 25912558 PMC11389091

[koaf115-B49] Jackson CL . Lipid droplet biogenesis. Curr Opin Cell Biol. 2019:59:88–96. 10.1016/j.ceb.2019.03.018 31075519

[koaf115-B50] Jo S, Kim T, Iyer VG, Im W. CHARMM-GUI: a web-based graphical user interface for CHARMM. J Comput Chem. 2008:29(11):1859–1865. 10.1002/jcc.20945 18351591

[koaf115-B51] Jumper J, Evans R, Pritzel A, Green T, Figurnov M, Ronneberger O, Tunyasuvunakool K, Bates R, Žídek A, Potapenko A. Highly accurate protein structure prediction with AlphaFold. Nature. 2021:596(7873):583–589. 10.1038/s41586-021-03819-2 34265844 PMC8371605

[koaf115-B52] Kim S, Swanson JMJ, Voth GA. Computational studies of lipid droplets. J Phys Chem B. 2022:126(11):2145–2154. 10.1021/acs.jpcb.2c00292 35263109 PMC8957551

[koaf115-B53] Kohlmeyer A, Vermaas J. TopoTools. Version v1.7, Zonodo.org 2017.

[koaf115-B54] Krawczyk HE, Sun S, Doner NM, Yan Q, Lim MSS, Scholz P, Niemeyer PW, Schmitt K, Valerius O, Pleskot R. SEED LIPID DROPLET PROTEIN1, SEED LIPID DROPLET PROTEIN2, and LIPID DROPLET PLASMA MEMBRANE ADAPTOR mediate lipid droplet–plasma membrane tethering. Plant Cell. 2022:34(6):2424–2448. 10.1093/plcell/koac095 35348751 PMC9134073

[koaf115-B55] Kulke M, Kurtz E, Boren DM, Olson DM, Koenig AM, Hoffmann-Benning S, Vermaas JV. PLAT domain protein 1 (PLAT1/PLAFP) binds to the *Arabidopsis thaliana* plasma membrane and inserts a lipid. Plant Sci. 2024:338:111900. 10.1016/j.plantsci.2023.111900 37863269

[koaf115-B56] Kumari RM, Khatri A, Chaudhary R, Choudhary V. Concept of lipid droplet biogenesis. Eur J Cell Biol. 2023:102(4):151362. 10.1016/j.ejcb.2023.151362 37742390 PMC7615795

[koaf115-B57] Laibach N, Schmidl S, Müller B, Bergmann M, Prüfer D, Schulze Gronover C. Small rubber particle proteins from *Taraxacum brevicorniculatum* promote stress tolerance and influence the size and distribution of lipid droplets and artificial poly (cis-1, 4-isoprene) bodies. Plant J. 2018:93(6):1045–1061. 10.1111/tpj.13829 29377321

[koaf115-B58] Lanier ER, Andersen TB, Hamberger B. Plant terpene specialized metabolism: complex networks or simple linear pathways? Plant J. 2023:114(5):1178–1201. 36891828 10.1111/tpj.16177 PMC11166267

[koaf115-B59] Lin L-J, Tai SSK, Peng C-C, Tzen JTC. Steroleosin, a sterol-binding dehydrogenase in seed oil bodies. Plant Physiol. 2002:128(4):1200–1211. 10.1104/pp.010982 11950969 PMC154248

[koaf115-B60] Lindsey BE 3rd, Rivero L, Calhoun CS, Grotewold E, Brkljacic J. Standardized method for high-throughput sterilization of arabidopsis seeds. J Vis Exp. 2017(128):56587. 10.3791/56587 29155739 PMC5752416

[koaf115-B61] Lundquist PK, Shivaiah K-K, Espinoza-Corral R. Lipid droplets throughout the evolutionary tree. Prog Lipid Res. 2020:78:101029. 10.1016/j.plipres.2020.101029 32348789

[koaf115-B62] Miklaszewska M, Banaś A. Biochemical characterization and substrate specificity of jojoba fatty acyl-CoA reductase and jojoba wax synthase. Plant Sci. 2016:249:84–92. 10.1016/j.plantsci.2016.05.009 27297992

[koaf115-B63] Miquel M, Trigui G, d’Andréa S, Kelemen Z, Baud S, Berger A, Deruyffelaere C, Trubuil A, Lepiniec L, Dubreucq B. Specialization of oleosins in oil body dynamics during seed development in Arabidopsis seeds. Plant Physiol. 2014:164(4):1866–1878. 10.1104/pp.113.233262 24515832 PMC3982749

[koaf115-B64] Miyamoto S, Kollman PA. Settle: an analytical version of the SHAKE and RATTLE algorithm for rigid water models. J Comput Chem. 1992:13(8):952–962. 10.1002/jcc.540130805

[koaf115-B65] Naim F, Nakasugi K, Crowhurst RN, Hilario E, Zwart AB, Hellens RP, Taylor JM, Waterhouse PM, Wood CC. Advanced engineering of lipid metabolism in *Nicotiana benthamiana* using a draft genome and the V2 viral silencing-suppressor protein. PLoS One. 2012:7(12):e52717. 10.1371/journal.pone.0052717 23300750 PMC3530501

[koaf115-B66] Nettebrock NT, Bohnert M. Born this way–biogenesis of lipid droplets from specialized ER subdomains. Biochim Biophys Acta Mol Cell Biol Lipids. 2020:1865(1):158448. 10.1016/j.bbalip.2019.04.008 31028912

[koaf115-B67] Ohlrogge J, Thrower N, Mhaske V, Stymne S, Baxter M, Yang W, Liu J, Shaw K, Shorrosh B, Zhang M. PlantFAdb: a resource for exploring hundreds of plant fatty acid structures synthesized by thousands of plants and their phylogenetic relationships. Plant J. 2018:96(6):1299–1308. 10.1111/tpj.14102 30242919

[koaf115-B68] Olsson MH, Søndergaard CR, Rostkowski M, Jensen JH. PROPKA3: consistent treatment of internal and surface residues in empirical pKa predictions. J Chem Theory Comput. 2011:7(2):525–537. 10.1021/ct100578z 26596171

[koaf115-B69] Olzmann JA, Carvalho P. Dynamics and functions of lipid droplets. Nat Rev Mol Cell Biol. 2019:20(3):137–155. 10.1038/s41580-018-0085-z 30523332 PMC6746329

[koaf115-B70] Park JW, Kim CH, Kang H-G, Do Choi Y. Promoter sequence of soybean glycinin gene regulates seed-specific expression in transgenic tobacco plant. Mol Cells. 1992:2(3):297–302. 10.1016/S1016-8478(23)13944-6

[koaf115-B71] Petrie JR, Shrestha P, Liu Q, Mansour MP, Wood CC, Zhou X-R, Nichols PD, Green AG, Singh SP. Rapid expression of transgenes driven by seed-specific constructs in leaf tissue: DHA production. Plant Methods. 2010:6(1):8. 10.1186/1746-4811-6-8 20222981 PMC2845569

[koaf115-B72] Phillips JC, Hardy DJ, Maia JDC, Stone JE, Ribeiro JV, Bernardi RC, Buch R, Fiorin G, Hénin J, Jiang W. Scalable molecular dynamics on CPU and GPU architectures with NAMD. J Chem Phys. 2020:153(4):044130. 10.1063/5.0014475 32752662 PMC7395834

[koaf115-B73] Prévost C, Sharp ME, Kory N, Lin Q, Voth GA, Farese RV Jr, Walther TC. Mechanism and determinants of amphipathic helix-containing protein targeting to lipid droplets. Dev Cell. 2018:44(1):73–86.e4. 10.1016/j.devcel.2017.12.011 29316443 PMC5764114

[koaf115-B74] Pyc M, Cai Y, Gidda SK, Yurchenko O, Park S, Kretzschmar FK, Ischebeck T, Valerius O, Braus GH, Chapman KD. Arabidopsis lipid droplet-associated protein (LDAP)–interacting protein (LDIP) influences lipid droplet size and neutral lipid homeostasis in both leaves and seeds. Plant J. 2017a:92(6):1182–1201. 10.1111/tpj.13754 29083105

[koaf115-B75] Pyc M, Cai Y, Greer MS, Yurchenko O, Chapman KD, Dyer JM, Mullen RT. Turning over a new leaf in lipid droplet biology. Trends Plant Sci. 2017b:22(7):596–609. 10.1016/j.tplants.2017.03.012 28454678

[koaf115-B76] Pyc M, Gidda SK, Seay D, Esnay N, Kretzschmar FK, Cai Y, Doner NM, Greer MS, Hull JJ, Coulon D. LDIP cooperates with SEIPIN and LDAP to facilitate lipid droplet biogenesis in Arabidopsis. Plant Cell. 2021:33(9):3076–3103. 10.1093/plcell/koab179 34244767 PMC8462815

[koaf115-B77] Qiu B, Simon MC. BODIPY 493/503 staining of neutral lipid droplets for microscopy and quantification by flow cytometry. Bio-Protocol. 2016:6(17):e1912–e1912. 28573161 10.21769/BioProtoc.1912 PMC5448404

[koaf115-B78] Rahman F, Hassan M, Rosli R, Almousally I, Hanano A, Murphy DJ. Evolutionary and genomic analysis of the caleosin/peroxygenase (CLO/PXG) gene/protein families in the Viridiplantae. PLoS One. 2018:13(5):e0196669. 10.1371/journal.pone.0196669 29771926 PMC5957377

[koaf115-B79] Ramachandran GN, Sasisekharan V. Conformation of polypeptides and proteins. Adv Protein Chem. 1968:23:283–438. 10.1016/S0065-3233(08)60402-7 4882249

[koaf115-B80] Rask L, Ellerström M, Ezcurra I, Stålberg K, Wycliffe P. Seed-specific regulation of the napin promoter in *Brassica napus*. J Plant Physiol. 1998:152(6):595 599. 10.1016/S0176-1617(98)80017-5

[koaf115-B81] Renne MF, Klug YA, Carvalho P. Lipid droplet biogenesis: a mystery “unmixing”? Semin Cell Dev Biol. 2020:108:14–23. 10.1016/j.semcdb.2020.03.001 32192830

[koaf115-B82] Romsdahl T, Shirani A, Minto RE, Zhang C, Cahoon EB, Chapman KD, Berman D. Nature-guided synthesis of advanced bio-lubricants. Sci Rep. 2019:9(1):11711. 31406215 10.1038/s41598-019-48165-6 PMC6690888

[koaf115-B83] Ruzicska P, Gombos Z, Farkas GL. Modification of the fatty acid composition of phospholipids during the hypersensitive reaction in tobacco. Virology. 1983:128(1):60–64. 10.1016/0042-6822(83)90318-5 18639025

[koaf115-B84] Sadre R, Kuo P, Chen J, Yang Y, Banerjee A, Benning C, Hamberger B. Cytosolic lipid droplets as engineered organelles for production and accumulation of terpenoid biomaterials in leaves. Nat Commun. 2019:10(1):853. 10.1038/s41467-019-08515-4 30787273 PMC6382807

[koaf115-B85] Santinho A, Salo VT, Chorlay A, Li S, Zhou X, Omrane M, Ikonen E, Thiam AR. Membrane curvature catalyzes lipid droplet assembly. Curr Biol. 2020:30(13):2481–2494. 10.1016/j.cub.2020.04.066 32442467

[koaf115-B86] Sapia J, Vanni S. Molecular dynamics simulations of intracellular lipid droplets: a new tool in the toolbox. FEBS Lett. 2024:598(10):1143–1153. 10.1002/1873-3468.14879 38627196

[koaf115-B87] Schindelin J, Arganda-Carreras I, Frise E, Kaynig V, Longair M, Pietzsch T, Preibisch S, Rueden C, Saalfeld S, Schmid B. Fiji: an open-source platform for biological-image analysis. Nat Methods. 2012:9(7):676–682. 10.1038/nmeth.2019 22743772 PMC3855844

[koaf115-B88] Scholz P, Chapman KD, Mullen RT, Ischebeck T. Finding new friends and revisiting old ones–how plant lipid droplets connect with other subcellular structures. New Phytol. 2022:236(3):833–838. 10.1111/nph.18390 35851478

[koaf115-B89] Sheinerman FB, Brooks CL III. Molecular picture of folding of a small α/β protein. Proc Natl Acad Sci U S A. 1998:95(4):1562–1567. 10.1073/pnas.95.4.1562 9465055 PMC19093

[koaf115-B90] Shimada TL, Hayashi M, Hara-Nishimura I. Membrane dynamics and multiple functions of oil bodies in seeds and leaves. Plant Physiol. 2018:176(1):199–207. 10.1104/pp.17.01522 29203559 PMC5761825

[koaf115-B91] Shivaiah K-K, Boren DM, Herrera-Tequia A, Vermaas J, Lundquist PK. An amphipathic helix drives interaction of Fibrillins with plastoglobule lipid droplets. bioRxiv 559984. 10.1101/2023.09.28.559984 29 September 2023, preprint: not peer reviewed.

[koaf115-B92] Siloto RMP, Findlay K, Lopez-Villalobos A, Yeung EC, Nykiforuk CL, Moloney MM. The accumulation of oleosins determines the size of seed oilbodies in Arabidopsis. Plant Cell. 2006:18(8):1961–1974. 10.1105/tpc.106.041269 16877495 PMC1533971

[koaf115-B93] Smith MA, Zhang H, Forseille L, Purves RW. Characterization of novel triacylglycerol estolides from the seed oil of *Mallotus philippensis* and *Trewia nudiflora*. Lipids. 2013:48:75–85. 23054551 10.1007/s11745-012-3721-y

[koaf115-B94] Spangenburg EE, Pratt SJP, Wohlers LM, Lovering RM. Use of BODIPY (493/503) to visualize intramuscular lipid droplets in skeletal muscle. J Biomed Biotechnol. 2011:2011:598358. 10.1155/2011/598358 21960738 PMC3180081

[koaf115-B95] Sturtevant D, Lu S, Zhou Z-W, Shen Y, Wang S, Song J-M, Zhong J, Burks DJ, Yang Z-Q, Yang Q-Y. The genome of jojoba (*Simmondsia chinensis*): a taxonomically isolated species that directs wax ester accumulation in its seeds. Sci Adv. 2020:6(11):eaay3240. 10.1126/sciadv.aay3240 32195345 PMC7065883

[koaf115-B96] Szymanski KM, Binns D, Bartz R, Grishin NV, Li W-P, Agarwal AK, Garg A, Anderson RGW, Goodman JM. The lipodystrophy protein seipin is found at endoplasmic reticulum lipid droplet junctions and is important for droplet morphology. Proc Natl Acad Sci U S A. 2007:104(52):20890–20895. 10.1073/pnas.0704154104 18093937 PMC2409237

[koaf115-B97] Thiam AR, Beller M. The why, when and how of lipid droplet diversity. J Cell Sci. 2017:130(2):315–324. 10.1242/jcs.192021 28049719

[koaf115-B98] Thiam AR, Ikonen E. Lipid droplet nucleation. Trends Cell Biol. 2021:31(2):108–118. 10.1016/j.tcb.2020.11.006 33293168

[koaf115-B151] Traver MS, Bartel B (2023). The ubiquitin-protein ligase MIEL1 localizes to peroxisomes to promote seedling oleosin degradation and lipid droplet mobilization. Proc Natl Acad Sci. USA 2023:120(29):e2304870120. 10.1073/pnas.2304870120 37410814 PMC10629534

[koaf115-B99] Van Der Walt S, Colbert SC, Varoquaux G. The NumPy array: a structure for efficient numerical computation. Comput Sci Eng. 2011:13(2):22–30. 10.1109/MCSE.2011.37

[koaf115-B100] Vermaas JV, Petridis L, Qi X, Schulz R, Lindner B, Smith JC. Mechanism of lignin inhibition of enzymatic biomass deconstruction. Biotechnol Biofuels. 2015:8(1):217. 10.1186/s13068-015-0379-8 26697106 PMC4687093

[koaf115-B101] Vermaas JV, Rempe SB, Tajkhorshid E. Electrostatic lock in the transport cycle of the multidrug resistance transporter EmrE. Proc Natl Acad Sci U S A. 2018:115(32):E7502–E7511. 10.1073/pnas.1722399115 30026196 PMC6094130

[koaf115-B102] Vermaas JV, Tajkhorshid E. Differential membrane binding mechanics of synaptotagmin isoforms observed in atomic detail. Biochemistry. 2017:56(1):281–293. 10.1021/acs.biochem.6b00468 27997124 PMC5557660

[koaf115-B103] Vollheyde K, Yu D, Hornung E, Herrfurth C, Feussner I. The fifth WS/DGAT enzyme of the bacterium Marinobacter aquaeolei VT8. Lipids. 2020:55:479–494. 10.1002/lipd.12250 32434279

[koaf115-B104] Willis RM, Wahlen BD, Seefeldt LC, Barney BM. Characterization of a fatty acyl-CoA reductase from Marinobacter aquaeolei VT8: a bacterial enzyme catalyzing the reduction of fatty acyl-CoA to fatty alcohol. Biochemistry. 2011:50(48):10550–10558. 10.1021/bi2008646 22035211

[koaf115-B105] Wimley WC, White SH. Experimentally determined hydrophobicity scale for proteins at membrane interfaces. Nat Struct Biol. 1996:3(10):842–848. 10.1038/nsb1096-842 8836100

[koaf115-B106] Zhan X, Zhang Y-H, Chen D-F, Simonsen HT. Metabolic engineering of the moss Physcomitrella patens to produce the sesquiterpenoids patchoulol and α/β-santalene. Front Plant Sci. 2014:5:636. 10.3389/fpls.2014.00636 25477891 PMC4235272

[koaf115-B107] Zhao Y, Dong Q, Geng Y, Ma C, Shao Q. Dynamic regulation of lipid droplet biogenesis in plant cells and proteins involved in the process. Int J Mol Sci. 2023:24(8):7476. 10.3390/ijms24087476 37108639 PMC10138601

[koaf115-B108] Zheng F, Vermaas JV, Zheng J, Wang Y, Tu T, Wang X, Xie X, Yao B, Beckham GT, Luo H. Activity and thermostability of GH5 endoglucanase chimeras from mesophilic and thermophilic parents. Appl Environ Microbiol. 2019:85(5):e02079-18. 10.1128/AEM.02079-18 30552196 PMC6384118

