# Supplementary material for: The jojoba lipid droplet protein LDAP1 facilitates the packaging of wax esters into lipid droplets
Source: Plant Cell. 2025 Aug 1;37(8):koaf115. doi: 10.1093/plcell/koaf115 (PMC12341951; doi:10.1093/plcell/koaf115)
Supplement: koaf115_Supplementary_Data [file koaf115_supplementary_data.zip › Supplementary Figures with legends kdc.pdf]

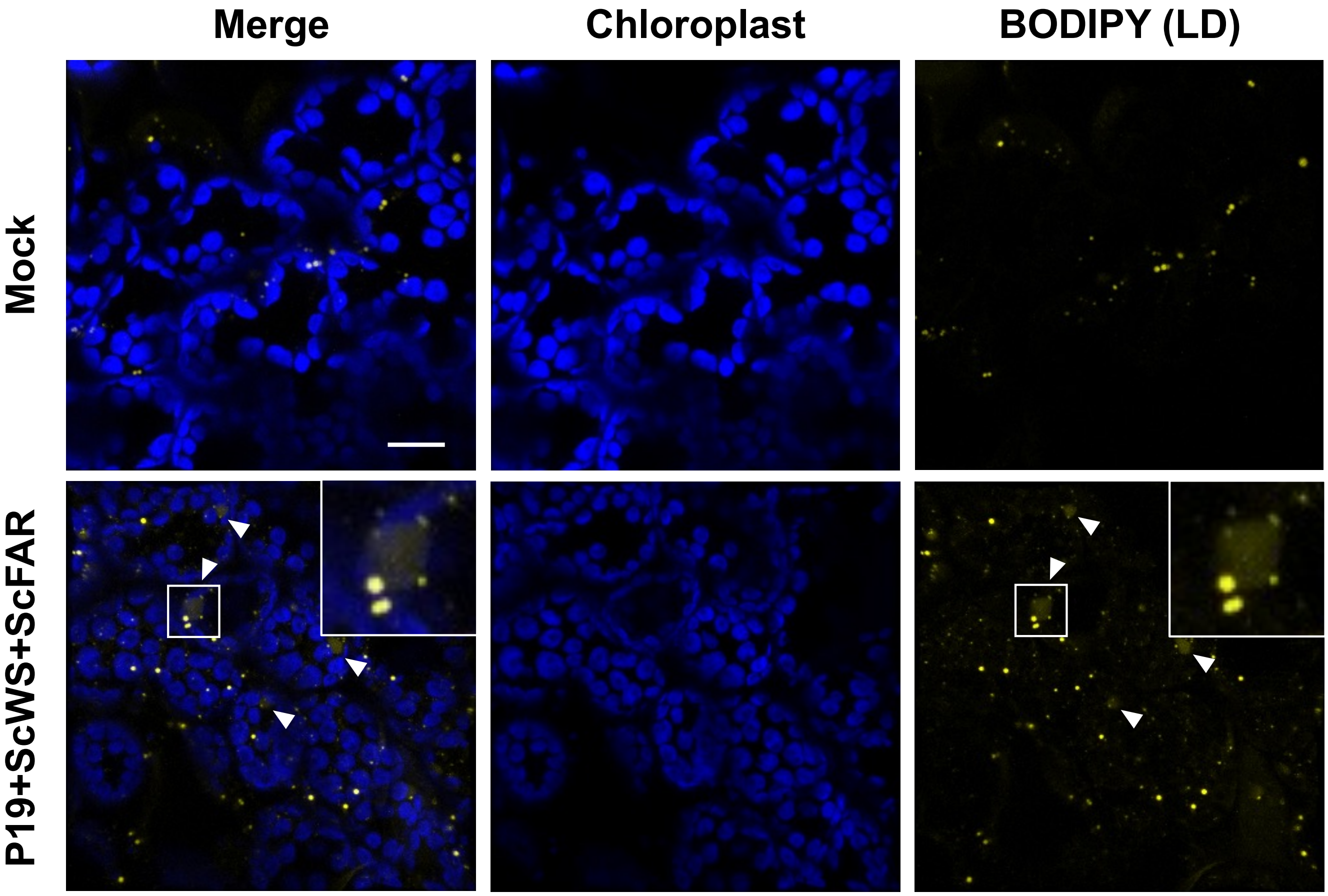

Supplementary Figure S1: WE- induced BODIPY- stained defects form independently of CFP-HDEL expression.

(Supports figure 1) Representative confocal laser scanning microscopy (CLSM) images of *N. benthamiana* leaves transiently expressing jojoba wax synthase (ScWS), and jojoba fatty acyl-CoA reductase (ScFAR). Samples were stained with BODIPY (493/503) to visualize neutral lipids. Arrows point to large BODIPY stained structures. Magnified inserts highlight BODIPY stained wax ester (WE) induced defects. For each transient expression experiment *A. tumefaciens* harboring the P19 viral suppressor construct was added to suppress transgene silencing. All samples were stained and imaged (Zeiss LSM710 confocal laser scanning microscope, fitted with AIRYSCAN attachment) at 4 days post infiltration. Chlorophyll autofluorescence, blue; BODIPY (493/503) yellow. Scale bar represent 20 µm for each original image, and 10 µm for all magnified inserts. The appearance of large BODIPY stained structures upon expression of ScWS and ScFAR support the hypothesis that endoplasmic reticulum (ER) defects and neutral lipid accumulation in the ER are a result of WE miss packaging and not protein aggregation. Abbreviation: lipid droplet (LD).

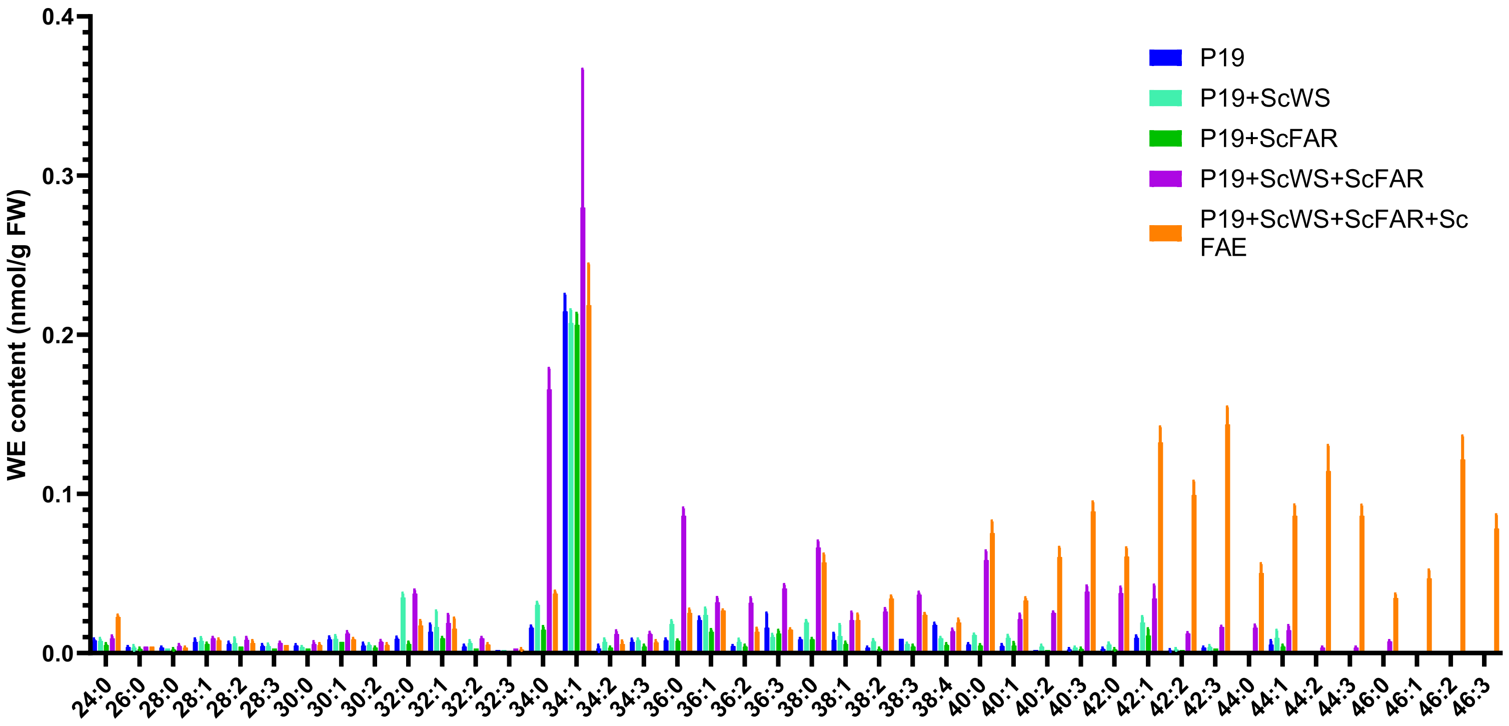

Supplementary Figure S2: WE molecular species composition from infiltrated *N. benthamiana* leaves.

(Supports figure 1) Molecular species of wax esters (WEs) in infiltrated *N. benthamiana* leaves producing jojoba wax synthase (ScWS) and jojoba fatty acyl-CoA reductase (ScFAR) individually, ScWS and ScFAR together, or ScWS and ScFAR with jojoba fatty acid elongase (ScFAE). Shown is the mean content (+ SD; n=3) of each identified WE species. Leaves co-producing ScWS, ScFar, and ScFAE produced longer chain (jojoba like) WEs when compared to leaves producing only ScWS and ScFAR. The x-axis labels correspond to the total number of carbons followed by total degrees of unsaturation (e.g., 32:1, 32 carbon WE with 1 degree of unsaturation). Abbreviation: fresh weight (FW).

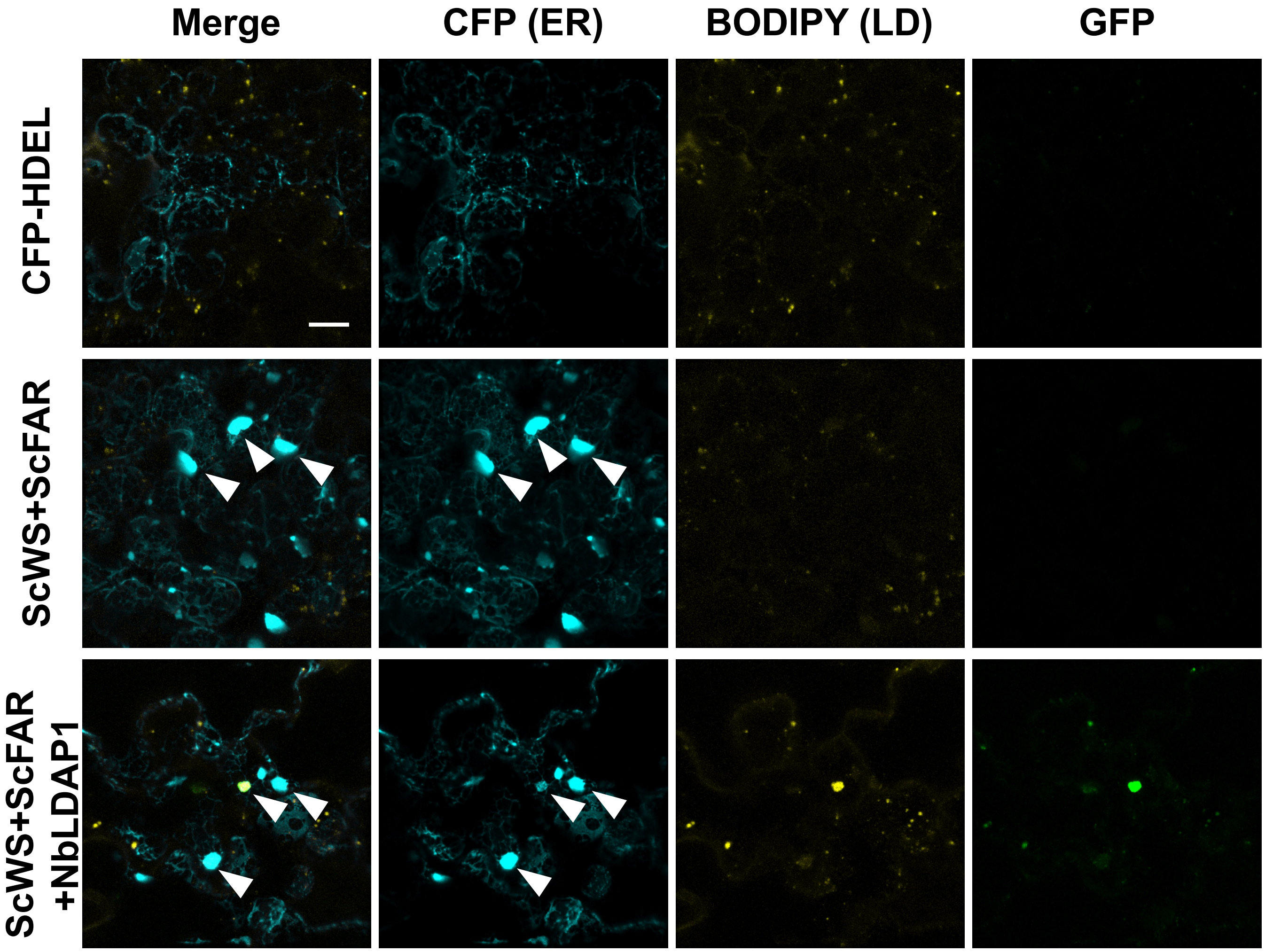

Supplementary Figure S3: Transient expression of *N. benthamiana* LDAP1 does not correct WE-induced defects.

(Supports figures 3) Representative confocal laser scanning microscopy (CLSM) images of *N. benthamiana* leaves transiently expressing jojoba wax synthase (ScWS), jojoba fatty acyl-CoA reductase (ScFAR), a cyan fluorescent protein (CFP) tagged endoplasmic reticulum (ER) marker (CFP-HDEL), and N-terminal green fluorescent protein (GFP) tagged *N. benthamiana* lipid droplet associated protein 1 (LDAP1) (NbLDAP1). Samples were stained with BODIPY (493/503) to visualize neutral lipids. Arrows point to regions of ER defects. For each transient expression experiment *A. tumefaciens* harboring the P19 viral suppressor construct was added to suppress transgene silencing. All samples were stained and imaged (Zeiss LSM710 confocal laser scanning microscope, fitted with AIRYSCAN attachment) at 4 days post infiltration. Scale bar represent 20 μm for each image. Transient production of *N. benthamiana* NbLDAP1 in conjunction with WE accumulation showed no restoration of wax ester (WE)-induced defects. Abbreviation: lipid droplet (LD).

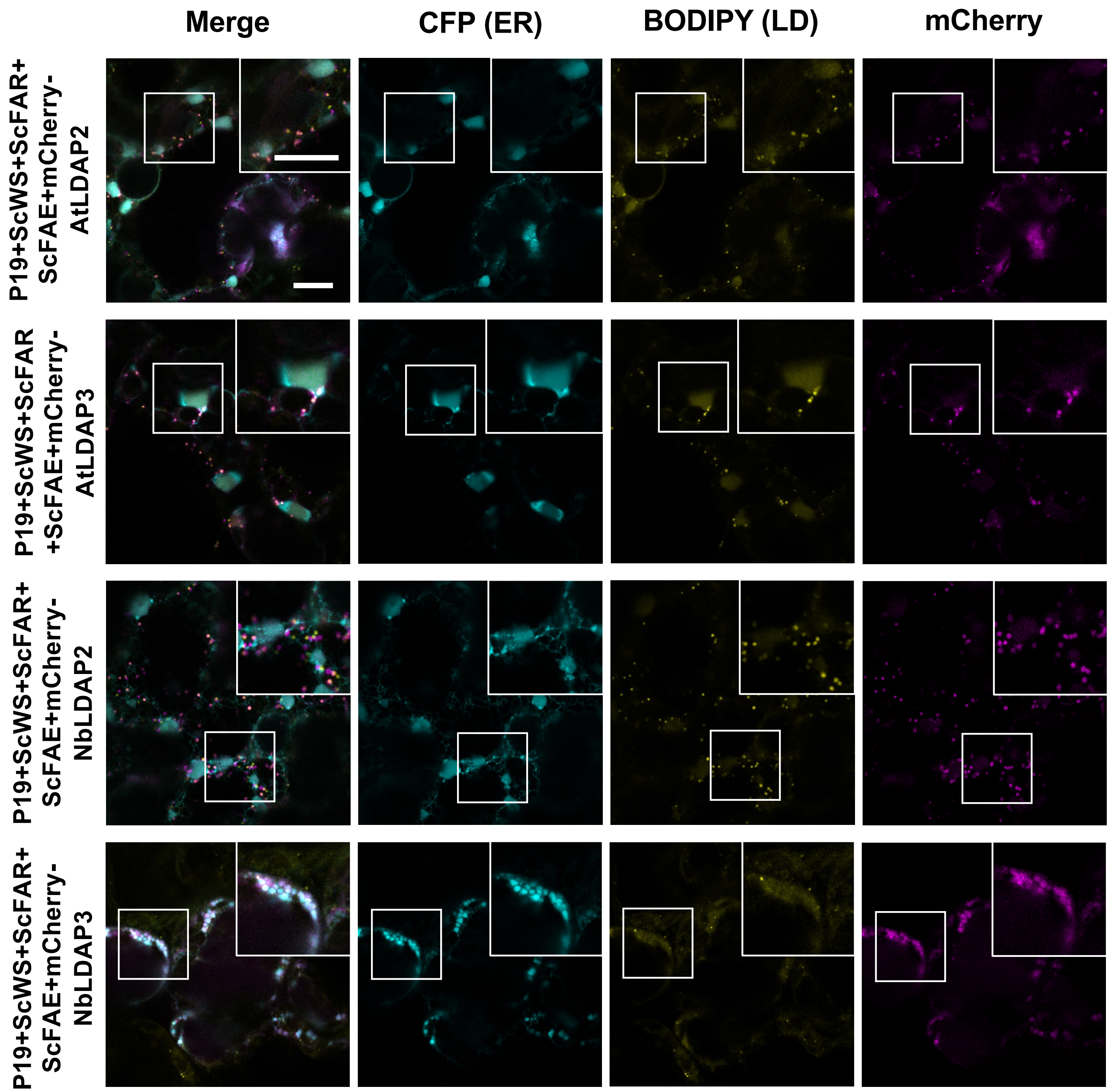

Supplementary Figure S4: Transient expression of LDAP isoforms from Arabidopsis and *N. benthamiana* do not correct WE-induced defects.

(Supports figure 3) Representative confocal laser scanning microscopy (CLSM) images of *N. benthamiana* leaves transiently expressing jojoba wax synthase (ScWS), jojoba fatty acyl-CoA reductase (ScFAR), a cyan fluorescent protein (CFP) tagged endoplasmic reticulum (ER) marker (CFP-HDEL) with either, N-terminal mCherry fluorescently tagged- *N. benthamiana* lipid droplet associated protein 2 (NbLDAP2), *N. benthamiana* lipid droplet associated protein 3 (NbLDAP3), Arabidopsis LDAP2 (AtLDAP2), or Arabidopsis LDAP3 (AtLDAP3). Samples were stained with BODIPY (493/503) to visualize neutral lipids. Magnified inserts highlight regions of wax ester (WE) induced ER defects. For each transient expression experiment *A. tumefaciens* harboring the P19 viral suppressor construct was added to suppress transgene silencing. All samples were stained and imaged (Zeiss LSM710 confocal laser scanning microscope, fitted with AIRYSCAN attachment) at 4 days post infiltration. Scale bar represents 10 μm and corresponds to all images. Transient expression of LDAP2 or LDAP3 from either Arabidopsis or *N. benthamiana* did not restore WE induced ER defects. Abbreviation: lipid droplet (LD).

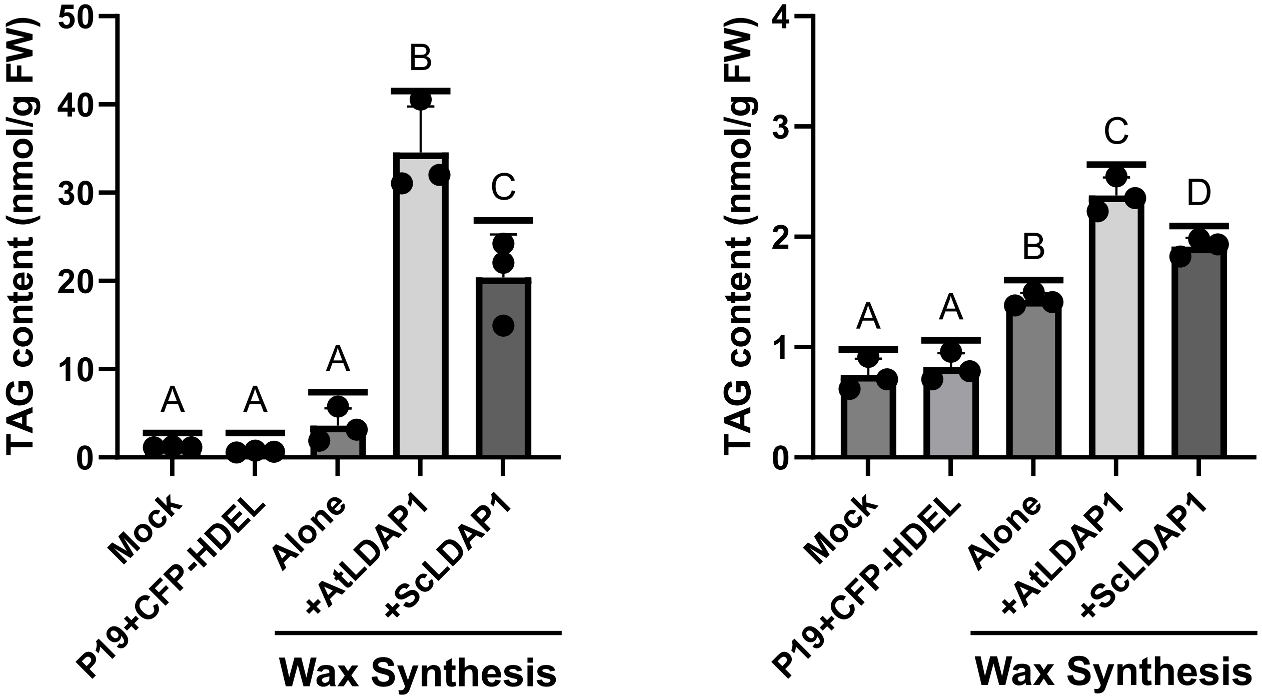

Supplementary Figure S5. Analysis of TAG distribution between isolated LDs and microsomes from infiltrated *N. benthamiana* leaves.

(Supports figure 4) Quantification of triacylglycerols (TAGs) from either lipid droplet (LD) or microsomal fractions isolated from infiltrated *N. benthamiana* leaves. “Wax Synthesis” samples include the expression of P19 (endogenous silencing suppressor), ScWS, ScFAR, and ScFAE collectively and in combination with either AtLDAP1, or ScLDAP1. Different letters indicate significant difference at $p \leq 0.05$, as determined by one-way ANOVA with Tukey’s post-test (n=3). Error bars correspond to standard deviation. Transient expression of heterologous genes was accomplished by agrobacterium mediated expression. For each transient expression experiment *A. tumefaciens* harboring the P19 viral suppressor construct was added to suppress transgene silencing. Samples were collected 4 days post infiltration for analysis. For each transient expression experiment *A. tumefaciens* harboring a jojoba fatty acid elongase (ScFAE) was included to elongate endogenous *N. benthamiana* fatty acids to 20 and 22 carbon in length to mimic endogenous jojoba fatty acids. Abbreviation: fresh weight (FW).

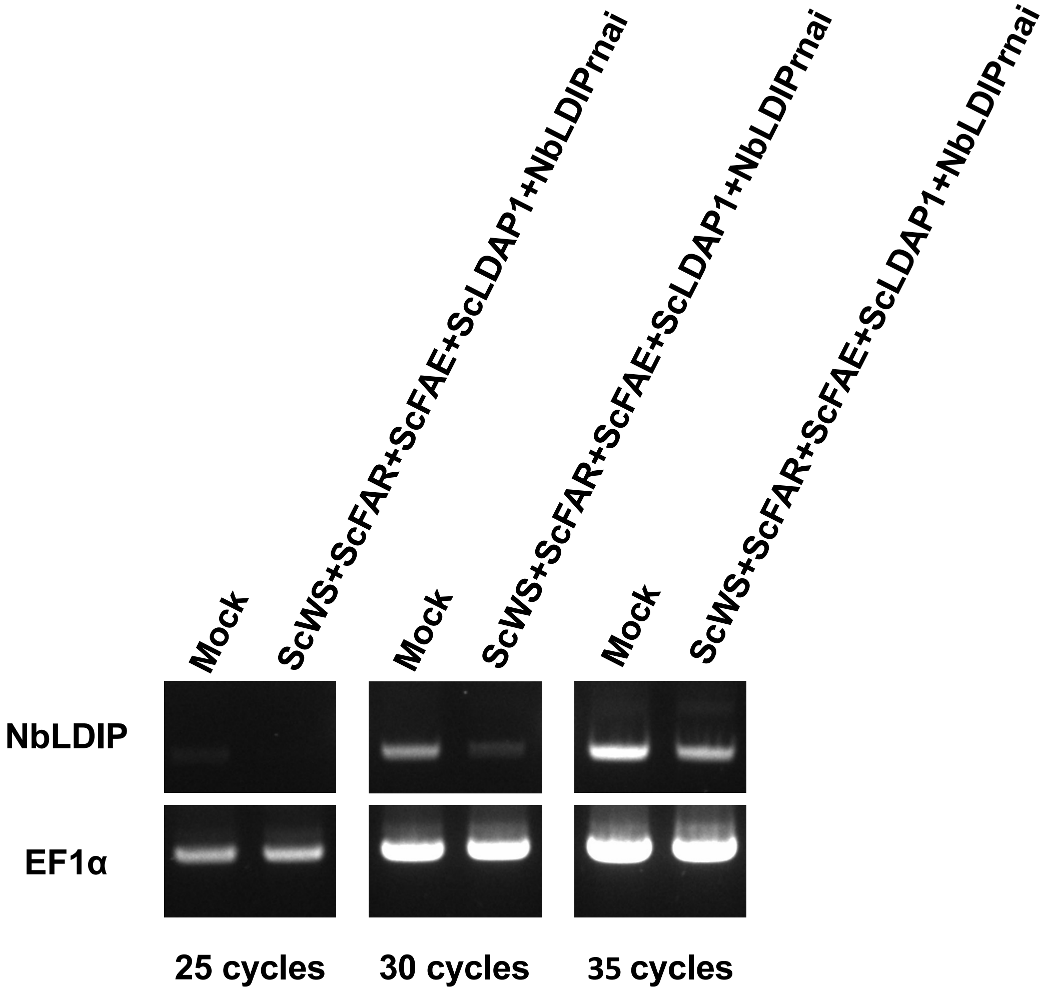

Supplementary Figure S6: Confirmation of NbLDIP silencing in *N. benthamiana* leaves by qualitative RT-PCR.

(Supports figure 5) Qualitative RT-PCR from RNA extracted from either mock infiltrated, or listed construct infiltrated *N. benthamiana* leaves. Primers specific to endogenous *N. benthamiana* lipid droplet associated protein interacting protein (NbLDIP) and not the region of the sequence used for NbLDIP suppression, were used for amplification at 25, 30, and 35 cycles (Supplementary Table S2). *N. benthamiana* elongation factor 1α (EF1α) was used as a reference gene with its primers listed in Supplementary Table S2. The top row of gel images corresponds to amplification of NbLDIP. The bottom row of gel images corresponds to amplification of EF1α.

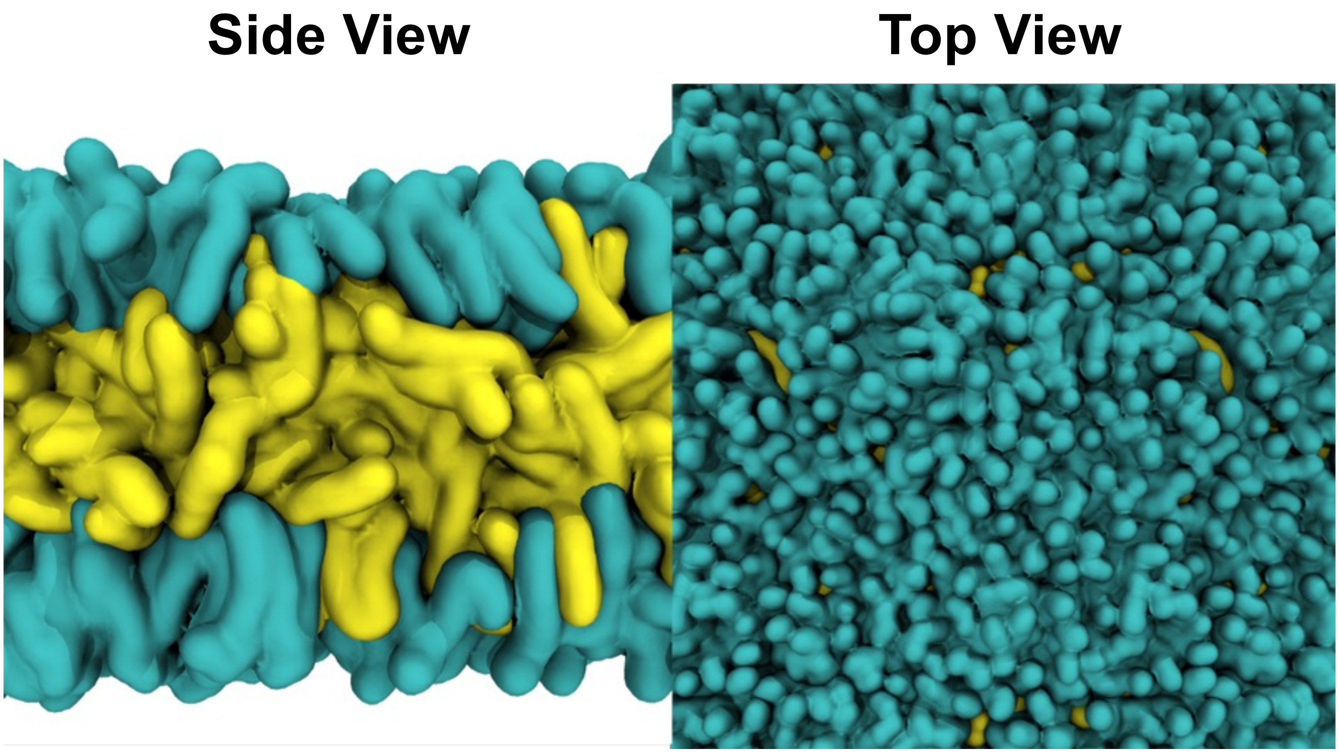

Supplementary Figure S7: Emergence of WE over the LD membrane surface.

(Supports figures 6,7) Side profile, and surface profile snapshots of modeled lipid droplet (LD) membrane surface. Cyan surface representation depicts the phospholipids of LD and yellow surface representation is for the wax esters (WEs). (See also simulations for both jojoba and N. tabacum lipid compositions, Supplementary Videos 1, 2).

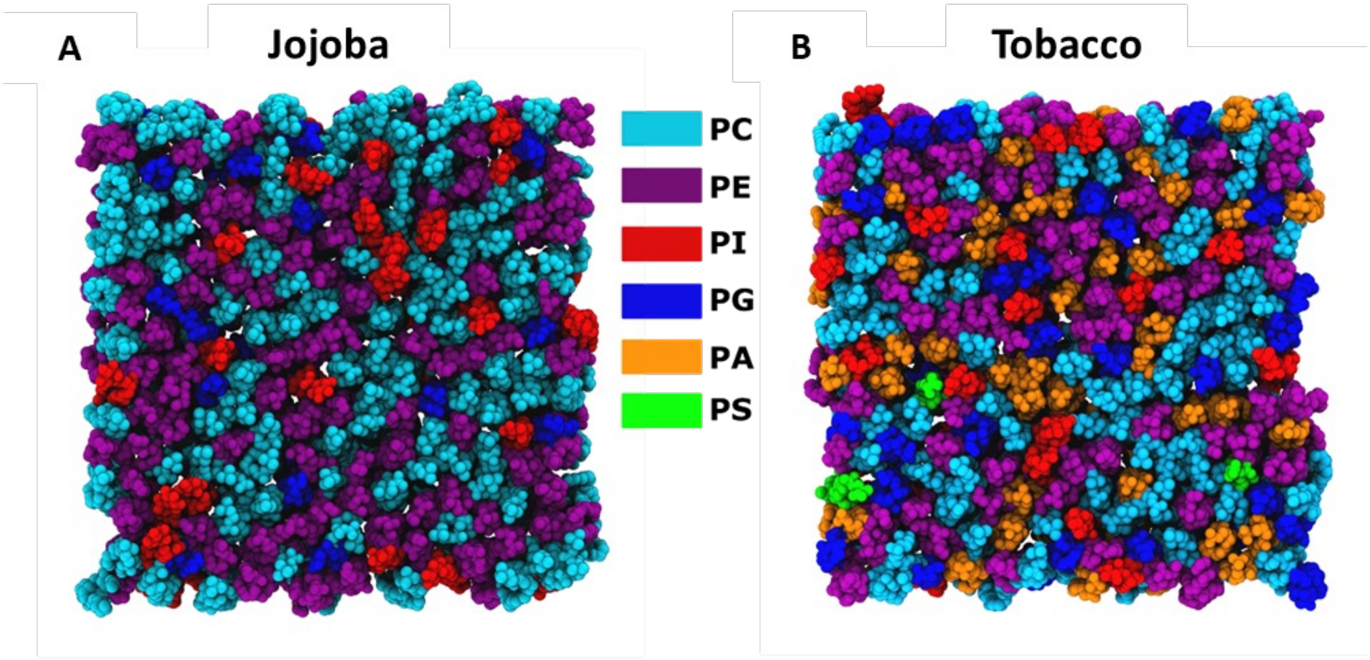

Supplementary Figure S8: Overhead view of the membrane compositions of the head groups in jojoba and *N. tabacum*- like systems.

(Supports figures 6,7) Atoms of each head group are assigned a unique color, cyan for phosphatidylcholine (PC), purple for phosphatidylethanolamine (PE), red for phosphatidylinositol (PI), blue for phosphatidylglycerol (PG), mustard for phosphatidic acid (PA) and light green for phosphatidylserine (PS).

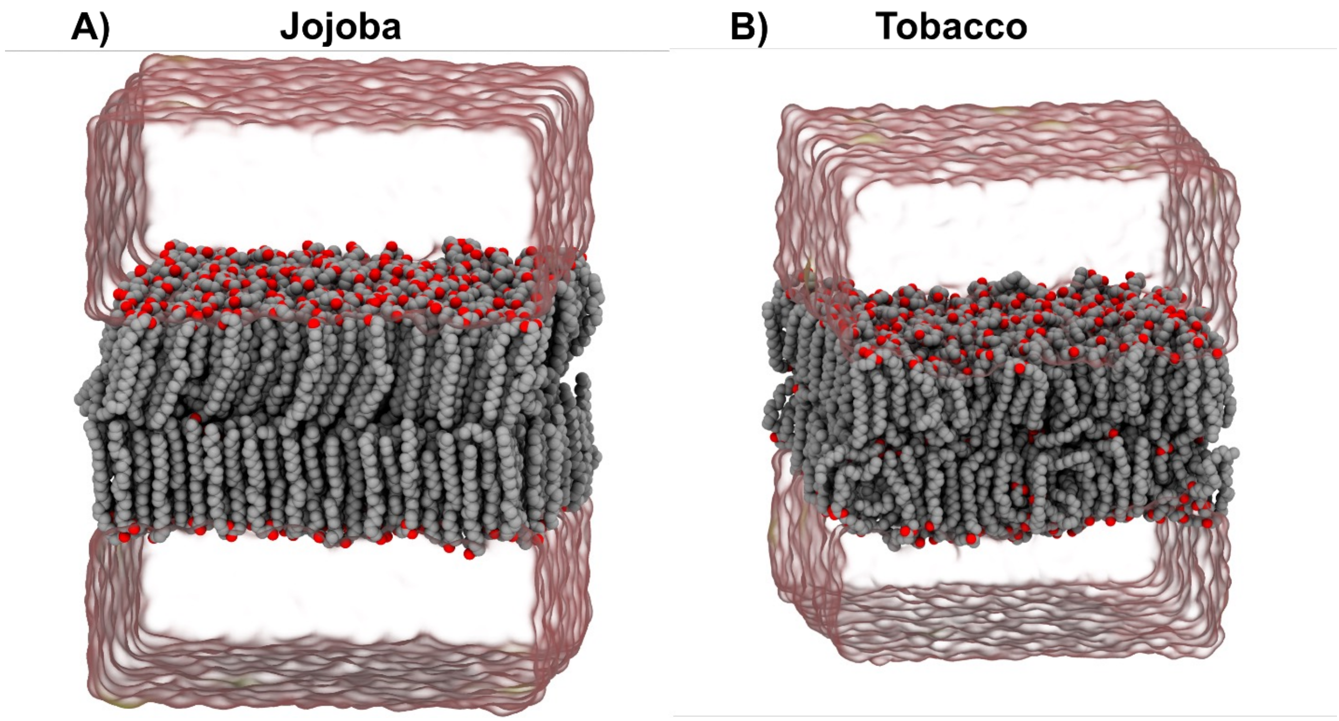

Supplementary Figure S9: Conformation of WEs in jojoba and *N. tabacum*- like membrane systems.

(Supports figures 6,7) The glass bubble representation corresponds to solvent molecules. Van Der Waal (VDW) representation corresponds to wax esters (WEs), red color beads correspond to oxygens, and grey color beads correspond to carbon atoms. Hydrogens are omitted for ease of visualization.

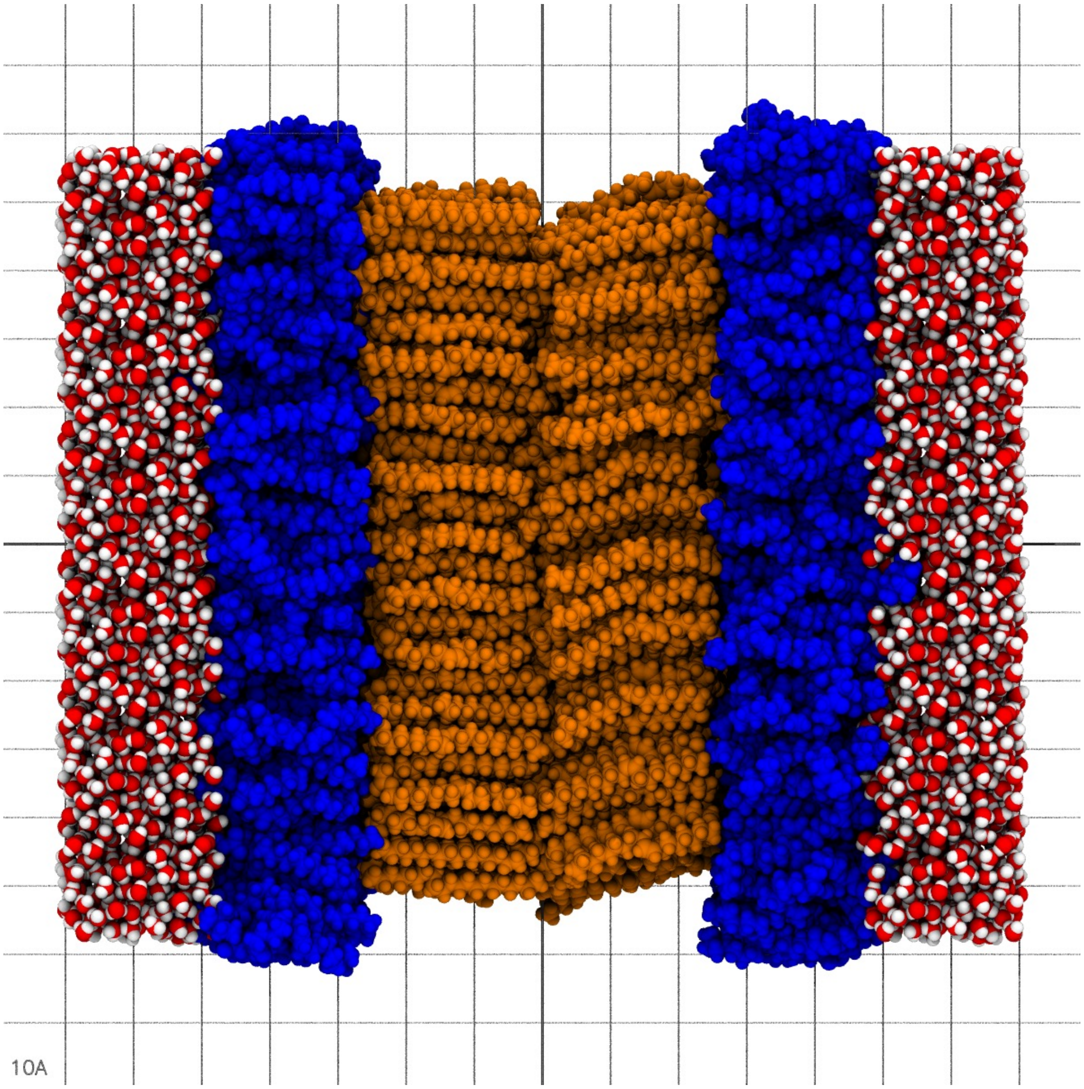

Supplementary Figure S10: Insertion of WEs inside a membrane bilayer to mimic the LD monolayer.

(Supports figures 6,7) Water is represented as red and white Van Der Waal (VDW) representation where red represents oxygen and white hydrogen. The membrane layers are represented with blue color and wax ester (WE) bilayer in mustard color in the VDW representation.

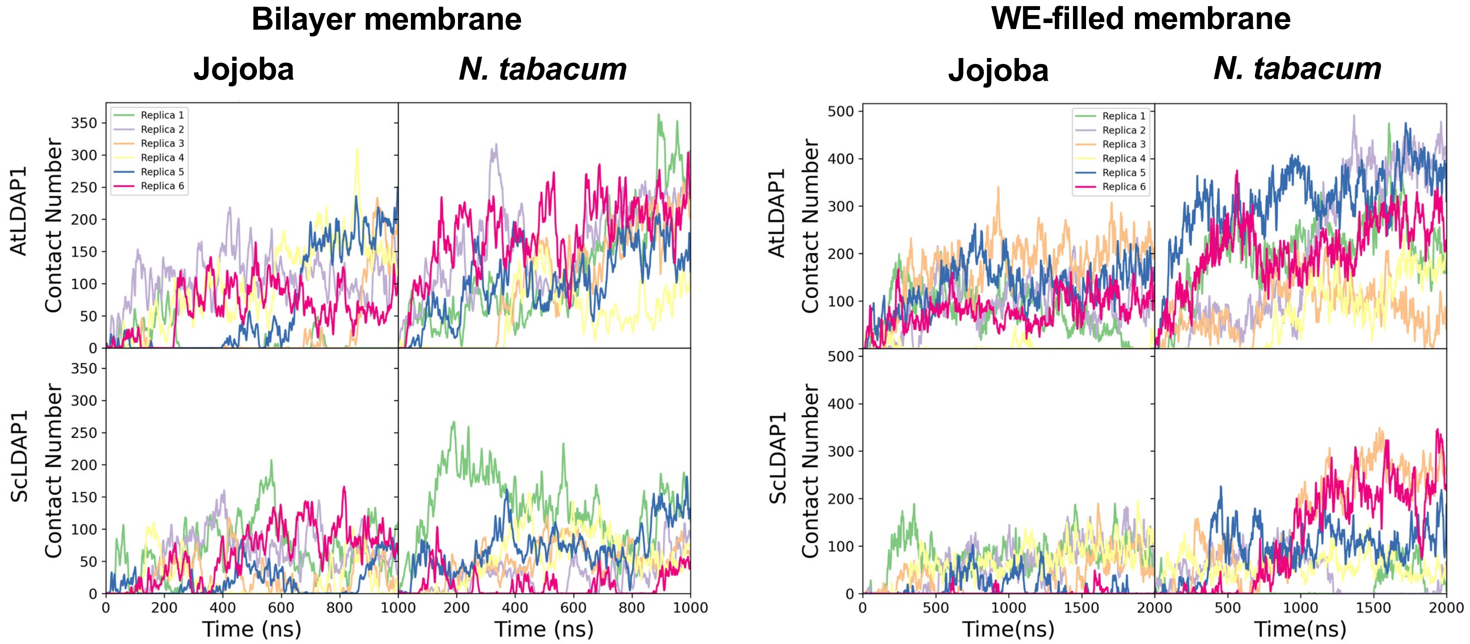

Supplementary Figure S11: Time based contacts plot of Arabidopsis and jojoba LDAP1 protein with bilayer membranes and WE-filled membranes.

(Supports figures 6,7) Graphical representations of the number of cumulative residue contacts to a jojoba or *N. tabacum-* like bilayer or wax ester (WE)-filled monolayer over the course of each simulation. Each colored line represents a different simulation with a different starting conformation. Data extracted from multiple simulations - Supplementary Video 3-8, MDS of Arabidopis lipid droplet associated protein 1 (AtLDAP1) with a jojoba like bilayer; Supplementary Video 9-14, molecular dynamic simulations (MDS) of jojoba LDAP1 (ScLDAP1) with a jojoba- like bilayer; Supplementary Video 15-20, MDS of AtLDAP1 with a jojoba- like WE-filled monolayer; Supplementary Video 21-26, MDS of ScLDAP1 with a jojoba- like WE-filled monolayer; Supplementary Video 27-32, MDS of AtLDAP1 with a *N. tabacum-* like bilayer; Supplementary Video 33-38, MDS of ScLDAP1 with a *N. tabacum-* like bilayer; Supplementary Video 39-44, MDS of AtLDAP1 with a *N. tabacum-* like phospholipid monolayer, filled with *N. benthamiana-* like WEs; Supplementary Video 45-50, MDS of ScLDAP1 with a *N. tabacum-* like phospholipid monolayer.

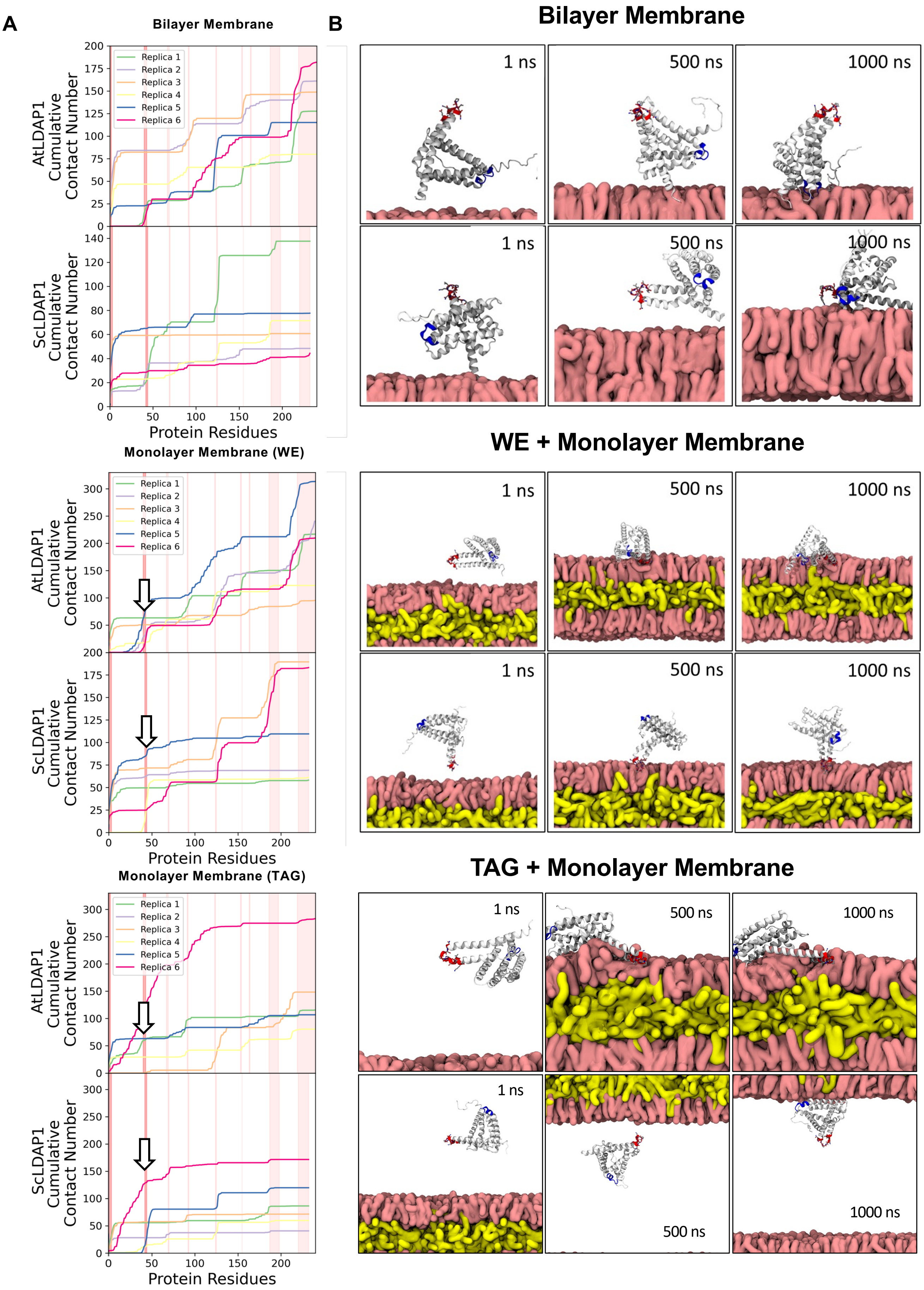

A
Bilayer Membrane
B
Bilayer Membrane
AtLDAP1 Cumulative Contact Number
ScLDAP1 Cumulative Contact Number
Replica 1
Replica 2
Replica 3
Replica 4
Replica 5
Replica 6
Protein Residues
1 ns
500 ns
1000 ns
Monolayer Membrane (WE)
WE + Monolayer Membrane
Monolayer Membrane (TAG)
TAG + Monolayer Membrane

Supplementary Figure S12: Simulated interactions of AtLDAP1 and ScLDAP1 with *N. tabacum*-like bilayer, WE- and TAG-filled monolayer.

A) (Supports figures 6,7) Average contact number per lipid droplet associated protein 1 (LDAP1) protein residue to *N. tabacum*- like phospholipid monolayer filled with *N. benthamiana* like- wax esters (WEs), or *N. tabacum*- like triacylglycerols (TAGs) (Supplementary Table S1), reported as a cumulative sum. Average contact number was calculated based on the bound conformation where the contact number of each residue to the membrane was greater than 25. Different colored lines represent a different simulation trajectory. White arrows denote the region that makes marked contacts with the surface. This region is colored dark red in the snapshots shown in (B). See simulations, Supplementary Video 3-8, molecular dynamic simulation (MDS) of Arabidopsis LDAP1 (AtLDAP1) with a jojoba- like bilayer; Supplementary Video 9-14, MDS of jojoba LDAP1 (ScLDAP1) with a jojoba- like bilayer; Supplementary Video 15-20, MDS of AtLDAP1 with a jojoba- like WE-filled monolayer; Supplementary Video 21-26, MDS of ScLDAP1 with a jojoba- like WE-filled monolayer; Supplementary Video 53-58, MDS of AtLDAP1 with a jojoba- like TAG-filled LD membrane; Supplementary Video 59-64, MDS of ScLDAP1 with a jojoba- like TAG-filled LD membrane.

B) Representative snapshots at 1 ns, 500 ns, and 1000 ns, of LDAP1s simulated with the WE- and TAG- filled monolayer. The red highlighted region in snapshots corresponds to the loop identified to directly insert into the monolayer. The blue region is the amphipathic α-helix identified through mutation analysis. See simulations, Supplementary Video 27-32, MDS of AtLDAP1 with a *N. tabacum*- like bilayer; Supplementary Video 33-38, MDS of ScLDAP1 with a *N. tabacum*- like bilayer; Supplementary Video 39-44, MDS of AtLDAP1 with a *N. tabacum*- like phospholipid monolayer, filled with *N. benthamiana*- like WEs; Supplementary Video 45-50, MDS of ScLDAP1 with a *N. tabacum*- like phospholipid monolayer, filled with *N. benthamiana*- like WEs; Supplementary Video 65-70, MDS of AtLDAP1 with a *N. tabacum*- like TAG-filled monolayer; Supplementary Video 71-76, MDS of ScLDAP1 with a *N. tabacum*- like TAG-filled monolayer.

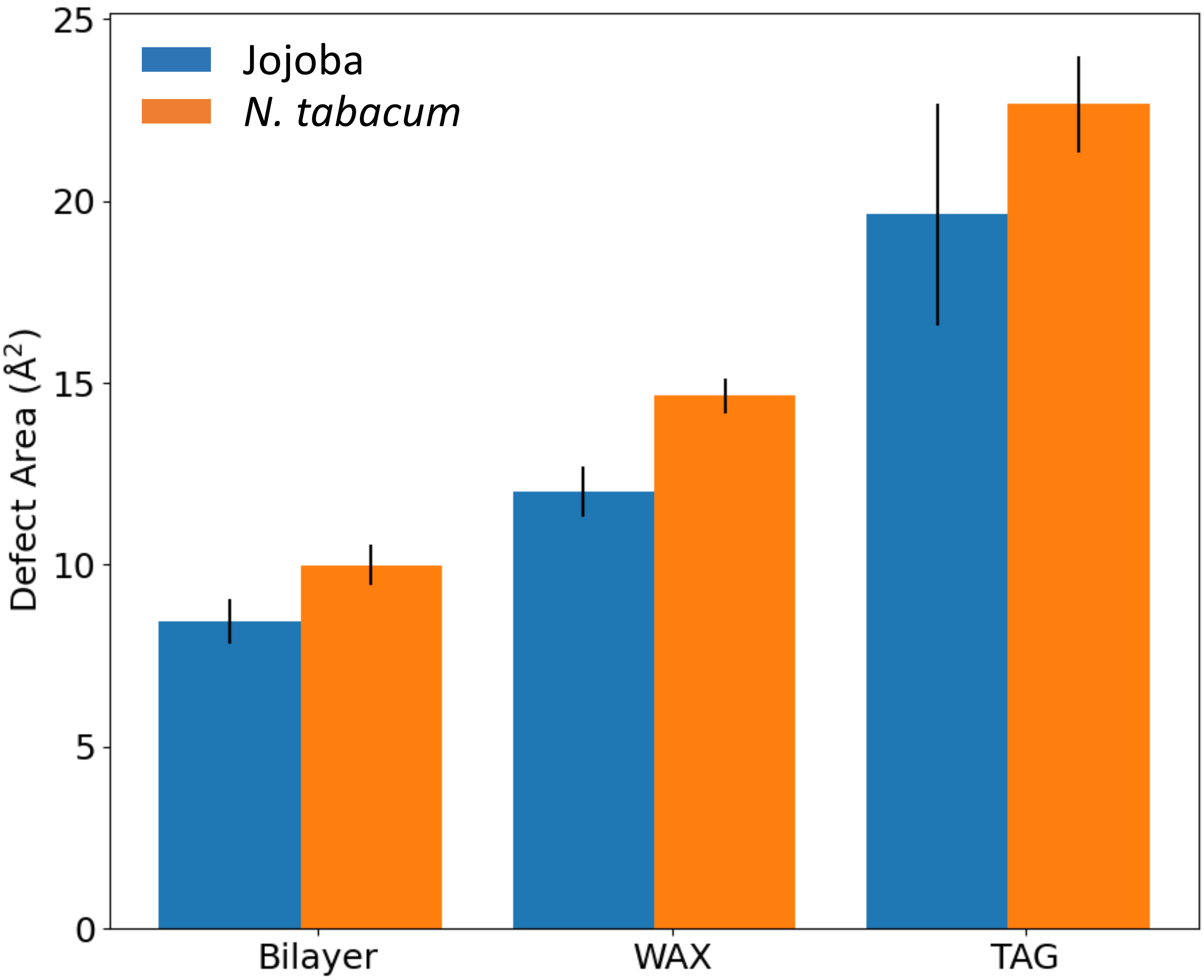

Supplementary Figure S13: Average area of phospholipid membrane defects observed within bilayer, WE-filled, and TAG-filled monolayer simulations.

(Supports figures 6,7) The total defect area quantification of both WE and TAG filled monolayer simulations as determined by PackMem bioinformatics tool. Defect area (Å^2) was calculated as the average sum of the defect area throughout each monolayers simulation after allowing the simulation to equilibrate for 100ns. Measurements were taken every 100ps of the simulation for a total of 9000 measurements. Error bars correspond to standard deviation in defect area (Å^2).

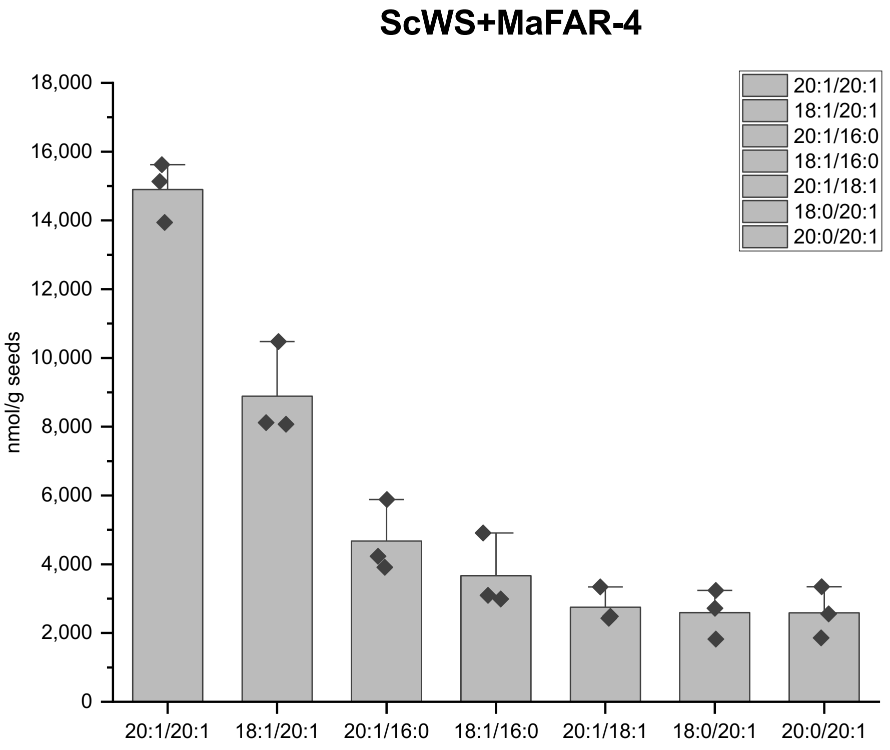

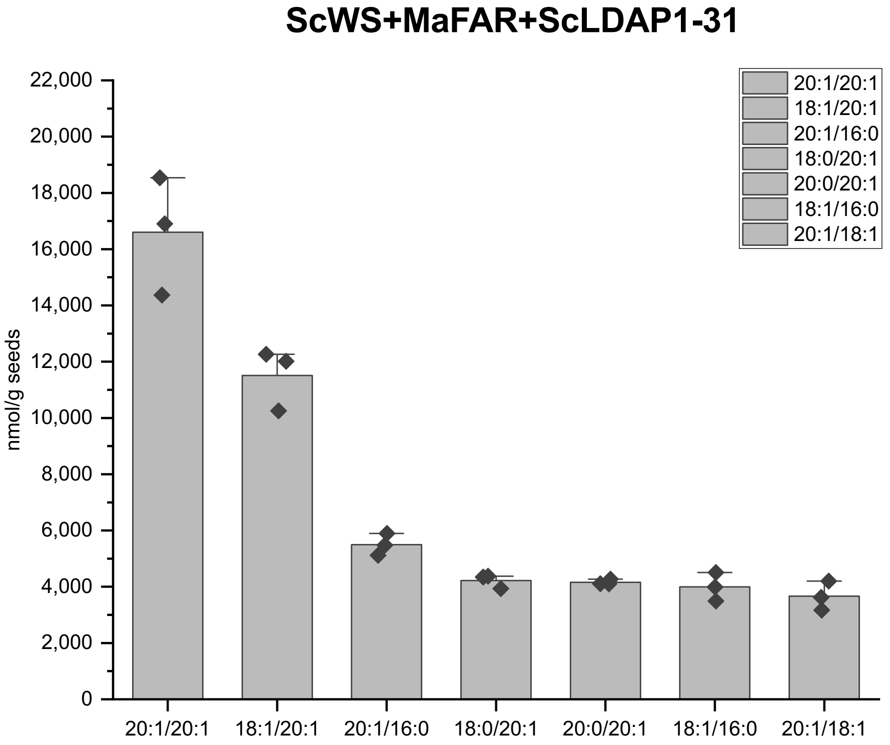

Supplementary Figure S14: ScLDAP1 expression does not affect WE profiles in Arabidopsis seeds expressing WE machinery.

(Supports figure 11) Molecular species of wax esters (WEs) in seeds of representative Arabidopsis lines expressing either jojoba wax synthase (ScWS) and *Marinobacter aquaeolei* fatty acyl-CoA reductase (MaFAR) together (ScWS+MaFAR-4), or with jojoba lipid droplet associated protein 1 (ScLDAP1) (ScWS+MaFAR+ScLDAP1-31). WE composition was determined by nano-ESI–MS/MS. Shown is the mean content (+ SD; n=3) of the top seven WE molecular species (alcohol moiety/acyl moiety).

A

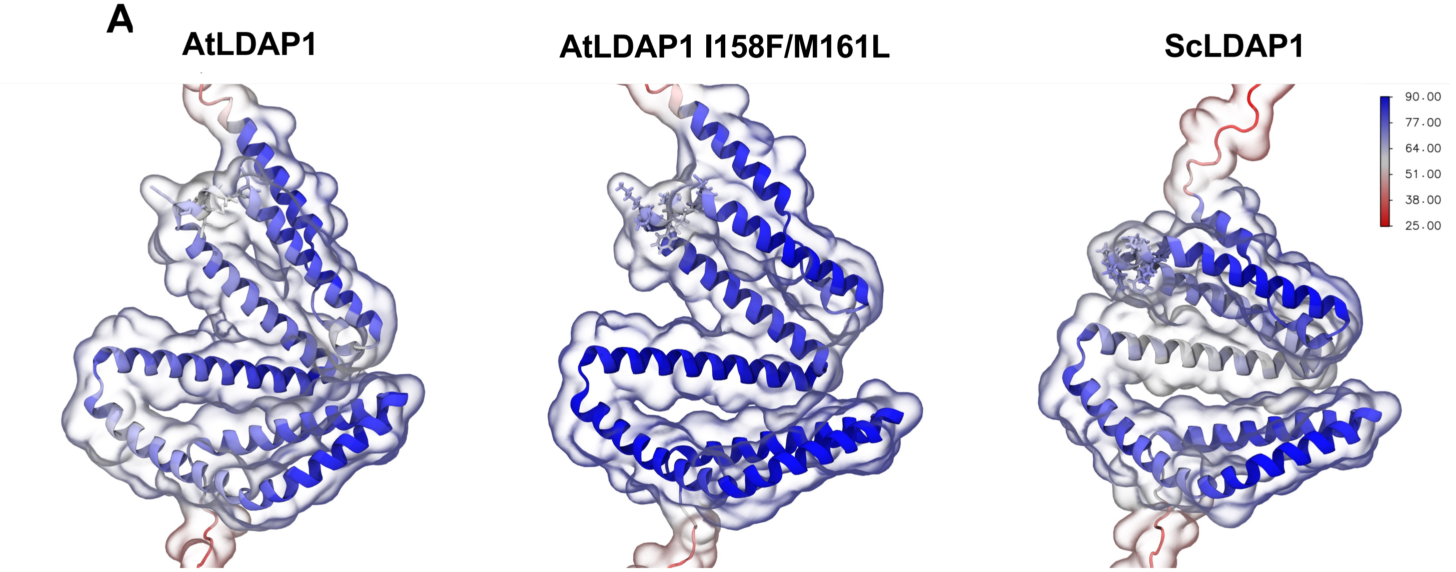

B

```
Arabidopsis     ME-TEKKNSKEVALKHLAFVRIATIHILASVSNLYEYAKQNSGPLKSAVEKVEGAVTTVV   59
Jojoba          METTTSTEMKKRELKHLGFVKVAAVNTIMCLSSVYEYAKQNSGPLKSTVGAVEGAVTTVV   60
                ** * ..: *:  ****.**::*::: : .:*.:*************:*  *********

Arabidopsis     TPVYQKFKDVPDSLLVFLDHKVGEVSYKFDEHAPPMAKKVVNQAHVLIYKATEKAQSFVK   119
Jojoba          SPVYEKFKGVPDDLLGFLDEKVGVASEKFDEHAPPLAKHLVSKTHDIIVKASQVTQDLIN   120
                :***:***.***.** ***.*** .* ********:**::*.::* :* **:: :*.:::

Arabidopsis     EARTGGPKAAFNYAATEYKFFVVTNSVKVWAKLNQYKPIHAMGDKALPVAAHFSSRYNDL   179
Jojoba          HVKTDGPRAAAHYAASESKQLVFIQSAKIWTRLDQIPPFHMLADIAVPTAAHWSEKYNKV   180
                ..:*.**:** :***:* * :*. :*.*:*::*:*  *:* :.* *:*.***:*.:**.:

Arabidopsis     VTDMTNMGYSLVGYLPLVPVDDIVKAYEKEDARRKKGGDTAGKKGETTDAADGDKSSSDS   239
Jojoba          VVDMSCKGHVIFSYLPLVPIDDIAQAFKPAGSAKEEEGEDGAAPAHEA------QSQSD*   233
                *.**:  *: :..******:***.:*::  .: ::: *: ..  .. :      :*.**

Arabidopsis     E      240
Jojoba          -      233
```

Supplementary Figure 15: Alphafold2 modeling, and Sequence Alignment of AtLDAP1 and ScLDAP1.

A) (Supports figure 10) Alphafold-predicted structural models of Arabidopsis lipid droplet associated protein 1 (AtLDAP1), AtLDAP1 I158F/M161L, and jojoba LDAP1 (ScLDAP1). Red to blue coloring of models indicates Alphafold pLDDT score.

B) (Supports figure 10) Sequence alignment of the deduced peptide sequences of LDAP1 from A. thaliana (Arabidopsis), and S. chinensis (jojoba). Numbers to the right of the sequences indicate residue position in the respective peptide. Asterisks underneath residue indicate conservation of residues between the two peptides at that position. Colons underneath residues indicate conservation of residues with similar properties at that position. Periods indicate conservation of residue with weakly similar properties at that position.

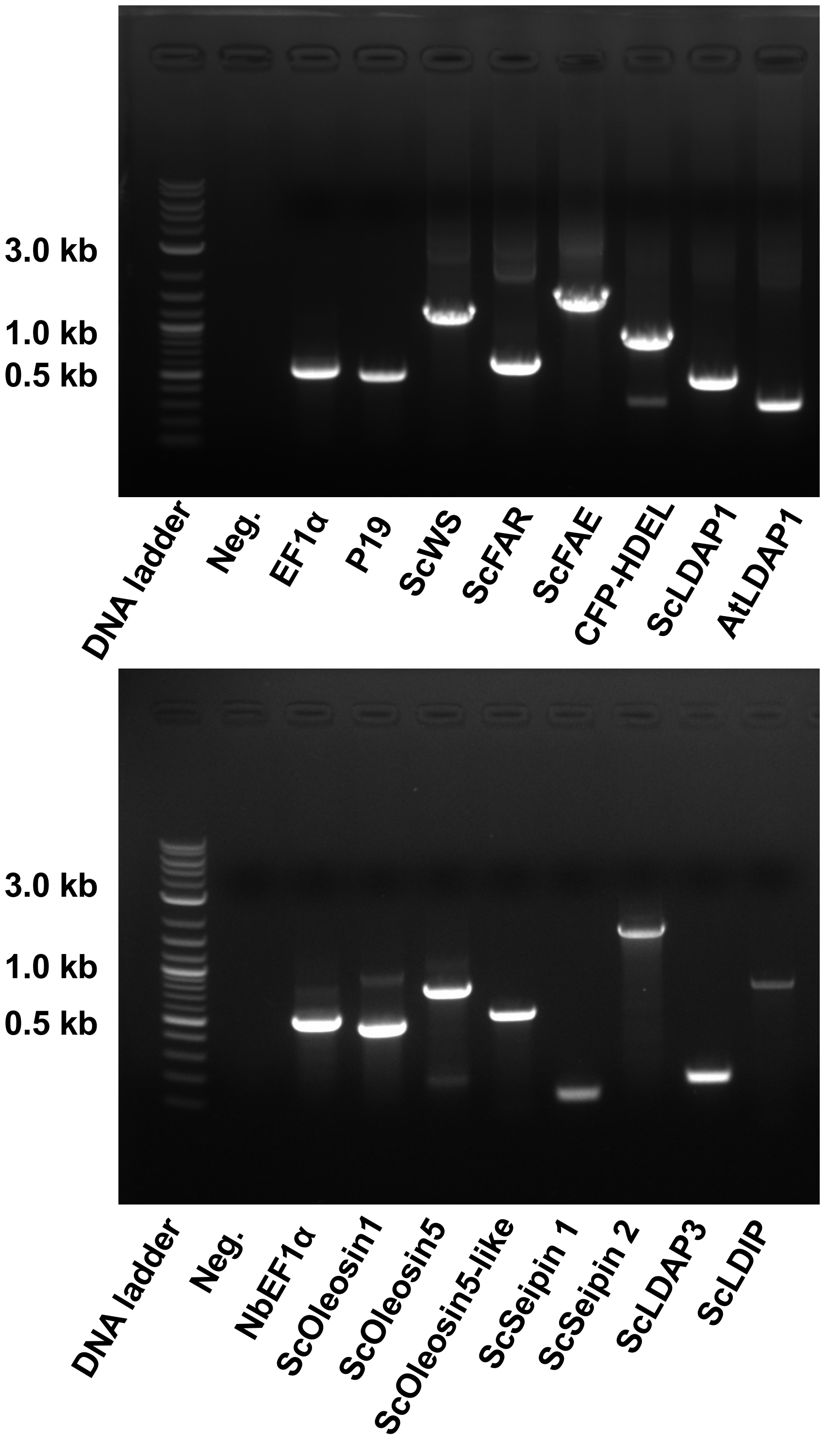

Supplementary Figure S16: Confirmation of transgene expression by qualitative RT-PCR.

(Supports figures 1-4, 8,9) Transient expression of transgenes (P19, jojoba wax synthase (ScWS), jojoba fatty acyl-CoA reductase (ScFAR), jojoba fatty acid elongase (ScFAE), cyan fluorescent protein-HDEL (CFP-HDEL) (ER marker), jojoba lipid droplet associated protein 1 (ScLDAP1), Arabidopsis LDAP1 (AtLDAP1), jojoba Oleosin1 (ScOleosin1), jojoba Oleosin5 (ScOleosin5), jojoba Oleosin5-like (ScOleosin5-like), jojoba Seipin1 (ScSeipin1), jojoba Seipin2 (ScSeipin2), jojoba lipid droplet associated protein 2 (ScLDAP2), and jojoba LDAP interacting protein (ScLDIP)) were confirmed by qualitative RT-PCR. Sequences of primers used in PCR included in Supplementary Table S2. Elongation factor 1 α (EF1α) was used as a reference gene for each sample. Negative control (Neg.) reactions with EF1α specific primers using RNA samples not treated with reverse transcriptase were shown to confirm no DNA contaminations in the samples. Abbreviation: kilobase (kb).

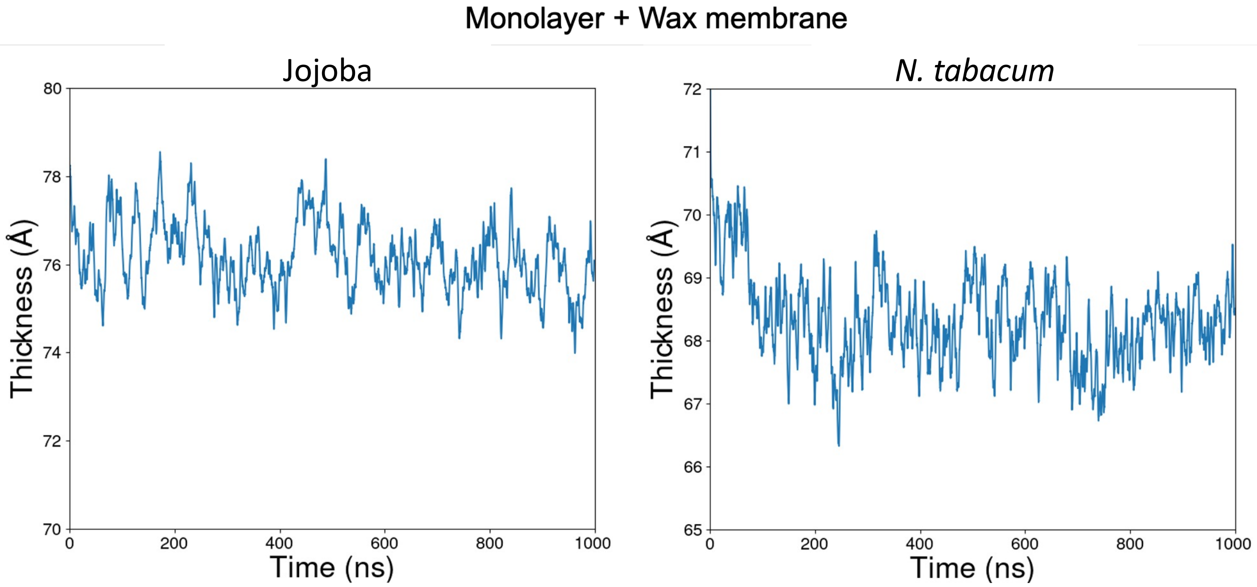

Supplementary Figure S17: Thickness of membrane with WEs measured by the phosphate-to-phosphate distance from the two monolayers.

(Supports figures 6,7) To ensure the stability of the monolayer systems, stability of, and deformations within, the lipid droplet analog were tracked. The stability of the membrane system was checked by measuring the thickness of the monolayer and wax ester (WE) by finding the average phosphate distance of the two monolayers (jojoba and *N. tabacum*) distributed on both sides of the wax ester. This distance shows there is a fluctuation of about 2 Å in the thickness of monolayer and wax ester for jojoba and *N. tabacum,* suggesting that the monolayer systems are stable.

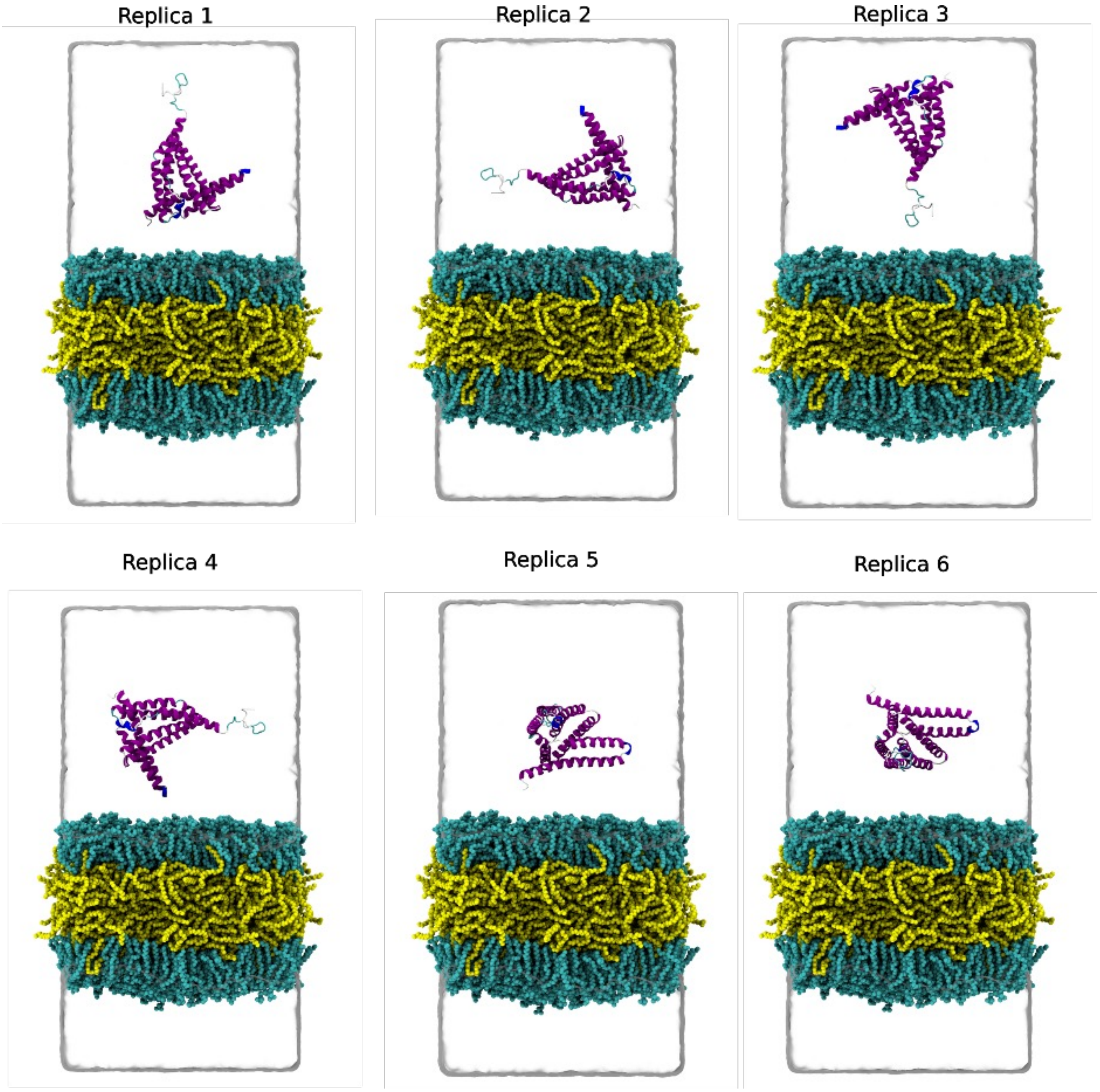

Supplementary Figure S18: Depiction of the simulation box along with the six-replica initial protein starting positions.

(Supports figure 6,7) The first 4 replicas were rotated around the x-axis 0, 90 ,180 and 270 and two were rotated around the y axis at 90 and – 90. In this depiction, the monolayer membrane as cyan Van Der Waal (VDW) representation, wax ester (WE) as yellow VDW representation and lipid droplet associating protein 1 (LDAP1) protein as cartoon representation and solvent as white glass surface representation. Hydrogen atoms are omitted from the VDW representation for cleaner depiction of the simulation system.
